# Programming Skeletal Muscle Metabolic Flexibility in Offspring of Male Rats in Response to Maternal Consumption of Slow Digesting Carbohydrates during Pregnancy

**DOI:** 10.3390/nu12020528

**Published:** 2020-02-19

**Authors:** Rafael Salto, María D. Girón, Manuel Manzano, María J. Martín, Jose D. Vílchez, Pilar Bueno-Vargas, Elena Cabrera, Mónica Pérez-Alegre, Eloisa Andujar, Ricardo Rueda, Jose M. Lopez-Pedrosa

**Affiliations:** 1 Department of Biochemistry and Molecular Biology II, School of Pharmacy, University of Granada, Campus de Cartuja, 18071 Granada, Spain; mgiron@ugr.es (M.D.G.); e.damaso@go.ugr.es (J.D.V.); elenacc_20@hotmail.com (E.C.); 2 Abbott Nutrition R&D, Abbott Laboratories, 18004 Granada, Spain; manuel.manzano@abbott.com (M.M.); chmmmj@yahoo.com (M.J.M.); pilar.bueno@abbott.com (P.B.-V.); ricardo.rueda@abbott.com (R.R.); jose.m.lopez@abbott.com (J.M.L.-P.); 3 Centro Andaluz de Biología Molecular y Medicina Regenerativa-CABIMER, Universidad de Sevilla-CSIC-Universidad Pablo de Olavide, 41704 Seville, Spain; monica.perez@cabimer.es (M.P.-A.); eloisa.andujar@cabimer.es (E.A.)

**Keywords:** early programming, skeletal muscle, muscle differentiation, insulin-resistant pregnancy, metabolic flexibility, slow digesting carbohydrates

## Abstract

Skeletal muscle plays a relevant role in metabolic flexibility and fuel usage and the associated muscle metabolic inflexibility due to high-fat diets contributing to obesity and type 2 diabetes. Previous research from our group indicates that a high-fat and rapid-digesting carbohydrate diet during pregnancy promotes an excessive adipogenesis and also increases the risk of non-alcoholic fatty liver disease in the offspring. This effect can be counteracted by diets containing carbohydrates with similar glycemic load but lower digestion rates. To address the role of the skeletal muscle in these experimental settings, pregnant rats were fed high-fat diets containing carbohydrates with similar glycemic load but different digestion rates, a high fat containing rapid-digesting carbohydrates diet (HF/RD diet) or a high fat containing slow-digesting carbohydrates diet (HF/SD diet). After weaning, male offspring were fed a standard diet for 3 weeks (weaning) or 10 weeks (adolescence) and the impact of the maternal HF/RD and HF/SD diets on the metabolism, signaling pathways and muscle transcriptome was analyzed. The HF/SD offspring displayed better muscle features compared with the HF/RD group, showing a higher muscle mass, myosin content and differentiation markers that translated into a greater grip strength. In the HF/SD group, metabolic changes such as a higher expression of fatty acids (FAT/CD36) and glucose (GLUT4) transporters, an enhanced glycogen content, as well as changes in regulatory enzymes such as muscle pyruvate kinase and pyruvate dehydrogenase kinase 4 were found, supporting an increased muscle metabolic flexibility and improved muscle performance. The analysis of signaling pathways was consistent with a better insulin sensitivity in the muscle of the HF/SD group. Furthermore, increased expression of genes involved in pathways leading to muscle differentiation, muscle mass regulation, extracellular matrix content and insulin sensitivity were detected in the HF/SD group when compared with HF/RD animals. In the HF/SD group, the upregulation of the *ElaV1*/*HuR* gene could be one of the main regulators in the positive effects of the diet in early programming on the offspring. The long-lasting programming effects of the HF/SD diet during pregnancy may depend on a coordinated gene regulation, modulation of signaling pathways and metabolic flexibility that lead to an improved muscle functionality. The dietary early programming associated to HF/SD diet has synergic and positive crosstalk effects in several tissues, mainly muscle, liver and adipose tissue, contributing to maintain the whole body homeostasis in the offspring.

## 1. Introduction

Metabolic adaptations to nutrients supply is a well-known process. Humans have the capability to promote fat storage from other nutrients such as carbohydrates. The fat, carbohydrate content and glycemic index of the diet are able to induce long-term adaptations that affect the homeostasis and metabolic flexibility of the body.

While these diet-induced alterations are well-known in cases of diabetes or obesity in adulthood, less knowledge has been gathered regarding the influence of the diet during the perinatal period in the short- and long-term adaptations of the offspring. In a previous work, we have shown [1] that the offspring of rats fed a high-fat diet containing slow-digesting carbohydrates (HF/SD) during pregnancy seemed to be protected against an increase in adipose tissue mass during adolescence. The HF/SD animals had reduced body fat mass and lower levels of cholesterol and triacylglycerols in plasma when compared to their counterparts whose mothers were fed a high-fat and rapid-digesting carbohydrate diet (HF/RD) during gestation. Furthermore, under the same experimental setting [2], livers of the HF/RD group showed a decrease in the Protein kinase B/Akt (Akt)/insulin signaling together with changes in the carbohydrate response element and sterol regulatory element binding proteins nuclear expression. All of them translated into an increase in fat liver storage and, therefore, non-alcoholic fatty liver disease risk. These changes were corrected by feeding a HF/SD diet during pregnancy.

Skeletal muscle is the organ that enables movement and force. Skeletal muscle represents 40–50% of body mass. It has the capability to uptake glucose, store it as glycogen and burn it for energy. Also, muscle can use fatty acids (FA) as metabolic fuels, having the ability to switch fuels in a regulated way. This capability is tightly regulated by insulin action as well as by exercise [3]. Therefore, a well-adjusted regulation is needed not only to promote muscle mass development, but also to facilitate body homeostasis in coordination with liver and adipose tissue metabolism [4]. Alterations in the growth and development of the muscle during the perinatal period could result not only in muscle mass deficits but also in systemic metabolic dysfunction [5,6].

On the other hand, pregnancy is an essential period for the growth and functionality of muscle development [7], since skeletal muscle fibers’ number is usually set by birth defining adult muscle mass [6]. The nutrition during pregnancy and the postnatal period is one of the main determinants of the muscle metabolism and performance in the offspring. Most of the muscle programming studies have been carried out in models of severe maternal under-nutrition, that lead to a decrease of skeletal muscle mass and strength in the offspring. However, albeit there are studies showing harmful effects of maternal obesity and diabetes on the muscle performance of the offspring [8], the modulation that carbohydrates nature of prenatal high-energy diets could exert in the offspring skeletal muscle health is not documented.

In this work, we have focused on the problem of programming the offspring of obese mothers as a result of the metabolic deregulation of mothers during pregnancy. With this model, we have tried to mimic this situation and animals were fed an obesogenic diet (high-fat, HF) before mating. Then, during pregnancy, we have analyzed the effects that two high-fat diets had in the muscle of the offspring at weaning and adolescence. One of these diets contains rapid-digesting carbohydrates (HF/RD) and the other contains slow-digesting carbohydrates (HF/SD). Our results highlight the relevance of the diet during the pregnancy period, since the offspring of the animals fed the HF/SD diet showed better muscle performance compared with the HF/RD group.

## 2. Materials and Methods

### 2.1. Animals

Female and male Sprague Dawley rats (10-week-old, Charles River Laboratories, Orleans Cedex, France) were maintained on a 12 h light/dark cycle at 23 ± 1 °C. Food and water were available ad libitum. All experimental procedures were carried out according to the ethical guidelines for animal experimentation provided by the Spanish National Research Council (RD 1201/10 October 2005).

### 2.2. Experimental Design

**Scheme 1:**
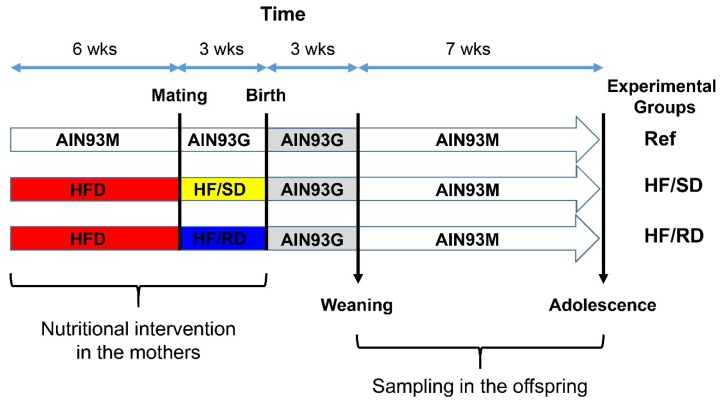
Experimental design. HFD: High Fat diet; HF/SD: High Fat Slow Digesting Carbohydrates diet; HF/RD: High Fat Slow Digesting Carbohydrates diet; Ref: Reference Group; Wks: Weeks

Thirty-six virgin female rats were housed independently during the premating period. They were randomly assigned to a Reference (Ref) group (*n* = 12) fed an AIN93M (AIN-93M is the second of two open formulations, published by the American Institute of Nutrition (AIN) committee in 1993 improving the AIN-76A standard diet. AIN-93M was structured to provide nutrients in concentrations required just to maintain adult rat or mouse populations, hence the “M” designation) diet [9] or a HF group (*n* = 24) receiving an obesogenic diet (HF; 20.5% fat, 24.2% protein, 41.5% carbohydrates and 7.9% fiber per weight) for 6 weeks. Then, female rats were mated with 13-week-old male rats and three days later, males were removed and female rats were randomly assigned either to a high-fat diet containing slow-digesting carbohydrates (HF/SD group) or to a high-fat diet containing rapid-digesting carbohydrates (HF/RD group). Rats fed AIN93M were changed into AIN93G (AIN-93G was the first of two open formulations published by the American Institute of Nutrition (AIN) committee in 1993 improving the laboratory standard, AIN-76A diet. AIN-93G is designed to accommodate the increased nutritional demands of rat or mouse growth as well as females who are pregnant or lactating, hense the “G” designation), the recommended rodent diet for gestation, lactation and growing [9]. All diets were prepared at Abbott Nutrition R&D (Research and Development) facilities. Composition of the three diets used during gestation has been described elsewhere [1,2] and is available as Supplementary Materials, Table S1. At delivery, all animals were fed AIN93G diets regardless of the diet consumed during pregnancy for 21 days (weaning rats), to guarantee that the expected effects in the offspring are due exclusively to a nutritional influence during gestation. On day 21 after delivery, all pups were changed onto AIN93M diet and housed in 4 male groups for seven additional weeks (adolescence rats). The individual body weights and food consumption were monitored regularly. A scheme of the experimental design is shown (Scheme 1).

Weaning and adolescent male rats of each group (*n* = 8) were anesthetized and euthanized by cardiac puncture. Gastrocnemius, soleus, tibialis anterior and extensor digitorum longus (EDL) muscles were immediately isolated, weighed and snap frozen in liquid nitrogen and kept at −80 °C for posterior analysis. Serum glucose, triacylglycerides and homeostasis model assessment (HOMA) are presented as Supplementary materials, Table S2.

### 2.3. Analysis of Strength

To measure grip strength, a Bioseb GRIP TEST Version 3+ apparatus was used (Bioseb, Chaville, France). For that purpose, both forepaws of the rat were placed on the front bar. Animals held by the tail grasped the bar and were then gently pulled away from it in a smooth, steady motion, until they released the bar. The strain gauge measured the force (N) required to break the animal’s grip. Three readings were taken for each animal and the average force required was recorded as the individual grip strength score of that rat. The measurements were carried out blindly regarding the experimental group.

### 2.4. Western Blot Analysis

Gastrocnemius lysates were obtained using the following lysis buffer: 50 mM Hepes pH 7.5, 150 mM NaCl, 1% Nonidet P-40, 10 mM NaF, 20 mM NaPPi, 1 mM MgCl2, 1 mM CaCl2, 20 mM β-glycerophosphate, 2 mM sodium orthovanadate, 2 mM ethylenediaminetetraacetic acid (EDTA), 2 mM phenylmethylsulfonyl fluoride (PMSF), 4 μg/L leupeptin. In brief, 150 mg of rat skeletal muscle were pulverized using a mortar and liquid nitrogen. The powder was transferred to a 2.0 mL eppendorf tube and then 500 µL of lysis buffer were added. The mix was homogenized for 15 s in Polytron at setting #4. The homogenate was centrifuged at 16,000× *g* for 15 min at 4 °C. The supernatant was transferred to a new eppendorf tube (1.5 mL) and sonicated for 15 s (cycle 0.5, amplitude 70%). Protein concentration of the sample was measured using the bicinchoninic acid method [10]. Specific antibodies against GLUT4 (Biogenesis Ltd., Poole, UK); myosin heavy chain F59 (Developmental Studies Hybridoma Bank, Iowa City, Iowa); total and phospho (Ser473)-PKB/Akt, total and phospho (Thr202/Tyr204)-p44/42 MAPK (ERK1/2), total and phospho (Thr172)-AMPKα2, total and phospho (Ser2448)-mTOR and MEF2D (Cell Signaling, Beverly, MA, USA); GLUT1, PKM2, PDK4, ATPase5B and UCP2 (Santa Cruz Biotechnology (Dallas, TX, USA); CD36/SR-B3 (Novus Biologicals, Centennial, CO, USA) were used. β-actin (Sigma-Aldrich, Saint Louis, MO, USA) was used as a load control.

### 2.5. Glycogen Content

Muscle glycogen was isolated as described [11]. Muscle homogenates (10%) were made in 0.03 N HCl and spread evenly on pieces of filter paper (Whatman 3M chromatography paper, 2.0 × 2.0 cm) in duplicate. The papers were dropped immediately into a beaker containing 66% Ethanol stirred gently by a rotating magnet screened from the papers by a wire mesh. The papers were subsequently washed three times for 40 min in 66% EtOH. Then, they were briefly rinsed with acetone and dried under a stream of warm air. The dried filter papers were cut into four pieces and placed in a tube containing 0.4 mL of 0.2 M acetate buffer, pH 4.8; 0.2 mg of amylo-α-1,4-α-1,6-glucosidase and H_2_O to a final volume of 2 mL. The vials were incubated for 90 min at 37 °C with gentle shaking. Appropriate controls were prepared by incubating aliquots of homogenate in acetate buffer minus amyloglucosidase. Glucose concentration in the incubated samples was determined by the glucose oxidase method.

### 2.6. Analysis of Pathways and Networks

Total RNA was extracted from muscles for each group. Purity and quality of isolated RNA were assessed by RNA 6000 Nano assay on a 2100 Bioanalyzer (Agilent Technologies, Santa Clara, CA, USA). The expression studies were performed by Two-Color Agilent Whole Rat Genome Microarray, 4X44K in Genomic Core Facility of CABIMER (Design ID = 014879; Agilent Technologies, Palo Alto, CA, USA). The normalization of raw data and differential gene expression analysis were performed using the tools of Gene Expression Profile Analysis Suite (GEPAS) [12]. Genes with a fold-change greater than 1.5 and *p* > 0.05 (1363 genes) were considered as significant. Ingenuity pathway analysis software (IPA, Ingenuity Systems, Redwood City, CA, USA; www.ingenuity.com) was used for pathway and network analysis of differentially expressed genes.

### 2.7. Statistical Analysis

Data are presented as mean ± standard error of the mean (SEM). A one-way analysis of variance (ANOVA) test was applied when comparing three groups and Student’s *t*-test was used when comparing two groups. Homoscedasticity was checked by Barlett’s test, and non-parametric tests were applied when appropriate. Differences were considered significant at *p* < 0.05.

## 3. Results

The aim of this study has been to address if the digestion rate of carbohydrates contained within a high-fat diet during pregnancy could produce metabolic adaptations in the muscle of the offspring. Our studies were carried out at two time points to determine the effects of the maternal dietary intervention in the offspring, weaning and adolescence.

Firstly, body weight, lean body mass, muscle strength and muscle weight were measured in the different experimental groups. As we have previously published, maternal high-fat diets containing rapid-digesting carbohydrates (HF/RD) induced an increase in body weight in the adolescent offspring as compared with a similar group exposed to a gestational diet including slow-digesting carbohydrates (HF/SD). Furthermore, the adolescent rats from the HF/SD group showed an increase in lean body mass compared with the HF/RD rats [1].

**Figure 1:**
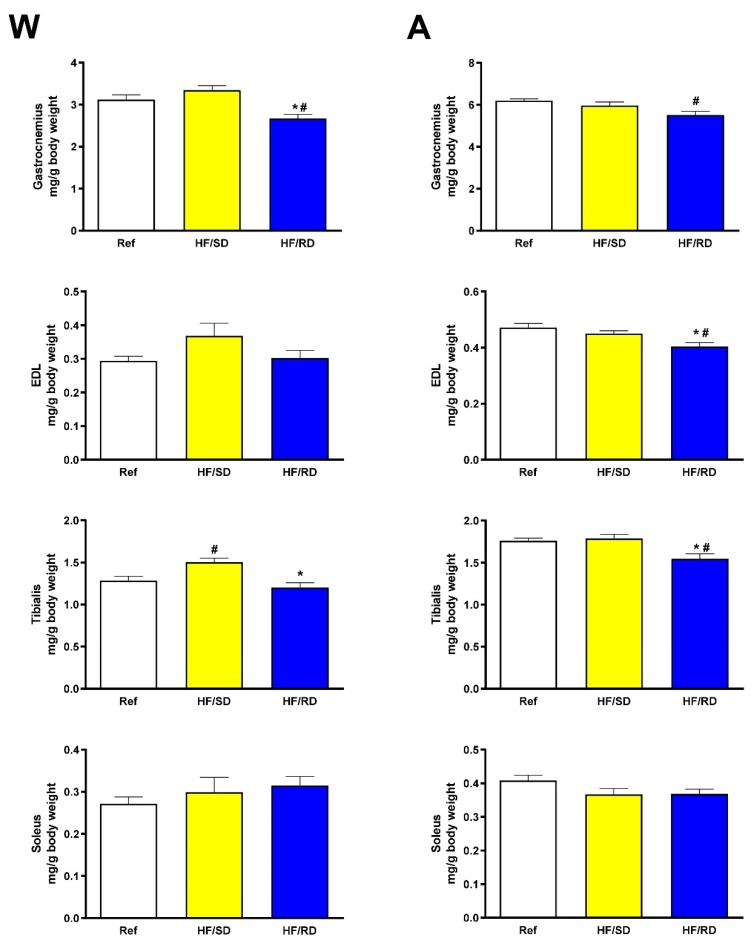
Muscle weight in the offspring. The muscle weight referenced to total animal weight were measured at weaning (**W**) and adolescence (**A**). Values are means ± standard error of the mean (SEM) (n = 8 for each experimental group). HF/SD: offspring from mothers on high-fat diet containing slow digesting carbohydrates; HF/RD: offspring from mothers on high-fat diet containing rapid digesting carbohydrates; Ref: offspring from mothers on AIN93G diet. * Significant difference with HF/SD group, *p* < 0.05. ^#^ Significant difference with reference group, *p* < 0.05. EDL: extensor digitorium longus.

When measured, the muscles’ weight in the HF/SD group increased significantly compared with the HF/RD group. Results are shown as muscle weight referenced to total animal weight (Figure 1) and as non-corrected muscle weight (Supplementary Figure S1). This increase was higher in muscles composed mainly of fast twitch fibers (gastrocnemius, EDL, tibialis) compared with muscles made mostly of slow twitch fibers as soleus [13], where significant differences were not detected.

**Figure 2:**
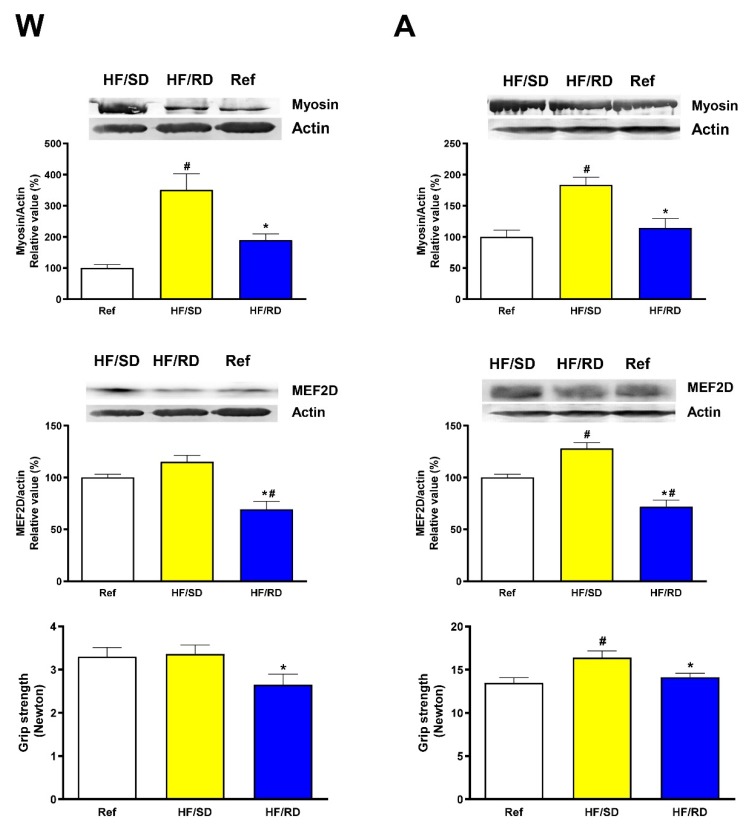
Muscle functionality in the offspring. Myosin content and grip strength were determined to address muscle functionality. MEF2D was measured as a marker of muscle differentiation at weaning (**W**) and adolescence (**A**). Values are means ± SEM (*n* = 8 for each experimental group). HF/SD: offspring from mothers on high-fat diet containing slow digesting carbohydrates; HF/RD: offspring from mothers on high-fat diet containing rapid digesting carbohydrates; Ref: offspring from mothers on AIN93G diet. * Significant difference with HF/SD group, *p* < 0.05. ^#^ Significant difference with reference group, *p* < 0.05.

Next, we assayed if these changes in muscle weight translated in an improvement of muscle functionality. For this purpose, myosin and myocyte-enhancer factor 2D (MEF2D) content in gastrocnemius and grip strength were measured (Figure 2).

Differences among groups were detected both at weaning and adolescence. The gastrocnemius myosin content was significant higher in the HF/SD group compared with the HF/RD and reference groups at weaning and at adolescence. Muscle differentiation is a coordinated process, where an array of transcription factors are differentially expressed over this process. One of the last transcription factors to be expressed as a marker of differentiation is the family of MEF2 transcriptional activators [14,15,16,17]. The expression of MEF2D (Figure 2) was significantly higher at weaning and adolescence in the HF/SD group compared with the HF/RD one (Figure 2).

The changes in myosin and MEF2D levels can be related with muscle functionality. At weaning and adolescence, forelimb grip strength in the HF/SD group was greater in comparison with the HF/RD group, which is in agreement with the significant increase found in fast twitch muscles’ weight. Furthermore, grip strength at adolescence was significantly higher in HF/SD rats when compared to the reference group.

Since there are positive effects of the slow digesting carbohydrates diet during pregnancy on muscle performance and differentiation of the offspring, the changes in key metabolic enzymes and transporters and signal transduction pathways were analyzed.

**Figure 3:**
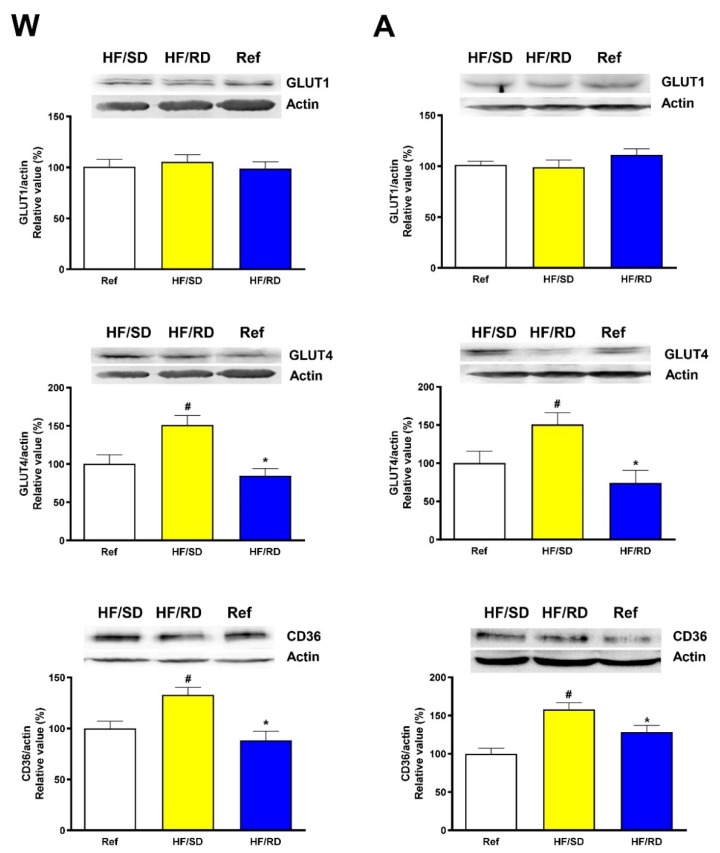
Fuel transporters expression in the offspring muscle. GLUT1, GLUT4 and FAT/CD36 transporters expression was assayed by Western blot in gastrocnemius muscle at weaning (**W**) and adolescence (**A**). Values are means ± SEM (*n* = 8 for each experimental group). HF/SD: offspring from mothers on high-fat diet containing slow digesting carbohydrates; HF/RD: offspring from mothers on high-fat diet containing rapid digesting carbohydrates; Ref: offspring from mothers on AIN93G diet. * Significant difference with HF/SD group, *p* < 0.05. ^#^ Significant difference with reference group, *p* < 0.05.

Muscle has the capability to select fuel use, thus being one of the main contributors to the body metabolic flexibility [4]. The two most relevant muscle fuels are glucose and fatty acids, which are selectively transported to the muscle in a highly regulated mode by glucose transporters (GLUT) and fatty acid transporter CD36 (FAT/CD36). Therefore, the expression of these transporters was assayed in the experimental groups (Figure 3).

Basal glucose transporter (GLUT1) and insulin-sensitive glucose transporter (GLUT4) were measured in the skeletal muscle samples from weaning and adolescent rats. At both ages, GLUT1 expression was similar among all experimental groups, as expected for a basal, non-regulated glucose transporter. On the contrary, rats from HF/RD group had significant lower level of GLUT4 compared to the HF/SD group both at weaning and adolescence. Furthermore, the HF/SD group showed significantly higher values compared with the reference control.

When the expression of FAT/CD36 transporter was measured, the results obtained mimicked those of the GLUT4 transporter. A significant increase in the expression of the transporter in the HF/SD group compared with the HF/RD group was detected, probably associated with a higher uptake of fatty acids.

**Figure 4:**
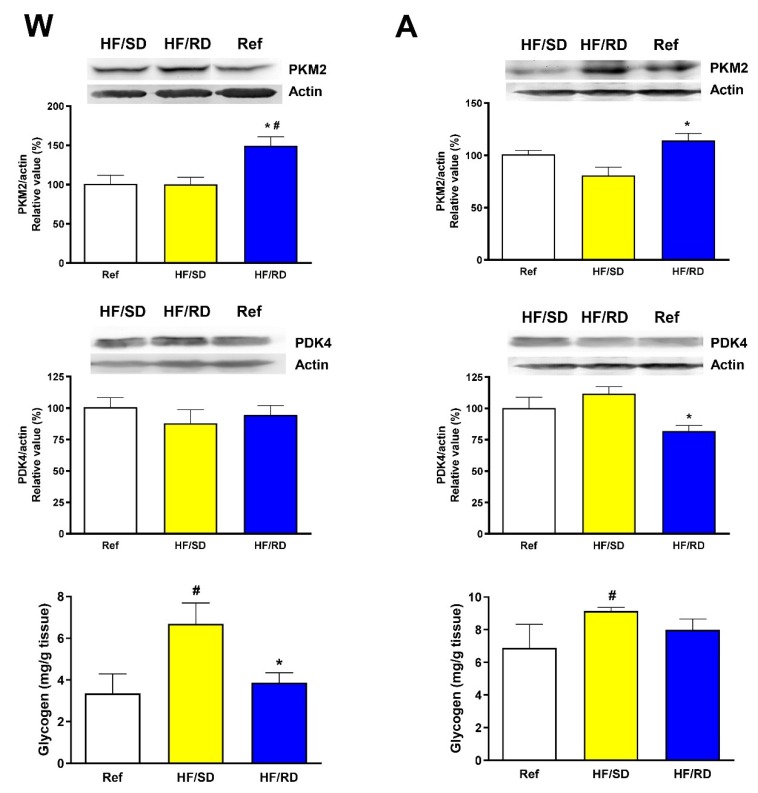
Muscle Pyruvate kinase isoenzyme M2 (PKM2) and Pyruvate Dehydrogenase Kinase 4 (PDK4) expression and glycogen content. PKM2 and PDK4 expression was assayed by Western blot in gastrocnemius muscle at weaning (**W**) and adolescence (**A**). Glycogen content was measured as described in the Methods Section. Values are means ± SEM (*n* = 8 for each experimental group). HF/SD: offspring from mothers on high-fat diet containing slow digesting carbohydrates; HF/RD: offspring from mothers on high-fat diet containing rapid digesting carbohydrates; Ref: offspring from mothers on AIN93G diet. * Significant difference with HF/SD group, *p* < 0.05. ^#^ Significant difference with reference group, *p* < 0.05.

The two main pathways involved in muscle catabolism to produce mechanical energy are glycolysis and lipolysis, and muscle metabolic flexibility is finely tuned by the muscle pyruvate kinase M2 isoenzyme (PKM2) and the pyruvate dehydrogenase kinase 4 (PDK4) [18,19]. When PKM2 expression was assayed (Figure 4), a significant difference was found between the HF/SD and HF/RD groups. The supplementation during pregnancy with slow digesting carbohydrates decreased and normalized PKM2 levels, suggesting a lower dependency of glucose to obtain energy in this experimental group. Additionally, PDK4 has a complex regulation that in the long term is controlled by its expression levels [18]. At adolescence, PDK4 expression was increased in the HF/SD group, therefore decreasing the use of the glycolytic pathway as the main energy source in this experimental group.

In addition, muscle glycogen content was determined (Figure 4). At weaning, the HF/SD group had significant higher glycogen content compared with the HF/RD and reference groups. This may indicate that the enhanced glucose uptake in the HF/SD group would be directed to glycogen synthesis rather than to the glycolytic pathway. At adolescence, the glycogen content of the HF/SD group remained significantly higher when compared to the reference group.

Mitochondrial enzymes involved in ATP synthesis were also assayed (Supplementary Figure S2). For this, ATPase subunit 5B (ATP5B) and the mitochondrial uncoupling protein 2 (UCP2) expression was assayed by Western blot. No significant differences were found among the different experimental groups, suggesting that there were no substantial alterations in the mitochondrial functionality.

**Figure 5:**
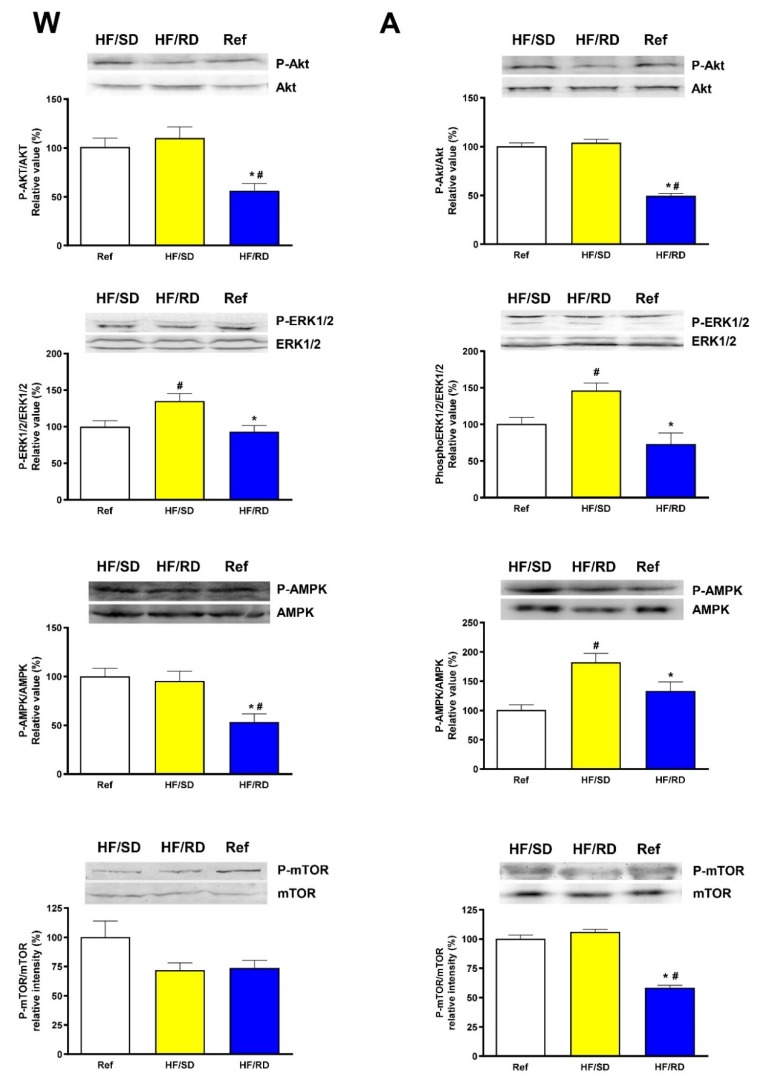
Modulation of muscle offspring signaling pathways by maternal diets. Phosphorylative status of Akt, ERK1/2, AMPK and mTOR was measured by Western blot in gastrocnemius muscle at weaning (**W**) and adolescence (**A**). Values are means ± SEM (*n* = 8 for each experimental group). HF/SD: offspring from mothers on high-fat diet containing slow digesting carbohydrates; HF/RD: offspring from mothers on high-fat diet containing rapid digesting carbohydrates; Ref: offspring from mothers on AIN93G diet. * Significant difference with HF/SD group, *p* < 0.05. ^#^ Significant difference with reference group, *p* < 0.05.

Therefore, up to this point, maternal nutrition shows a clear impact in the muscle of the offspring regarding muscle functionality and differentiation, and these changes are supported by metabolic adaptations that suggest an improved metabolic flexibility in the HF/SD group. We then proceed to identify which signaling pathways were modulating these changes in the offspring muscles. For that purpose, the phosphorylated status of key signaling protein kinases was assayed (Figure 5). The increase on insulin signaling pathway in the HF/SD group, measured as Akt phosphorylation (Figure 5A), was also associated with an enhanced signaling through the MAPK pathways measured by phosphorylation of ERK1/2 kinases.

Also, a kinase associated to the energy state of the muscle, AMP-activated protein kinase (AMPK), and a kinase associated to protein synthesis, the mechanistic target of rapamycin (mTOR), were measured. A protective effect of the HF/SD diet could be observed in both kinases when compared with the HF/RD group.

Since the maternal diet had been proven to influence muscle functionality, fuel use, metabolic flexibility and signaling pathways in the offspring, a strategy to discover if other cellular processes were also affected was set. For this, expression arrays of mRNA samples from HF/SD and HF/RD animals at adolescence were performed. A total of 1363 genes with fold-change higher that 1.5 and *p* < 0.05 were identified, and from those, a subgroup of 507 well-annotated genes were selected for further analysis (Supplementary materials Table S3).

**Figure 6:**
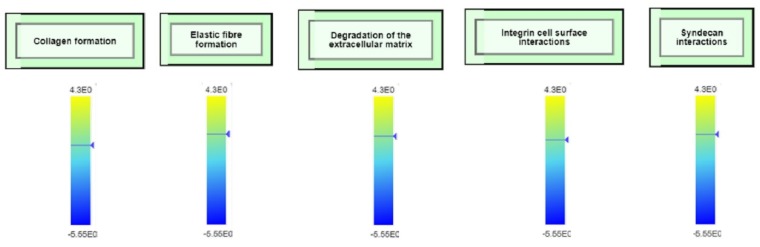
Enrichment of extracellular matrix (ECM)-related pathways in the HF/SD versus HF/RD group. KEGG analysis by STRING indicate an enrichment of pathways related with ECM. Entities are colored according to the expression scales when the HF/SD group is compared with the HF/RD group. Yellowish tones indicate positive fold-changes and bluish tones refer to negative fold-changes. The entities are partly colored as per the number of molecules of the pathway that were in the submitted list. The median for the pathway is flagged.

These genes were compared using protein-protein interaction networks functional enrichment analysis (STRING) (a database of known and predicted protein–protein interactions) and 499 nodes were identified, with an average local clustering coefficient of 0.299 and a (Protein-Protein Interaction) PPI enrichment *p*-value < 1 × 10^−16^. Of these, eight main clusters were identified with the highest confidence for interaction score (≥0.9) (Supplementary materials Table S4). Furthermore, STRING was also used for identifying the significantly enriched (Kyoto Encyclopedia of Genes and Genomes database) KEGG pathways (Supplementary materials Table S5). Of those enriched pathways, it is remarkable to indicate that the PI3K-Akt signaling pathway is one of those upregulated in the HF/SD group compared to the HF/RD one. When the data was analyzed using Reactome, a curated database of pathways and reactions in human biology, 936 pathways were hit by at least one modulated mRNA. Of these, most pathways were related to the extracellular matrix (ECM), in agreement with the KEGG analysis by STRING. Figure 6 shows the main pathways and the expression values for each one, indicating an enrichment in the HF/SD versus the HF/RD group.

**Figure 7:**
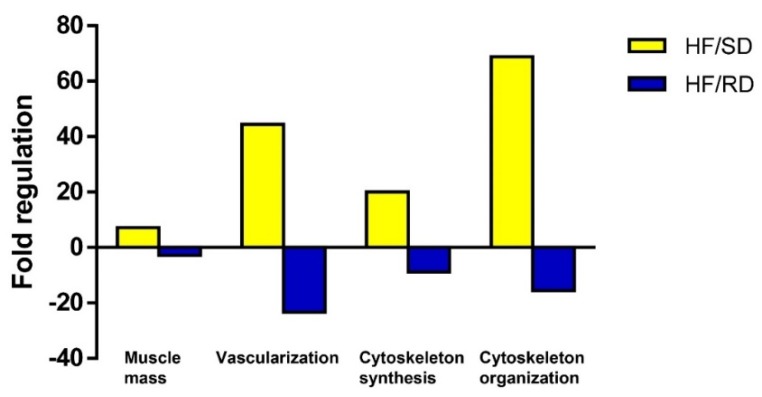
IPA ingenuity analysis of muscle-related processes. IPA analysis was used to predict whether these processes were increased or decreased as per the fold-change values of the genes involved and the influence of each individual gene on the biological function. Figure 7 shows how processes related to cytoskeleton and muscle mass are increased in HF/SD rats with respect to HF/RD ones.

To search for biological processes related to muscle function, a search in the Gene Ontology database, using PANTHER (Protein ANalysis THrough Evolutionary Relationships) and IPA ingenuity analysis were used to predict whether these processes were increased or decreased as per the fold-change values of the genes involved and the influence of each individual gene on the biological function. Results of the search are shown in Figure 7.

**Figure 8:**
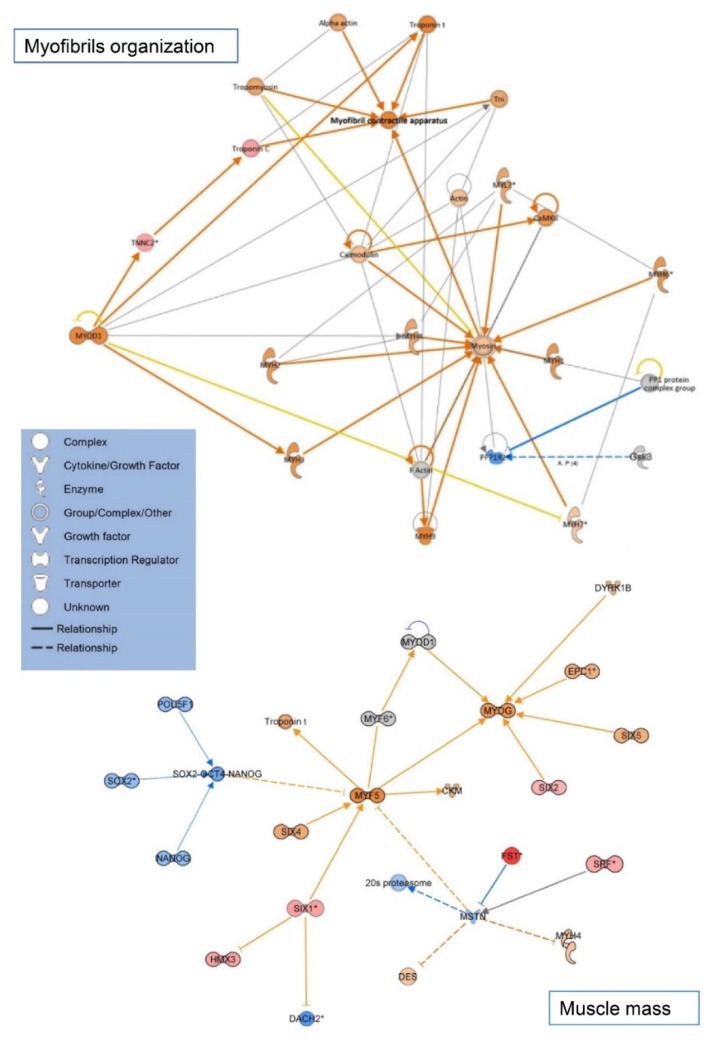
Biological interaction network of genes of the myofibrils’ organization and muscle mass pathways. Interaction networks were generated for the HF/SD versus HF/RD group comparison using IPA. Genes or gene products are represented as nodes, and the biological relationship between two nodes is represented as an edge. All edges are supported by at least one reference from the literature. Red and blue colored nodes indicate genes with up- and down-regulated expression respectively, in offspring from rats fed HF/SD diet compared with HF/RD diet. The intensity of the colors specifies the degree of up- or down-regulation. Greater intensity represents a higher expression level. Nodes and edges are displayed with various shapes and labels that present the functional class of genes and the nature of the relationships between the nodes.

Finally, a combined analysis using IPA allowed us to establish a network of genes that are modulated by the maternal diet and are involved in the control myofibrils organization and muscle mass (Figure 8). Again, expression data support the idea of up-regulation of both processes in the HF/SD group compared with the HF/RD one.

## 4. Discussions

Obesity, diabetes and metabolic syndrome in developing and developed countries are strongly associated to the intake of high-fat, high glycemic index (including rapid-digesting carbohydrates) diets. These diets affect the metabolism of key organs and tissues such as liver or adipose tissue, hampering whole body homeostasis. Their effects are even more negative during the early stages of development, such as pregnancy, impairing liver and adipose tissue metabolism in the offspring later.

Diets with a fat content of more than 40% energy based on animal fat lead to obesity, hyperglycemia and hypertriglyceridemia in rodents’ animal models with a similar profile to humans [20]. Glucose control during pregnancy in obese women is especially relevant for mothers and children. Replacement of simple sugars by low glycemic index carbohydrates has been proposed as useful [21]. These diets during pregnancy led to a decrease of gestational weight gain [22], and smaller fat deposits in the babies [23]. However, how this type of diet affects muscle metabolism has not been fully addressed to date.

Early programing of skeletal muscle metabolism is relevant from a double point of view. Firstly, muscle metabolic adaptations probably regulate its functionality, performance and strength. Secondly, muscle metabolism acts as a key regulator of body homeostasis, due to its metabolic flexibility, that is the capability to select fuel use and control body fuel channeling, hormone sensitivity and homeostasis. Since muscle has a lower priority in nutrient partition compared with liver during fetal development, muscle metabolic inflexibility is one of the first stages of peripheral insulin resistance and predisposition to obesity and type 2 diabetes later in life [6].

Previous reports from our group showed that a dietary intervention during pregnancy could have an important impact in the possibility for the offspring to develop some pathologies in later periods of life [1,2]. The consumption of HF diets that differ in the nature and digestion rate of carbohydrates by obese rats during pregnancy has allowed us to compare the long-term transgenerational effects of rapid-digesting carbohydrates, such as sucrose and maltodextrins, and slow-digesting carbohydrates, i.e., isomaltulose, resistant maltodextrins and fructooligosacharides. The use of slow digesting carbohydrates during pregnancy revealed to be of paramount importance, as it proved to prevent excess of adipogenesis in the offspring, modulating adipose tissue metabolism [1], to normalize liver metabolism and to reduce non-alcoholic fatty liver disease risk in the offspring [2].

The aim of this work has been to study the effects of the maternal diet during pregnancy on muscle metabolism, differentiation and performance in the offspring. In the same way as in the above-mentioned studies, HF/RD and HF/SD diets were selected, as reference AIN93G (up to weaning) and AIN93M (up to adolescence) diets were used. Since one of the main objectives was to determine the persistence over time of changes induced by the maternal feeding in the offspring, two time points were selected, weaning and late adolescence.

In general, muscles corresponding to the HF/SD group showed higher weight compared with the HF/RD group (Figure 1), this effect being more significant in muscles mainly composed of fast twitch fibers. Then, a grip strength assay was performed to determine if the changes in muscle weight translated in better muscle performance. The HF/SD group exhibited a better performance compared with the HF/RD group at both time points, and improved strength parameters compared to the reference group at adolescence (Figure 2). It has already been reported that adults on HF and high glycemic index diets showed poorer muscle performance [24], but we must remark that all the animals were on the same diet since birth while being tested and the only differential parameter was the maternal gestational diet. Therefore, it might conclude that the differences found were due to the early programming, showing that maternal nutrition has a resilient impact in the weight and muscle performance in the offspring. Furthermore, the slow digestive carbohydrate composition in the HF/SD diets apparently has some protective effects in the offspring, since this experimental group showed a significantly better muscle strength that the reference animals at adolescence.

The changes in grip strength are supported by a higher expression of myosin as a marker of muscle contraction and MEF2D, a late marker of muscle differentiation [15]. In the HF/SD group myosin and MEF2D levels were significantly higher than in HF/RD and reference groups. Muscle differentiation from myoblasts to myofibers is a complex process, where in a time-orchestrated mode, a panoply of transcription factors are expressed to promote differentiation [15,17]. One of the late differentiation markers is the MEF2 family. Four members, termed A–D, formed this family and some of them are essential for skeletal and cardiac muscle differentiation and performance [16]. In fact, an increase in MEF2A and MEF2D levels help to adapt skeletal muscle to exercise [25], while in diabetic animals, MEF2 levels are substantially reduced [26,27]. Furthermore, MEF2 binding sites appear in key muscle promoters such as creatine kinase [28] or myosin [17] that are directly involved in muscle performance. In addition, GLUT4 promoter expression is enhanced upon MEF2 binding [14,27]. Therefore, our results indicated that the HF/SD offspring showed a muscle programming that improved muscle strength and differentiation, and since it remains in place at adolescence, may point to a perinatal adaptation that could keep on during the life span.

The ability to select fuel for energy is one of the main features of the muscle. In fact, muscle selection of FA or glucose as the main energy sources is one of the bases of the metabolic flexibility concept [4]. Dietary carbohydrates are able to modify metabolic flexibility. A typical example is the capability of the muscle to switch between fat and glucose in response to an insulin stimulus. This flexibility takes place in healthy lean individuals and is hampered in diabetics and obese [29], reflecting a situation of metabolic inflexibility and metabolic syndrome.

Whether the muscle can use glucose or FA as fuels would be determinant in their metabolic adaptation to high or low glycemic index diets. In a healthy individual in a fasted, resting situation, muscle is mainly consuming FA from circulating triacylglycerols. FA are generated at the plasma membrane by a Lipoprotein lipase and internalized through an inducible FAT/CD36 transporter. Later on, FA will be activated as acyl-CoAs and then transported to the mitochondria where they are oxidized to obtain energy [19]. In a healthy individual, a high glycemic index diet elicits the translocation of GLUT4, a specific insulin-dependent glucose transporter, to the plasma membrane, while FA transport through the FAT/CD36 transporter is inhibited. This is a first insight of a metabolic fuel selection that will be further conditioned by a stimulation of the glycolytic pathway, an induction of the pyruvate kinase levels and even more important by an activation of the pyruvate dehydrogenase complex [18,30]. This metabolic switch works when the insulin signaling pathway is functional, while in obese or diabetic individuals, the amount of GLUT4 at the plasma membrane is reduced by at least 50% [31].

We have analyzed the expression of GLUT transporters in the different experimental groups. Since GLUT1 is a basal, insulin-independent glucose transporter, no significant changes were detected among the experimental groups at any time point, as shown in Figure 3. Conversely, a significant increase in the GLUT4 insulin-dependent transporter was detected in the HF/SD group compared to the HF/RD group at both weaning and adolescence. This result would suggest an increased insulin sensitivity in the HF/SD animals since insulin is the main regulator of GLUT4 expression and translocation. Moreover, GLUT4 promoter includes a MEF2 binding site, and it has been described that GLUT4 transcription is significantly upregulated through an increase in the production or activity of MEF2 transcription factors [14]. Therefore, the concomitant increase in MEF2 and GLUT4 levels observed in HF/SD rats, may reflect the importance of early programming in these animals. The increased expression of both proteins can be associated with an enhanced insulin sensitivity and the capability to prevent the impaired muscle glucose metabolism that is characteristic of diabetes mellitus and other metabolic syndromes. On the contrary, in agreement with the lower GLUT4 expression in the HF/RD group, it has been shown that a maternal cafeteria diet during pregnancy clearly decreased GLUT4 expression in the offspring muscles [32].

In parallel with the expression profile of GLUT4 transporter, FAT/CD36 transporter (Figure 3) is also significantly increased in the HF/SD offspring compared with the HF/RD group at weaning and adolescence. FAT/CD36 is responsible for the FA uptake as fuel in the muscle and its expression is hampered by high glycemic index diets [24]. Our results could indicate that the early programming that took place in the HF/RD offspring seemed to mimic this situation, where the capability of using FA as an energy source is limited, leading to muscle metabolic inflexibility and probably to lower muscle performance. On the contrary, the higher expression of both FAT/CD36 and GLUT4 transporters in the HF/SD offspring allow this group to be able to select fuels, from FA in repose to glucose upon insulin stimulation. This enhanced metabolic flexibility probably was partly responsible for the best muscle performance and grip strength in this experimental group.

In muscle, the fuel selection and metabolic flexibility also relays in the regulation of the pyruvate dehydrogenase complex (PDC) activity [33]. Activation of PDC takes place in muscles depending mainly on glucose as fuel and is indicative of metabolic syndrome. Mammalian PDC is inhibited by phosphorylation by pyruvate dehydrogenase kinase (PDK) isoenzymes, PDK4 being the main regulator of metabolic flexibility in skeletal muscle [18,30]. In addition to a short-term regulation by metabolite levels, PDK4 also shows a long-term regulation at the transcription level, its expression being diminished by high glycemic index diets [24]. It has been described that high-fat/low-carbohydrate diets reduce insulin-stimulated carbohydrate oxidation and promote glycogen storage. This effect is mediated by the muscle PDK4 [34].

The significant increase in PDK4 amount in the HF/SD compared with the HF/RD offspring (Figure 4) pointed to an adolescent HF/SD group mainly relying on FA for obtaining energy in basal conditions, and was further confirmed by the decreased expression of muscle pyruvate kinase (PKM2), one of the main regulators of the glycolytic pathway. At the same time, the expression of GLUT4 transporter was increased, probably due to an enhanced insulin sensitivity, the glucose being stored as glycogen in rapid twitching muscles (Figure 4). In fact, there was a significantly higher level of glycogen in the HF/SD group compared with the HF/RD group. Low muscle glycogen content has been described as an early marker of insulin resistance [35].

On the contrary, the higher levels of PKM2 in the HF/RD group compared with the HF/SD group could suggest that the muscle in HF/RD animals was probably depending on glucose to obtain energy and the glycolytic pathway and PDC were both active. In addition, since the expression of GLUT4 was reduced, the muscle functionality in this group could be compromised.

Our previous results [1,2] showed important differences in insulin sensitivity in both liver and adipose tissue. In muscle, the offspring of the HF/SD group showed an increase in the phosphorylation of Akt and ERK1/2 pathways. Both routes are involved in increasing glucose uptake by the muscle [14] as well as in improving insulin sensitivity. Therefore, even though high-fat diets are associated with insulin resistance, the supplementation of a diet with slow digesting carbohydrates had a beneficial effect by improving the insulin response in these muscles.

Obesity during pregnancy was associated with a decrease in AMPK-mediated signaling in the offspring leading to muscle insulin resistance [36]. An increase in phosphorylated AMPK was observed in the HF/SD group at both time points when compared with the HF/RD, supporting the idea that the HF/SD diet was able to prevent muscle insulin resistance.

Furthermore, it has been described [19] that phosphorylated AMPK was able to promote FA uptake by increasing CD36/FAT transporter as well as glucose uptake mediated by GLUT4. Our results in the HF/SD group agreed with this, since higher CD36/FAT and GLUT4 expression was observed, which could affect the use of muscle fuel.

mTOR is considered the kinase responsible to integrate signals that control growth and development [37,38]. Phosphorylated mTOR expression was higher in the HF/SD group than in the HF/RD group at adolescence. mTOR regulation is controlled by upstream signaling pathways, such as the insulin-dependent signaling measured by the phosphorylation status of Akt and ERK1/2, which are sensors of cell growth [39]. Therefore, it could be concluded that the higher phosphorylation of mTOR in HF/SD rats could be driven by the activation of the cited signaling pathways to synthesize more proteins and obtain better muscle performance.

Up to this point, our results indicated that the early programing provided by the HF/SD diet compared with the HF/RD diet had a strong impact on the regulation of signaling pathways and metabolic flexibility that lead to a better muscle performance. To investigate the extent of maternal diet effects, expression arrays were performed in those muscle samples obtained at adolescence from HF/SD and HF/RD groups. A total of 507 genes showed significant changes in their expression levels indicating that the programing provided by the maternal diet has a wide influence in the gene regulation of the offspring.

To further analyze the impact of these changes in gene expression, gene information was uploaded for pathway analysis and the information retrieved indicated that at least eight main clusters of genes were identified as targets for the early programing. These clusters included ribosomal proteins relevant for protein synthesis, proteins involved in regulation of actin polymerization, collagen formation, several binding proteins and proteins involved in RNA metabolism. In addition, when the information was uploaded in Reactome, ten modulated pathways were identified. Most of these pathways are related to the extracellular matrix (ECM). The modification in the gene networks indicated above translates the idea that not only key membrane transporters, regulatory enzymes and signaling pathways are programed, but also complementary pathways that taken together are responsible for the better performance in the muscles of the HF/SD group.

**Figure 9:**
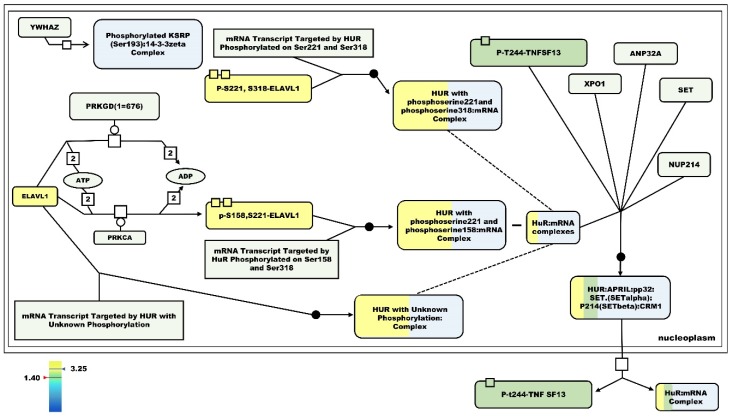
Elav1 pathway for mRNA stabilization. Entities are colored according to the expression scales when the HF/SD group is compared with the HF/RD group. The key elements in the pathway that are upregulated in HF/SL are shaded in yellowish-green tones, according to the expression scale. Yellowish tones indicate positive fold changes and bluish tones refer to negative fold-changes.

A pertinent question was how these changes are coordinated in the muscle cell. From the metabolic adaptations that we have described, an increase in insulin sensitivity appeared to be one of the main points of coordinated control. One of the top regulated genes found is the *Embryonic Lethal Abnormal Vision L1 (ElaV1)* gene, which increased its expression 3.2-fold in the HF/SD group compared to HF/RD. ElaV1/HuR protein belongs to the RNA binding proteins family [40]. Overexpression of this gene could explain the enhanced insulin sensitivity in the HF/SD offspring. In fact, as predicted by bioinformatics analysis, RNA metabolism was also upregulated in HF/SD when compared to HF/RD. Insulin-like binding proteins bind specific sets of RNA and regulate their translation, stability and subcellular localization, 38% of genes in the pathways were upregulated in the HF/SD group (5/13) (Figure 9).

Blockade of ElaV1/HuR protein by genetic manipulation in mice has been recently identified as responsible for metabolic inflexibility, mild obesity, impaired glucose tolerance, impaired fat oxidation and decreased in vitro palmitate oxidation [41]. All these data were similar to the results that we obtained by dietary early programming in the HF/RD group. Furthermore, all these alterations did not appear in the HF/SD group, in which the Elav1/HuR gene was strongly overexpressed.

A hypothesis termed fuel-mediated teratogenesis proposes that uterine exposure to an excess of fuels, mainly glucose, causes permanent fetal changes that lead to obesity in postnatal life [42]. The detailed mechanisms underlying how this nutritional challenge during pregnancy can induce lasting changes at the cellular level in the offspring are unknown. A proposed mechanism includes epigenetic modifications that affect the transcription of relevant genes and lead to changes in metabolism in target tissues such as hepatic and adipose tissue. In addition, changes in hormonal homeostasis associated with alterations in tissue sensitivity to these hormones have also been implicated in early programming. Additionally, a possible effect of the nutritional intervention during pregnancy is to alter the mammary gland development and therefore to influence lactation in the offspring, since obesity has been described as one of the main factors affecting the mammary gland development [43].

Our results extend the tissues targeted by early programming to the muscle. In humans, maternal obesity and gestational over-nutrition are harmful for the skeletal muscle development in the offspring, translating in reduced skeletal muscle area and number of fibers. These changes lead to impaired muscle functionality at adolescence [6]. In fact, our expression array data suggests that changes in the expression of crucial regulatory genes translate in changes in key metabolic enzymes associated with changes in muscle insulin sensitivity to promote muscle dysfunctionality. Apparently, the initial changes in the expression of regulator genes such as ElaV1/HuR can be avoided by supplementation during the pregnancy of slow digestive carbohydrates and consequently, can lead to the normalization of muscle metabolism, hormone response and functionality.

We consider that our results, albeit they are obtained in an experimental animal model, mimic the negative effects of the gestational over-nutrition in the HF/RD group. These negative effects were prevented when slow digesting carbohydrates were included in the diet of the HF/SD group. Therefore, a similar nutritional intervention in humans probably could have a strong impact in the muscle performance and metabolic flexibility of the offspring.

## 5. Conclusions

The supplementation of the diet during pregnancy with slow digesting carbohydrates prevented metabolic and signaling pathways that promote metabolic flexibility and appeared to exert a protective effect on insulin resistance in the offspring. These effects were produced at several levels, from gene expression and signal transduction sensitizing to fuel transporters and key metabolic enzymes’ modulation, leading to a better muscle functionality. Data from gene expression indicated the relevant role of some genes, such as ElaV1/HuR, in the coordinated regulation of this programming. Our results highlighted the relevance of dietary carbohydrates during pregnancy to manage muscle metabolism in the offspring. In addition, as we previously reported, the uptake of slow digesting carbohydrates diets during pregnancy also promoted beneficial effects on liver and adipose tissue metabolism in the offspring, indicating synergic and crosstalk effects in different tissues to orchestrate whole body homeostasis.

## Supplementary Materials

The following are available online at https://www.mdpi.com/2072-6643/12/2/528/s1, Figure S1; Non- corrected muscle weight in the offspring. Figure S2: Muscle ATPase subunit 5B (ATP5B) and the mitochondrial uncoupling protein 2 (UCP2) expression; Table S1: Composition of experimental diets. Table S2: Biochemical Serum Parameters. Table S3: Expression arrays of mRNA samples from HF/SD and HF/RD animals at adolescence; Table S4: STRING (a database of known and predicted protein-protein interactions) analysis of differentially expressed genes from HF/SD and HF/RD animals at adolescence; Table S5: Top 10 KEGG pathways identified by STRING from the analysis of differentially expressed genes from HF/SD and HF/RD animals at adolescence.
