# Supplementary material for: Programming Skeletal Muscle Metabolic Flexibility in Offspring of Male Rats in Response to Maternal Consumption of Slow Digesting Carbohydrates during Pregnancy
Source: Nutrients. 2020 Feb 19;12(2):528. doi: 10.3390/nu12020528 (PMC7071425; doi:10.3390/nu12020528)
Supplement: Supplementary file 1 [file nutrients-12-00528-s001.pdf]

# Programming skeletal muscle metabolic flexibility in offspring of male rats in response to maternal consumption of slow digesting carbohydrates during pregnancy

**Rafael Salto[1#*], María D Girón[1#], Manuel Manzano[2], María J Martín[2], Jose D Vílchez[1], Pilar Bueno-Vargas[2], Elena Cabrera[1], Mónica Pérez-Alegre[3], Eloisa Andujar[3], Ricardo Rueda[2], Jose M Lopez-Pedrosa[2]**

[1] Department of Biochemistry and Molecular Biology II, School of Pharmacy, University of Granada, Campus de Cartuja, 18071 Granada, Spain; rsalto@ugr.es (RS), mgiron@ugr.es (MDG), e.damaso@go.ugr.es (JDV), elenacc_20@hotmail.com (EC)

[2] Abbott Nutrition R&D, Abbott Laboratories, 18004 Granada, Spain; manuel.manzano@abbott.com (MM), chmmmj@yahoo.com (MJM), pilar.bueno@abbott.com (PB-V), ricardo.rueda@abbott.com (RR), jose.m.lopez@abbott.com (JMLP)

[3] Centro Andaluz de Biología Molecular y Medicina Regenerativa-CABIMER, Universidad de Sevilla-CSIC-Universidad Pablo de Olavide, Seville, Spain; monica.perez@cabimer.es (MP-A), eloisa.andujar@cabimer.es (EA)

# These authors contributed equally to this work.

* Correspondence: rsalto@ugr.es; Tel.: +34-958-246363 (RS)

**SUPPLEMENTARY MATERIAL**

- Supplementary Figure S1
- Supplementary Figure S2
- Supplementary Table S1
- Supplementary Table S2
- Supplementary Table S3
- Supplementary Table S4
- Supplementary Table S5

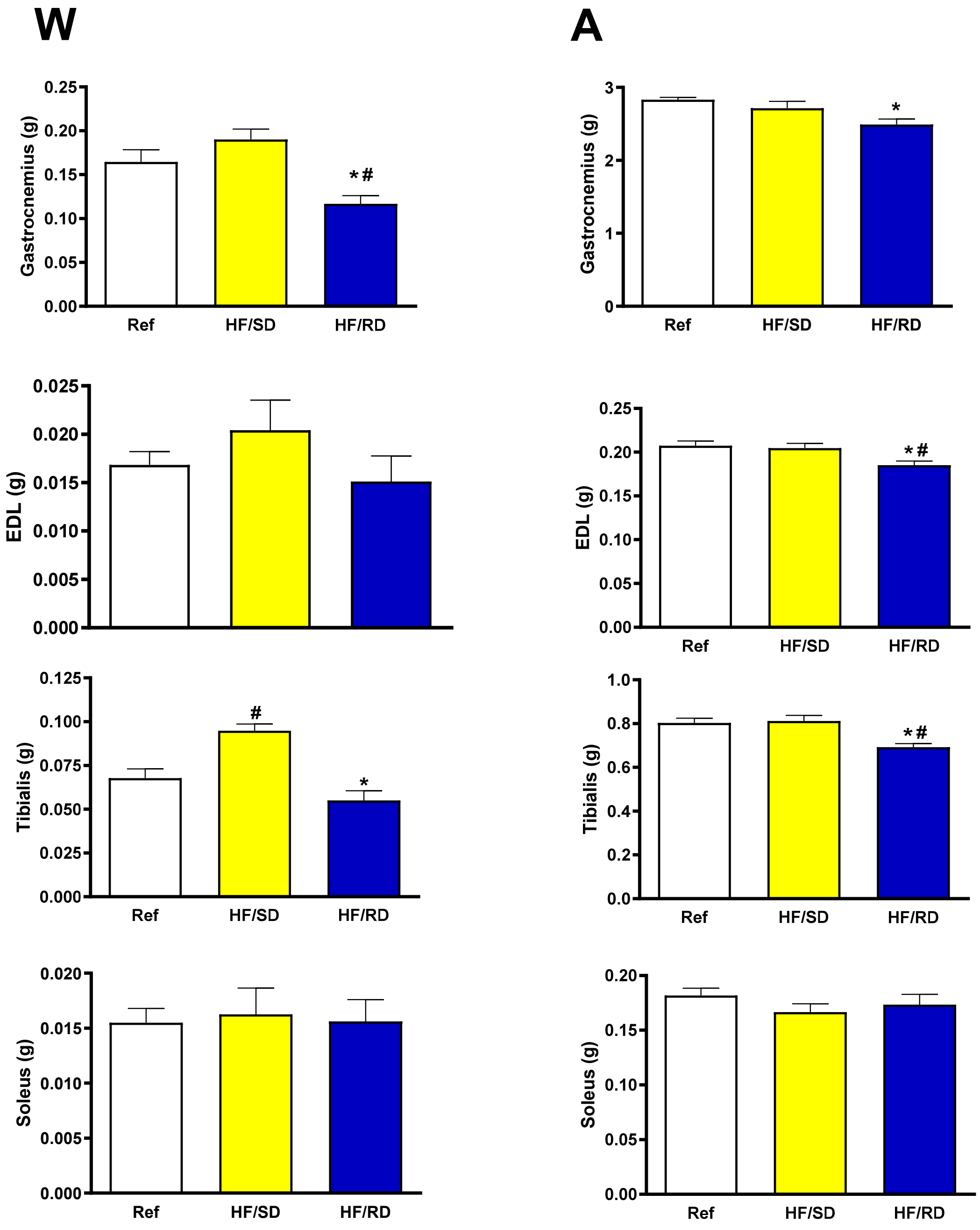


*Figure S1.* Muscle weight in the offspring. The muscle weight has been measured at weaning (W) and adolescence (A). Values are means ± SEM (n=8 for each experimental group). HF/SD: offspring from mothers on high fat diet containing slow digesting carbohydrates; HF/RD: offspring from mothers on high fat diet containing rapid digesting carbohydrates; Ref: offspring from mothers on AIN93G diet. * Significant difference with HF/SD group, $p<0.05$. # Significant difference with reference group, $p<0.05$. EDL: extensor digitorium longus.

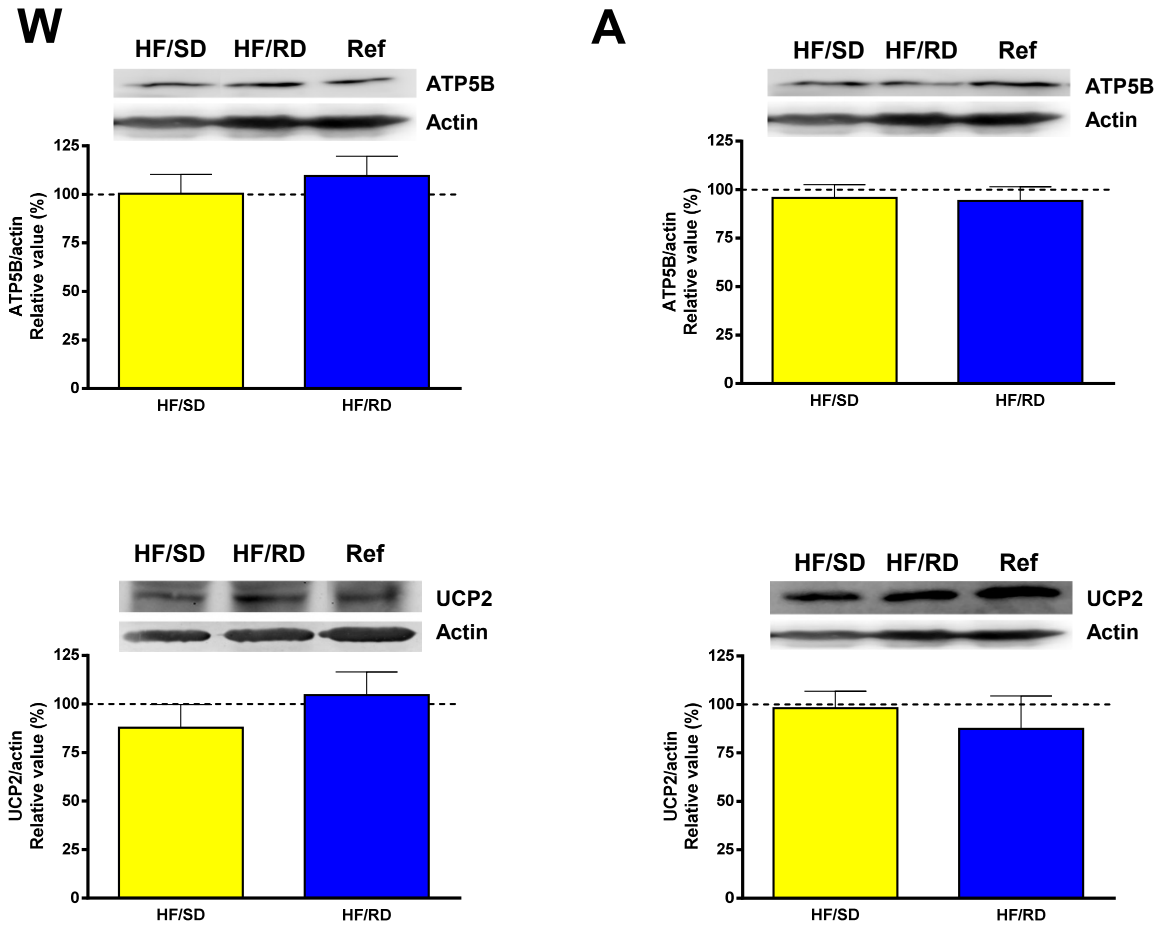


**Supplementary Figure S2.** *Muscle ATPase subunit 5B (ATP5B) and the mitochondrial uncoupling protein 2 (UCP2) expression.* Expression was assayed by Western blot in gastrocnemius muscle at weaning (W) and adolescence (A). Values are means ± SEM (n=8 for each experimental group). HF/SD: offspring from mothers on high fat diet containing slow digesting carbohydrates; HF/RD: offspring from mothers on high fat diet containing rapid digesting carbohydrates; Ref: offspring from mothers on AIN93G diet. Line in all graphs shows reference group mean value. No significant differences were found.

**Supplementary Materials Table S1. Composition of experimental diets**

| | Reference diets | | Obesogenic diet | Experimental diets | |
|---|---|---|---|---|---|
| | AIN93G | AIN93M | HF | HF/RD | HF/SD |
| **Total Protein** [a] (g/100g diet) | 18.3 | 12.6 | 24.2 | 24.2 | 24.2 |
| Calcium caseinate (%) | 100 | 100 | 100 | 100 | 100 |
| **Total fat** [b] (g/100g diet) | 7.0 | 4.0 | 20.5 | 20.5 | 20.5 |
| Plant oil (%) | 100 | 100 | | | |
| Lard fat (%) | | | 100 | 100 | 100 |
| **Total CHO** (g/100g diet) | 64.6 | 75.7 | 49.4 | 49.4 | 49.1 |
| Sugar [c] | | | | | |
| Isomaltulose (%) | - | - | - | - | 67.9 |
| Sucrose (%) | 14.4 | 13.5 | 56.0 | 81.9 | 5.5 |
| Rapid-digesting complex CHO [c] | | | | | |
| Maltodextrin/Corn starch (%) | 78.4 | 79.7 | - | - | 7.7 |
| Resistant maltodextrin (%) | - | - | 7.9 | - | 15.4 |
| Indigestible fiber [c] | | | | | |
| Fructooligosaccharides (%) | - | - | - | 18.1 | 3.5 |
| Cellulose (%) | 7.2 | 6.8 | - | - | - |

Individual (a) protein, (b) fat and (c) CHO composition is expressed as percentage of total protein, fat and CHO, respectively

**Supplementary Table S2. Biochemical Serum Parameters.**

**Weanning**

| | HF/SD Group | HF/RD Group | Reference |
|---|---|---|---|
| Glycaemia (mg/dL) | 169.4 ± 4.7 | 163.1 ± 7.7 | 170.6 ± 4.2 |
| TG (mg/dL) | 49.1 ± 4.5 | 85.8 ± 14.8* | 55.6 ± 7.7 |

**Adolescence**

| | HF/SD Group | HF/RD Group | Reference |
|---|---|---|---|
| Glycaemia (mg/dL) | 104.6 ± 5.0 | 119.0 ± 6.4 | 117.3 ± 12.8 |
| TG (mg/dL) | 62.3 ± 5.2 | 123.2 ± 30.5 | 59.4 ± 5.9 |

(n=8) * Significant difference with HF/SD group.

**Homeostasis model assessment (HOMA) at Adolescence**

| | HF/SD Group | HF/RD Group | Reference |
|---|---|---|---|
| AUC glycaemia (mg·min/dL) | 25415 ± 857.8 | 28112 ± 1351 | 27391 ± 524.3 |
| AUC insulinemia (µg·min/L) | 341066 ± 25027 | 393824 ± 53570 | 359118 ± 30052 |
| HOMA (index) | 5.72 ± 1.093 | 9.83 ± 2.425 | 9.875 ± 2.748 |

We have measured in the offspring of the different experimental groups a homeostasis model assessment (HOMA) as an index of insulin resistance, a meal tolerance test and during the test the plasma glucose and insulin levels have been quantitated (Martin, *et al. The Journal of nutritional biochemistry* **2018,** 61, 183-196). The results expressed as Area under the Curve (AUC) for the different groups (n=8). Prosprandial glycemic peak and total glycemic response were significantly lower in the HF/SD group compared with HF/RD group.

**Supplementary Material Table S3.** *Expression arrays of mRNA samples from HF/SD and HF/RD animals at adolescence.* A total of 1363 genes with higher that 1.5 fold change and $p < 0.05$ were detected and from those, a subgroup of 507 well annotated genes were selected.

| M (log2 fold change) | Linear Fold Change | Increase/ Decrease | Statistic | p-value | Adjusted p-value | GeneName | Description | Genbank Accession |
|---|---|---|---|---|---|---|---|---|
| **2.88** | 7.34 | Increase | 7.38 | 4.6793E-07 | 0.0121 | similar to alkaline phosphatase (EC 3.1.3.1) precursor, placental-like - human (predicted) | Rattus norvegicus similar to alkaline phosphatase precursor (LOC367308), mRNA [XM_346070] | XM_346070 |
| **2.11** | 4.31 | Increase | 7.25 | 6.0042E-07 | 0.0121 | ubiquitously transcribed tetratricopeptide repeat gene, X chromosome (predicted) | PREDICTED: Rattus norvegicus ubiquitously transcribed tetratricopeptide repeat gene, X chromosome (predicted) (Utx_predicted), mRNA [XM_001055391] | XM_001055391 |
| **1.16** | 2.23 | Increase | 7.06 | 8.8496E-07 | 0.0121 | AI237189 | AI237189 EST233751 Normalized rat ovary, Bento Soares Rattus sp. cDNA clone ROVDP71 3' end, mRNA sequence [AI237189] | AI237189 |
| **1.22** | 2.33 | Increase | 6.52 | 2.6988E-06 | 0.0184 | AA859053 | AA859053 UI-R-A0-ak-e-04-0-UI.s1 UI-R-A0 Rattus norvegicus cDNA clone UI-R-A0-ak-e-04-0-UI 3', mRNA sequence [AA859053] | AA859053 |
| **2.01** | 4.04 | Increase | 6.51 | 2.758E-06 | 0.0184 | similar to Protein C6orf78 homolog | PREDICTED: Rattus norvegicus similar to Protein C6orf78 homolog (LOC499445), mRNA [XM_574768] | XM_574768 |
| **-1.43** | 2.70 | Decrease | -6.22 | 5.0911E-06 | 0.0184 | RGD1308297 | Rattus norvegicus similar to CG10084-PA (LOC306137), mRNA [XM_224510] | XM_224510 |
| **-1.22** | 2.32 | Decrease | -6.03 | 7.527E-06 | 0.0184 | BF556301 | BF556301 UI-R-A1-en-d-10-0-UI.r1 UI-R-A1 Rattus norvegicus cDNA | BF556301 |

| | | | | | | | | |
|---|---|---|---|---|---|---|---|---|
| | | | | | | | clone UI-R-A1-en-d-10-0-UI 5', mRNA sequence [BF556301] | |
| **-1.02** | 2.03 | Decrease | -5.91 | 9.9477E-06 | 0.0207 | TC552847 | APC_RAT (P70478) Adenomatous polyposis coli protein (APC protein), complete [TC552847] | |
| **1.78** | 3.44 | Increase | 5.88 | 1.0397E-05 | 0.0207 | hypothetical protein LOC685924 | PREDICTED: Rattus norvegicus hypothetical protein LOC685924 (LOC685924), mRNA [XM_001065777] | XM_001065777 |
| **1.04** | 2.05 | Increase | 5.84 | 1.1437E-05 | 0.0207 | BE106074 | BE106074 UI-R-BO1-ask-f-04-0-UI.s1 UI-R-BO1 Rattus norvegicus cDNA clone UI-R-BO1-ask-f-04-0-UI 3', mRNA sequence [BE106074] | BE106074 |
| **-1.21** | 2.32 | Decrease | -5.78 | 1.3075E-05 | 0.0207 | AA998118 | AA998118 UI-R-C0-hx-c-06-0-UI.s1 UI-R-C0 Rattus norvegicus cDNA clone UI-R-C0-hx-c-06-0-UI 3', mRNA sequence [AA998118] | AA998118 |
| **1.19** | 2.29 | Increase | 5.73 | 1.4431E-05 | 0.0207 | similar to Der1-like domain family, member 1 | PREDICTED: Rattus norvegicus similar to Der1-like domain family, member 1 (LOC689741), mRNA [XM_001071836] | XM_001071836 |
| **2.30** | 4.93 | Increase | 5.73 | 1.4671E-05 | 0.0207 | tryptase alpha/beta 1 | Rattus norvegicus tryptase alpha/beta 1 (Tpsab1), mRNA [NM_019322] | NM_019322 |
| **1.83** | 3.56 | Increase | 5.72 | 1.4887E-05 | 0.0207 | SH2 domain containing phosphatase anchor protein 1 (predicted) | PREDICTED: Rattus norvegicus SH2 domain containing phosphatase anchor protein 1 (predicted) (Spap1_predicted), mRNA [XM_227483] | XM_227483 |
| **2.06** | 4.18 | Increase | 5.55 | 2.182E-05 | 0.0263 | olfactory receptor 567 (predicted) | Rattus norvegicus olfactory receptor 567 (predicted) (Olr567_predicted), mRNA [NM_001000326] | NM_001000326 |
| **-1.82** | 3.54 | Decrease | -5.52 | 2.3065E-05 | 0.0263 | BF523141 | BF523141 UI-R-C3-sl-b-09-0-UI.r1 UI-R-C3 Rattus norvegicus cDNA clone | BF523141 |

| | | | | | | | | |
|---|---|---|---|---|---|---|---|---|
| | | | | | | | UI-R-C3-sl-b-09-0-UI 5', mRNA sequence [BF523141] | |
| **1.63** | 3.09 | Increase | 5.42 | 2.8987E-05 | 0.0290 | AI171355 | AI171355 EST217310 Normalized rat muscle, Bento Soares Rattus sp. cDNA clone RMUBJ63 3' end, mRNA sequence [AI171355] | AI171355 |
| **1.46** | 2.75 | Increase | 5.39 | 3.0771E-05 | 0.0300 | cyclin-dependent kinase inhibitor 1C (P57) | Rattus norvegicus cyclin-dependent kinase inhibitor 1C (P57) (Cdkn1c), transcript variant 3, mRNA [NM_182735] | NM_182735 |
| **-1.07** | 2.10 | Decrease | -5.36 | 3.274E-05 | 0.0312 | AW144111 | AW144111 EST294407 Normalized rat embryo, Bento Soares Rattus sp. cDNA clone RGICI34 5' end, mRNA sequence [AW144111] | AW144111 |
| **-1.22** | 2.32 | Decrease | -5.31 | 3.6921E-05 | 0.0329 | AW916157 | AW916157 EST347461 Rat gene index, normalized rat, norvegicus, Bento Soares Rattus norvegicus cDNA clone RGIDI75 5' end, mRNA sequence [AW916157] | AW916157 |
| **-1.02** | 2.02 | Decrease | -5.27 | 4.0501E-05 | 0.0339 | AI102821 | AI102821 EST212110 Normalized rat embryo, Bento Soares Rattus sp. cDNA clone REMBT30 3' end, mRNA sequence [AI102821] | AI102821 |
| **-1.12** | 2.17 | Decrease | -5.24 | 4.289E-05 | 0.0339 | TC520328 | Unknown | |
| **1.96** | 3.89 | Increase | 5.20 | 4.7236E-05 | 0.0339 | calcium/calmodulin-dependent protein kinase I | Rattus norvegicus calcium/calmodulin-dependent protein kinase I (Camk1), mRNA [NM_134468] | NM_134468 |
| **1.69** | 3.22 | Increase | 5.19 | 4.8002E-05 | 0.0339 | sialic acid acetylesterase (predicted) | PREDICTED: Rattus norvegicus sialic acid acetylesterase (predicted) (Siae_predicted), mRNA [XM_343373] | XM_343373 |

| | | | | | | | | |
|---|---|---|---|---|---|---|---|---|
| **1.93** | 3.82 | Increase | 5.11 | 5.7646E-05 | 0.0347 | matrix metallopeptidase 2 | Rattus norvegicus matrix metallopeptidase 2 (Mmp2), mRNA [NM_031054] | NM_031054 |
| **1.49** | 2.82 | Increase | 5.10 | 5.86E-05 | 0.0347 | G-protein signalling modulator 1 (AGS3-like, C. elegans) | Rattus norvegicus G-protein signalling modulator 1 (AGS3-like, C. elegans) (Gpsm1), mRNA [NM_144745] | NM_144745 |
| **1.24** | 2.36 | Increase | 5.09 | 6.0282E-05 | 0.0347 | protein phosphatase 4, catalytic subunit | Rattus norvegicus protein phosphatase 4, catalytic subunit (Ppp4c), mRNA [NM_134359] | NM_134359 |
| **1.27** | 2.41 | Increase | 5.04 | 6.7496E-05 | 0.0360 | CXXC finger 5 | PREDICTED: Rattus norvegicus similar to CXXC finger 5 (LOC681300), mRNA [XM_001061130] | XM_001061130 |
| **-1.45** | 2.73 | Decrease | -5.04 | 6.8405E-05 | 0.0360 | centrosome-associated protein 350 | PREDICTED: Rattus norvegicus centrosome-associated protein 350 (Cap350), mRNA [XM_001067472] | XM_001067472 |
| **2.23** | 4.69 | Increase | 5.00 | 7.3874E-05 | 0.0379 | XM_216797 | Rattus norvegicus hypothetical LOC299350 (LOC299350), mRNA [XM_216797] | XM_216797 |
| **1.56** | 2.95 | Increase | 4.96 | 8.0953E-05 | 0.0394 | Hist1h2an_predicted | PREDICTED: Rattus norvegicus histone 1, H2an (predicted) (Hist1h2an_predicted), mRNA [XM_225386] | XM_225386 |
| **1.48** | 2.80 | Increase | 4.96 | 8.1351E-05 | 0.0394 | AF010441 | Rattus norvegicus MARRLC6A mRNA, partial cds. [AF010441] | AF010441 |
| **-1.77** | 3.42 | Decrease | -4.84 | 0.00010713 | 0.0448 | DY472414 | RVL23737 Wackym-Soares normalized rat vestibular cDNA library Rattus norvegicus cDNA 5', mRNA sequence [DY472414] | DY472414 |
| **1.06** | 2.09 | Increase | 4.81 | 0.00011444 | 0.0454 | zinc finger, DHHC domain containing 18 | Rattus norvegicus zinc finger, DHHC domain containing 18 (Zdhhc18), mRNA [NM_001039339] | NM_001039339 |

| | | | | | | | | |
|---|---|---|---|---|---|---|---|---|
| **2.76** | 6.77 | Increase | 4.79 | 0.00012011 | 0.0456 | FBJ osteosarcoma oncogene B | PREDICTED: Rattus norvegicus FBJ osteosarcoma oncogene B (Fosb), mRNA [XM_001057199] | XM_001057199 |
| **-2.35** | 5.09 | Decrease | -4.77 | 0.00012372 | 0.0464 | mutL homolog 1 (E. coli) | Rattus norvegicus mutL homolog 1 (E. coli) (Mlh1), mRNA [NM_031053] | NM_031053 |
| **2.29** | 4.91 | Increase | 4.76 | 0.00012677 | 0.0464 | actin, gamma 2 | Rattus norvegicus actin, gamma 2 (Actg2), mRNA [NM_012893] | NM_012893 |
| **1.57** | 2.97 | Increase | 4.73 | 0.00013583 | 0.0470 | XM_345216 | Rattus norvegicus similar to Ab2-162 (LOC365803), mRNA [XM_345216] | XM_345216 |
| **4.30** | 19.69 | Increase | 4.73 | 0.00013646 | 0.0470 | Myh11 | PREDICTED: Rattus norvegicus myosin, heavy polypeptide 11, smooth muscle, transcript variant 1 (Myh11), mRNA [XM_001053321] | XM_001053321 |
| **1.23** | 2.35 | Increase | 4.72 | 0.00014086 | 0.0470 | similar to ribosomal protein L13 | PREDICTED: Rattus norvegicus similar to ribosomal protein L13 (LOC680938), mRNA [XM_001059551] | XM_001059551 |
| **-1.08** | 2.11 | Decrease | -4.71 | 0.00014341 | 0.0470 | BF550737 | BF550737 UI-R-C0-jk-c-04-0-UI.r1 UI-R-C0 Rattus norvegicus cDNA clone UI-R-C0-jk-c-04-0-UI 5', mRNA sequence [BF550737] | BF550737 |
| **-1.67** | 3.18 | Decrease | -4.70 | 0.00014738 | 0.0470 | TC548806 | S79979 ribosomal protein L37 {Homo sapiens;} , partial (85%) [TC548806] | |
| **1.53** | 2.89 | Increase | 4.68 | 0.00015479 | 0.0478 | Hermansky-Pudlak syndrome 1 homolog (human) | Rattus norvegicus Hermansky-Pudlak syndrome 1 homolog (human) (Hps1), mRNA [NM_040669] | NM_040669 |
| **-1.48** | 2.79 | Decrease | -4.64 | 0.00016679 | 0.0496 | ATP-binding cassette, sub-family A (ABC1), member 7 | Rattus norvegicus ATP-binding cassette, sub-family A (ABC1), member 7 (Abca7), mRNA [NM_207598] | NM_207598 |
| **-2.11** | 4.32 | Decrease | -4.62 | 0.00017752 | 0.0509 | Sh3bp1 | PREDICTED: Rattus norvegicus reg I binding protein I (Rbp1), mRNA [XM_235500] | XM_235500 |

| | | | | | | | | |
|---|---|---|---|---|---|---|---|---|
| **1.47** | 2.77 | Increase | 4.61 | 0.00018103 | 0.0516 | AI233279 | AI233279 EST229967 Normalized rat kidney, Bento Soares Rattus sp. cDNA clone RKIDD12 3' end, mRNA sequence [AI233279] | AI233279 |
| **3.05** | 8.31 | Increase | 4.60 | 0.00018317 | 0.0518 | claudin 23 | Rattus norvegicus claudin 23 (Cldn23), mRNA [NM_001033062] | NM_001033062 |
| **1.36** | 2.57 | Increase | 4.59 | 0.00019031 | 0.0531 | similar to Williams-Beuren syndrome critical region protein 28 isoform A (predicted) | PREDICTED: Rattus norvegicus similar to Williams-Beuren syndrome critical region protein 28 isoform A (predicted) (RGD1561903_predicted), mRNA [XM_213758] | XM_213758 |
| **1.56** | 2.95 | Increase | 4.58 | 0.00019306 | 0.0531 | XM_214608 | Rattus norvegicus similar to FH1/FH2 domains-containing protein (Formin homolog overexpressed in spleen) (FHOS) (Formin homology 2 domain containing 1) (LOC291731), mRNA [XM_214608] | XM_214608 |
| **-1.06** | 2.09 | Decrease | -4.52 | 0.00022176 | 0.0576 | TC563522 | Q6XKT3 (Q6XKT3) Long-wavelength rhodopsin (Fragment), partial (6%) [TC563522] | |
| **2.00** | 3.99 | Increase | 4.49 | 0.00023953 | 0.0585 | similar to histone 2a | PREDICTED: Rattus norvegicus similar to histone 2a (LOC682543), mRNA [XM_001061959] | XM_001061959 |
| **2.49** | 5.60 | Increase | 4.48 | 0.0002458 | 0.0590 | guanylate cyclase 2g | Rattus norvegicus guanylate cyclase 2g (Gucy2g), mRNA [NM_139042] | NM_139042 |
| **1.51** | 2.85 | Increase | 4.47 | 0.00024737 | 0.0590 | PEF protein with a long N-terminal hydrophobic domain | Rattus norvegicus PEF protein with a long N-terminal hydrophobic domain (Peflin), mRNA [NM_001007651] | NM_001007651 |
| **1.00** | 2.00 | Increase | 4.47 | 0.00025114 | 0.0595 | cleavage and polyadenylation specific factor 5 | Rattus norvegicus cleavage and polyadenylation specific factor 5 (Cpsf5), mRNA [NM_001039004] | NM_001039004 |
| **-1.11** | 2.16 | Decrease | -4.45 | 0.00026288 | 0.0608 | Smarca4 | PREDICTED: Rattus norvegicus SWI/SNF related, matrix associated, | XM_343358 |

| | | | | | | | | |
|---|---|---|---|---|---|---|---|---|
| | | | | | | | actin dependent regulator of chromatin, subfamily a, member 4 (Smarca4), mRNA [XM_343358] | |
| **-1.46** | 2.75 | Decrease | -4.43 | 0.00027021 | 0.0618 | similar to MYST histone acetyltransferase monocytic leukemia 4 (predicted) | PREDICTED: Rattus norvegicus similar to MYST histone acetyltransferase monocytic leukemia 4 (predicted) (RGD1566399_predicted), mRNA [XR_008228] | XR_008228 |
| **1.45** | 2.72 | Increase | 4.43 | 0.0002722 | 0.0618 | similar to mast cell protease 1-like 3 precursor (predicted) | PREDICTED: Rattus norvegicus similar to mast cell protease 1-like 3 precursor (predicted) (RGD1562035_predicted), mRNA [XM_224209] | XM_224209 |
| **-2.17** | 4.51 | Decrease | -4.41 | 0.00028266 | 0.0627 | TC549700 | Q9ERK2 (Q9ERK2) Neprilysin-like peptidase gamma, partial (5%) [TC549700] | |
| **-1.22** | 2.34 | Decrease | -4.34 | 0.00033574 | 0.0679 | TC550497 | Q8R4C3 (Q8R4C3) Csr1, partial (40%) [TC550497] | |
| **1.16** | 2.24 | Increase | 4.32 | 0.00034971 | 0.0690 | M16349 | Rat asialoglycoprotein receptor RHL1 mRNA, 5' end. [M16349] | M16349 |
| **-1.73** | 3.31 | Decrease | -4.32 | 0.00035276 | 0.0692 | BF564899 | BF564899 UI-R-BU0-ana-b-07-0-UI.r1 UI-R-BU0 Rattus norvegicus cDNA clone UI-R-BU0-ana-b-07-0-UI 5', mRNA sequence [BF564899] | BF564899 |
| **-2.06** | 4.16 | Decrease | -4.31 | 0.00035858 | 0.0693 | DV719627 | RVL10432 Wackym-Soares normalized rat vestibular cDNA library Rattus norvegicus cDNA 5', mRNA sequence [DV719627] | DV719627 |
| **-1.05** | 2.07 | Decrease | -4.30 | 0.00036884 | 0.0696 | similar to CG3064-PB (predicted) | PREDICTED: Rattus norvegicus similar to CG3064-PB (predicted) (RGD1564844_predicted), mRNA [XM_236851] | XM_236851 |

| | | | | | | | | |
|---|---|---|---|---|---|---|---|---|
| **-1.73** | 3.31 | Decrease | -4.30 | 0.00037217 | 0.0696 | BF558804 | BF558804 UI-R-A1-du-a-12-0-UI.r1 UI-R-A1 Rattus norvegicus cDNA clone UI-R-A1-du-a-12-0-UI 5', mRNA sequence [BF558804] | BF558804 |
| **-1.41** | 2.65 | Decrease | -4.29 | 0.00037713 | 0.0697 | hypothetical protein LOC690208 | PREDICTED: Rattus norvegicus hypothetical protein LOC690208 (LOC690208), mRNA [XM_001073671] | XM_001073671 |
| **-1.39** | 2.63 | Decrease | -4.28 | 0.00038644 | 0.0708 | procollagen, type IV, alpha 3 (Goodpasture antigen) binding protein (predicted) | PREDICTED: Rattus norvegicus procollagen, type IV, alpha 3 (Goodpasture antigen) binding protein (predicted) (Col4a3bp_predicted), mRNA [XM_345143] | XM_345143 |
| **1.56** | 2.95 | Increase | 4.26 | 0.00039962 | 0.0718 | ectonucleotide pyrophosphatase/phosphodiesterase 2 | Rattus norvegicus ectonucleotide pyrophosphatase/phosphodiesterase 2 (Enpp2), mRNA [NM_057104] | NM_057104 |
| **-1.96** | 3.90 | Decrease | -4.26 | 0.00040093 | 0.0718 | similar to RIKEN cDNA 4931426K16 gene | Rattus norvegicus similar to RIKEN cDNA 4931426K16 gene (RGD1311267), mRNA [NM_001039024] | NM_001039024 |
| **1.21** | 2.32 | Increase | 4.26 | 0.0004038 | 0.0720 | cholecystokinin | Rattus norvegicus cholecystokinin (Cck), mRNA [NM_012829] | NM_012829 |
| **1.21** | 2.32 | Increase | 4.25 | 0.00041561 | 0.0732 | gamma-aminobutyric acid (GABA) B receptor 1 | Rattus norvegicus gamma-aminobutyric acid (GABA) B receptor 1 (Gabbr1), mRNA [NM_031028] | NM_031028 |
| **1.48** | 2.80 | Increase | 4.22 | 0.00044035 | 0.0738 | PHD finger protein 7 | Rattus norvegicus PHD finger protein 7 (Phf7), mRNA [NM_001012211] | NM_001012211 |
| **-1.18** | 2.26 | Decrease | -4.21 | 0.00045725 | 0.0759 | TC520546 | K13A_MOUSE (Q9EQW7) Kinesin-like protein KIF13A, partial (3%) [TC520546] | |
| **1.38** | 2.60 | Increase | 4.20 | 0.00046518 | 0.0759 | BG671620 | DRNBVF01 Rat DRG Library Rattus norvegicus cDNA clone DRNBVF01 5', mRNA sequence [BG671620] | BG671620 |

| | | | | | | | | |
|---|---|---|---|---|---|---|---|---|
| **1.24** | 2.36 | Increase | 4.16 | 0.00050398 | 0.0801 | ADP-ribosylation factor 6 | Rattus norvegicus ADP-ribosylation factor 6 (Arf6), mRNA [NM_024152] | NM_024152 |
| **1.49** | 2.81 | Increase | 4.15 | 0.00051937 | 0.0814 | adaptor protein complex AP-1, sigma 1 (predicted) | PREDICTED: Rattus norvegicus adaptor protein complex AP-1, sigma 1 (predicted) (Ap1s1_predicted), mRNA [XM_341052] | XM_341052 |
| **-1.73** | 3.31 | Decrease | -4.14 | 0.00053057 | 0.0817 | cadmium-inducible gene 1L | Rattus norvegicus cadmium-inducible gene 1L (Cdig1l), mRNA [NM_153623] | NM_153623 |
| **1.38** | 2.61 | Increase | 4.13 | 0.00054924 | 0.0823 | aquaporin 2 | Rattus norvegicus aquaporin 2 (Aqp2), mRNA [NM_012909] | NM_012909 |
| **3.06** | 8.32 | Increase | 4.10 | 0.00058874 | 0.0840 | plasticity-related protein PRG-2 | Rattus norvegicus plasticity-related protein PRG-2 (Prg-2), mRNA [NM_181634] | NM_181634 |
| **1.91** | 3.75 | Increase | 4.09 | 0.00059779 | 0.0848 | similar to JM11 protein (predicted) | PREDICTED: Rattus norvegicus similar to JM11 protein (predicted) (RGD1561596_predicted), mRNA [XM_228761] | XM_228761 |
| **-1.35** | 2.55 | Decrease | -4.05 | 0.00065577 | 0.0898 | PHD finger protein 17 (predicted) | PREDICTED: Rattus norvegicus PHD finger protein 17 (predicted) (Phf17_predicted), mRNA [XM_227074] | XM_227074 |
| **-1.33** | 2.52 | Decrease | -4.05 | 0.00066161 | 0.0898 | speedy homolog 1 (Drosophila) | Rattus norvegicus speedy homolog 1 (Drosophila) (Spdy1), mRNA [NM_138855] | NM_138855 |
| **-2.30** | 4.93 | Decrease | -4.04 | 0.00066878 | 0.0898 | LOC682316 | PREDICTED: Rattus norvegicus similar to dystrophin, muscular dystrophy (LOC682316), mRNA [XM_001060977] | XM_001060977 |
| **2.58** | 5.98 | Increase | 4.03 | 0.00069304 | 0.0920 | AI145447 | AI145447 UI-R-BT0-pv-d-09-0-UI.s1 UI-R-BT0 Rattus norvegicus cDNA clone UI-R-BT0-pv-d-09-0-UI 3', mRNA sequence [AI145447] | AI145447 |

| | | | | | | | | |
|---|---|---|---|---|---|---|---|---|
| **-1.18** | 2.27 | Decrease | -4.02 | 0.00070638 | 0.0935 | Obscn | PREDICTED: Rattus norvegicus obscurin, cytoskeletal calmodulin and titin-interacting RhoGEF (Obscn), mRNA [XM_001076876] | XM_001076876 |
| **-1.18** | 2.27 | Decrease | -4.00 | 0.00073898 | 0.0959 | zinc and ring finger 1 (predicted) | PREDICTED: Rattus norvegicus zinc and ring finger 1 (predicted) (Znrf1_predicted), mRNA [XM_342692] | XM_342692 |
| **1.07** | 2.10 | Increase | 3.98 | 0.00076446 | 0.0973 | hairy and enhancer of split 7 (Drosophila) (predicted) | PREDICTED: Rattus norvegicus hairy and enhancer of split 7 (Drosophila) (predicted) (Hes7_predicted), mRNA [XM_220597] | XM_220597 |
| **-1.45** | 2.73 | Decrease | -3.98 | 0.00076636 | 0.0973 | synaptojanin 1 | PREDICTED: Rattus norvegicus synaptojanin 1 (Synj1), mRNA [XM_573256] | XM_573256 |
| **-1.27** | 2.41 | Decrease | -3.96 | 0.00080322 | 0.0998 | chromodomain helicase DNA binding protein 1 (predicted) | PREDICTED: Rattus norvegicus chromodomain helicase DNA binding protein 1 (predicted) (Chd1_predicted), mRNA [XM_001056703] | XM_001056703 |
| **4.28** | 19.38 | Increase | 3.96 | 0.00080346 | 0.0998 | RGD1562348_predicted | PREDICTED: Rattus norvegicus similar to ankyrin repeat domain protein 17 isoform b (predicted) (RGD1562348_predicted), mRNA [XM_214012] | XM_214012 |
| **-1.65** | 3.14 | Decrease | -3.95 | 0.00081997 | 0.1002 | G protein-coupled receptor associated sorting protein 1 | Rattus norvegicus G protein-coupled receptor associated sorting protein 1 (Gprasp1), mRNA [NM_134386] | NM_134386 |
| **2.52** | 5.74 | Increase | 3.94 | 0.00084378 | 0.1003 | cell division cycle 20 homolog (S. cerevisiae) | Rattus norvegicus cell division cycle 20 homolog (S. cerevisiae) (Cdc20), mRNA [NM_171993] | NM_171993 |
| **2.12** | 4.35 | Increase | 3.93 | 0.0008566 | 0.1010 | TC522745 | Q7S3E7 (Q7S3E7) Predicted protein, partial (3%) [TC522745] | |

| | | | | | | | | |
|---|---|---|---|---|---|---|---|---|
| **-1.10** | 2.15 | Decrease | -3.92 | 0.00088171 | 0.1024 | XM_223541 | Rattus norvegicus similar to leucine zipper-EF-hand containing transmembrane protein 1 (LOC305457), mRNA [XM_223541] | XM_223541 |
| **1.33** | 2.52 | Increase | 3.91 | 0.00090969 | 0.1039 | procollagen, type 1, alpha 1 | PREDICTED: Rattus norvegicus procollagen, type 1, alpha 1 (Col1a1), mRNA [XM_213440] | XM_213440 |
| **1.04** | 2.06 | Increase | 3.91 | 0.00091381 | 0.1041 | CF110073 | Shultzomica03324 Rat lung airway and parenchyma cDNA libraries Rattus norvegicus cDNA clone Contig2930 5', mRNA sequence [CF110073] | CF110073 |
| **1.58** | 2.98 | Increase | 3.90 | 0.00091995 | 0.1045 | receptor (calcitonin) activity modifying protein 2 | Rattus norvegicus receptor (calcitonin) activity modifying protein 2 (Ramp2), mRNA [NM_031646] | NM_031646 |
| **1.27** | 2.41 | Increase | 3.90 | 0.00092814 | 0.1046 | mitochondrial tumor suppressor 1 | Rattus norvegicus mitochondrial tumor suppressor 1, mRNA (cDNA clone IMAGE:7104732), complete cds. [BC072537] | BC072537 |
| **-2.50** | 5.64 | Decrease | -3.88 | 0.00098027 | 0.1066 | homeodomain interacting protein kinase 2 (predicted) | Rattus norvegicus similar to nuclear body associated kinase 1a (LOC362342), mRNA [XM_342662] | XM_342662 |
| **-1.47** | 2.77 | Decrease | -3.87 | 0.00099969 | 0.1074 | stonin 2 (predicted) | PREDICTED: Rattus norvegicus stonin 2 (predicted) (Ston2_predicted), mRNA [XM_234454] | XM_234454 |
| **-1.61** | 3.05 | Decrease | -3.86 | 0.00101486 | 0.1080 | putative aminopeptidase Fxna | Rattus norvegicus putative aminopeptidase Fxna (Fxna), mRNA [NM_184050] | NM_184050 |
| **-1.16** | 2.24 | Decrease | -3.84 | 0.00105296 | 0.1082 | similar to NFkB interacting protein 1 | PREDICTED: Rattus norvegicus similar to NFkB interacting protein 1 (LOC686781), mRNA [XM_001075705] | XM_001075705 |

| | | | | | | | | |
|---|---|---|---|---|---|---|---|---|
| **-1.01** | 2.02 | Decrease | -3.84 | 0.00105614 | 0.1083 | LOC682690 | PREDICTED: Rattus norvegicus similar to chromodomain helicase DNA binding protein 9, transcript variant 1 (LOC682690), mRNA [XM_001062637] | XM_001062637 |
| **1.23** | 2.34 | Increase | 3.84 | 0.00107011 | 0.1089 | AW530281 | UI-R-BU0-amu-g-07-0-UI.s1 UI-R-BU0 Rattus norvegicus cDNA clone UI-R-BU0-amu-g-07-0-UI 3', mRNA sequence [AW530281] | AW530281 |
| **-1.04** | 2.05 | Decrease | -3.82 | 0.0011046 | 0.1092 | Arsk | PREDICTED: Rattus norvegicus arylsulfatase K (Arsk), mRNA [XM_001059083] | XM_001059083 |
| **2.11** | 4.33 | Increase | 3.82 | 0.00110929 | 0.1094 | similar to HOX11L2 (predicted) | PREDICTED: Rattus norvegicus similar to HOX11L2 (predicted) (RGD1564190_predicted), mRNA [XM_573065] | XM_573065 |
| **1.02** | 2.03 | Increase | 3.82 | 0.00111461 | 0.1094 | similar to Dynamin-binding protein (Scaffold protein Tuba) | PREDICTED: Rattus norvegicus similar to Dynamin-binding protein (Scaffold protein Tuba) (LOC309362), mRNA [XM_219860] | XM_219860 |
| **-1.12** | 2.17 | Decrease | -3.82 | 0.00111814 | 0.1094 | AW921244 | AW921244 EST352548 Rat gene index, normalized rat, norvegicus, Bento Soares Rattus norvegicus cDNA clone RGIHQ07 5' end, mRNA sequence [AW921244] | AW921244 |
| **-1.20** | 2.30 | Decrease | -3.81 | 0.00112796 | 0.1094 | XM_215481 | Rattus norvegicus similar to DEAH (Asp-Glu-Ala-His) box polypeptide 29; nucleic acid helicase DDXx (LOC294741), mRNA [XM_215481] | XM_215481 |
| **-1.39** | 2.62 | Decrease | -3.80 | 0.00115426 | 0.1103 | CASP8 and FADD-like apoptosis regulator | Rattus norvegicus CASP8 and FADD-like apoptosis regulator (Cflar), transcript variant 2, mRNA [NM_057138] | NM_057138 |

| | | | | | | | | |
|---|---|---|---|---|---|---|---|---|
| **-1.44** | 2.71 | Decrease | -3.79 | 0.00120723 | 0.1121 | AA997849 | UI-R-C0-hu-b-07-0-UI.s1 UI-R-C0 Rattus norvegicus cDNA clone UI-R-C0-hu-b-07-0-UI 3', mRNA sequence [AA997849] | AA997849 |
| **-1.62** | 3.08 | Decrease | -3.78 | 0.00120978 | 0.1121 | DV725773 | RVL17751 Wackym-Soares normalized rat vestibular cDNA library Rattus norvegicus cDNA 5', mRNA sequence [DV725773] | DV725773 |
| **-1.50** | 2.84 | Decrease | -3.76 | 0.00127446 | 0.1153 | AW917250 | AW917250 EST348554 Rat gene index, normalized rat, norvegicus, Bento Soares Rattus norvegicus cDNA clone RGIEB30 5' end, mRNA sequence [AW917250] | AW917250 |
| **1.65** | 3.14 | Increase | 3.76 | 0.00128684 | 0.1153 | forkhead-like 18 (Drosophila) | Rattus norvegicus forkhead-like 18 (Drosophila) (Fkhl18), mRNA [NM_001012091] | NM_001012091 |
| **1.14** | 2.21 | Increase | 3.75 | 0.00131277 | 0.1163 | similar to RIKEN cDNA 1300007B12; clone MNCb-2755 | Rattus norvegicus similar to RIKEN cDNA 1300007B12; clone MNCb-2755 (RGD1309892), mRNA [NM_001013871] | NM_001013871 |
| **-1.22** | 2.33 | Decrease | -3.75 | 0.00132285 | 0.1165 | BE117514 | BE117514 UI-R-BS1-ayo-f-11-0-UI.s1 UI-R-BS1 Rattus norvegicus cDNA clone UI-R-BS1-ayo-f-11-0-UI 3', mRNA sequence [BE117514] | BE117514 |
| **1.22** | 2.33 | Increase | 3.74 | 0.00135517 | 0.1179 | serologically defined colon cancer antigen 3 | Rattus norvegicus serologically defined colon cancer antigen 3 (Sdccag3), mRNA [NM_001013135] | NM_001013135 |
| **1.22** | 2.33 | Increase | 3.73 | 0.00138391 | 0.1191 | Poldip3_predicted | PREDICTED: Rattus norvegicus polymerase (DNA-directed), delta interacting protein 3 (predicted) (Poldip3_predicted), mRNA [XM_001077668] | XM_001077668 |

| | | | | | | | | |
|---|---|---|---|---|---|---|---|---|
| **1.34** | 2.54 | Increase | 3.72 | 0.00139849 | 0.1191 | procollagen, type I, alpha 2 | Rattus norvegicus procollagen, type I, alpha 2 (Col1a2), mRNA [NM_053356] | NM_053356 |
| **-1.04** | 2.06 | Decrease | -3.70 | 0.00145321 | 0.1213 | ankyrin 3, epithelial | Rattus norvegicus ankyrin 3, epithelial (Ank3), transcript variant 1, mRNA [NM_031805] | NM_031805 |
| **1.31** | 2.48 | Increase | 3.70 | 0.00146522 | 0.1216 | AI548160 | AI548160 UI-R-C3-su-a-08-0-UI.s1 UI-R-C3 Rattus norvegicus cDNA clone UI-R-C3-su-a-08-0-UI 3', mRNA sequence [AI548160] | AI548160 |
| **1.38** | 2.60 | Increase | 3.70 | 0.00147101 | 0.1219 | similar to Antxr2 protein | PREDICTED: Rattus norvegicus similar to Antxr2 protein (LOC305633), mRNA [XM_223745] | XM_223745 |
| **2.23** | 4.69 | Increase | 3.69 | 0.00148803 | 0.1224 | BI294724 | BI294724 UI-R-DK0-ceb-d-01-0-UI.s1 UI-R-DK0 Rattus norvegicus cDNA clone UI-R-DK0-ceb-d-01-0-UI 3', mRNA sequence [BI294724] | BI294724 |
| **-1.12** | 2.17 | Decrease | -3.69 | 0.0014957 | 0.1227 | similar to thyroid hormone receptor interactor 11 | PREDICTED: Rattus norvegicus similar to thyroid hormone receptor interactor 11 (LOC314393), mRNA [XM_001065684] | XM_001065684 |
| **-2.44** | 5.44 | Decrease | -3.66 | 0.00162166 | 0.1269 | epoxide hydrolase 1, microsomal | Rattus norvegicus epoxide hydrolase 1, microsomal (Ephx1), transcript variant 2, mRNA [NM_012844] | NM_012844 |
| **2.01** | 4.04 | Increase | 3.66 | 0.00162452 | 0.1269 | plakophilin 1 (predicted) | PREDICTED: Rattus norvegicus plakophilin 1 (predicted) (Pkp1_predicted), mRNA [XM_222666] | XM_222666 |
| **1.46** | 2.75 | Increase | 3.63 | 0.00172234 | 0.1301 | putative small membrane protein NID67 | Rattus norvegicus putative small membrane protein NID67 (Nid67), mRNA [NM_173126] | NM_173126 |
| **1.40** | 2.65 | Increase | 3.62 | 0.00174717 | 0.1308 | actin related protein 2/3 complex, subunit 4 (predicted) | PREDICTED: Rattus norvegicus actin related protein 2/3 complex, subunit | XM_238365 |

| | | | | | | | | |
|---|---|---|---|---|---|---|---|---|
| | | | | | | | 4 (predicted) (Arpc4_predicted), mRNA [XM_238365] | |
| **-1.13** | 2.18 | Decrease | -3.62 | 0.00175214 | 0.1309 | short form of beta II spectrin | Rattus norvegicus beta II spectrin-short isoform mRNA, partial cds. [AF218849] | AF218849 |
| **-1.42** | 2.67 | Decrease | -3.62 | 0.00176135 | 0.1309 | AW914836 | AW914836 EST346140 Normalized rat ovary, Bento Soares Rattus sp. cDNA clone RGIBH87 5' end, mRNA sequence [AW914836] | AW914836 |
| **-1.22** | 2.32 | Decrease | -3.62 | 0.00178164 | 0.1319 | AA926021 | AA926021 UI-R-A1-es-d-09-0-UI.s1 UI-R-A1 Rattus norvegicus cDNA clone UI-R-A1-es-d-09-0-UI 3' similar to gi [AA926021] | AA926021 |
| **1.26** | 2.40 | Increase | 3.61 | 0.00179104 | 0.1319 | TC539961 | Q986H9 (Q986H9) ABC transporter binding protein, partial (5%) [TC539961] | |
| **-1.19** | 2.29 | Decrease | -3.61 | 0.00180987 | 0.1323 | DV727624 | RVL20147 Wackym-Soares normalized rat vestibular cDNA library Rattus norvegicus cDNA 5', mRNA sequence [DV727624] | DV727624 |
| **-2.39** | 5.26 | Decrease | -3.59 | 0.00188425 | 0.1358 | TC522919 | Q6WZ94 (Q6WZ94) Transcriptional regulator, partial (6%) [TC522919] | |
| **-1.07** | 2.09 | Decrease | -3.59 | 0.00190887 | 0.1363 | CF107847 | Shultzomica01098 Rat lung airway and parenchyma cDNA libraries Rattus norvegicus cDNA clone NA8658 5', mRNA sequence [CF107847] | CF107847 |
| **-1.26** | 2.39 | Decrease | -3.59 | 0.00191382 | 0.1363 | AW918229 | AW918229 EST349533 Rat gene index, normalized rat, norvegicus, Bento Soares Rattus norvegicus cDNA clone RGIEO12 5' end, mRNA sequence [AW918229] | AW918229 |
| **-1.18** | 2.27 | Decrease | -3.56 | 0.0020184 | 0.1395 | similar to RNA polymerase III subunit RPC2 (predicted) | PREDICTED: Rattus norvegicus similar to RNA polymerase III subunit | XM_343188 |

| | | | | | | | | |
|---|---|---|---|---|---|---|---|---|
| | | | | | | | RPC2 (predicted) (RGD1565311_predicted), mRNA [XM_343188] | |
| **-1.74** | 3.34 | Decrease | -3.56 | 0.00202929 | 0.1396 | F-box only protein 17 | Rattus norvegicus F-box only protein 17 (Fbxo17), mRNA [NM_001013064] | NM_001013064 |
| **2.74** | 6.66 | Increase | 3.56 | 0.00203544 | 0.1398 | naked cuticle 1 homolog (Drosophila) (predicted) | PREDICTED: Rattus norvegicus naked cuticle 1 homolog (Drosophila) (predicted) (Nkd1_predicted), mRNA [XM_344728] | XM_344728 |
| **-1.76** | 3.38 | Decrease | -3.55 | 0.00206199 | 0.1401 | BF559590 | BF559590 UI-R-A1-dz-b-11-0-UI.r1 UI-R-A1 Rattus norvegicus cDNA clone UI-R-A1-dz-b-11-0-UI 5', mRNA sequence [BF559590] | BF559590 |
| **-2.02** | 4.06 | Decrease | -3.55 | 0.00206452 | 0.1401 | TC544106 | Q7PN93 (Q7PN93) ENSANGP00000023986 (Fragment), partial (17%) [TC544106] | |
| **-2.52** | 5.73 | Decrease | -3.54 | 0.00210711 | 0.1410 | transformed mouse 3T3 cell double minute 4 | Rattus norvegicus transformed mouse 3T3 cell double minute 4 (Mdm4), mRNA [NM_001012026] | NM_001012026 |
| **1.44** | 2.72 | Increase | 3.53 | 0.00217264 | 0.1426 | AI180270 | AI180270 EST224013 Normalized rat spleen, Bento Soares Rattus sp. cDNA clone RSPCS66 3' end, mRNA sequence [AI180270] | AI180270 |
| **3.15** | 8.86 | Increase | 3.52 | 0.00220238 | 0.1432 | XM_230900 | Rattus norvegicus similar to nuclear DNA binding factor (LOC296420), mRNA [XM_230900] | XM_230900 |
| **-1.38** | 2.60 | Decrease | -3.52 | 0.00221443 | 0.1432 | kinesin family member 1B | Rattus norvegicus kinesin family member 1B (Kif1b), mRNA [NM_057200] | NM_057200 |
| **-1.46** | 2.75 | Decrease | -3.52 | 0.00221887 | 0.1432 | REX4, RNA exonuclease 4 homolog (S. cerevisiae) | Rattus norvegicus REX4, RNA exonuclease 4 homolog (S. cerevisiae) (Rexo4), mRNA [NM_001033884] | NM_001033884 |

| | | | | | | | | |
|---|---|---|---|---|---|---|---|---|
| **-1.17** | 2.25 | Decrease | -3.52 | 0.00224096 | 0.1438 | AY623033 | Rattus norvegicus clone S33-A glycosyltransferase family 8-related mRNA, 3' UTR. [AY623033] | AY623033 |
| **1.50** | 2.84 | Increase | 3.50 | 0.00232474 | 0.1465 | LOC681211 | PREDICTED: Rattus norvegicus similar to monoacylglycerol O-acyltransferase 2 (LOC681211), mRNA [XM_001060766] | XM_001060766 |
| **-1.51** | 2.85 | Decrease | -3.50 | 0.0023524 | 0.1477 | TC530091 | Q7WGV9 (Q7WGV9) Sulfate transport system permease protein, partial (6%) [TC530091] | |
| **1.36** | 2.56 | Increase | 3.49 | 0.00237681 | 0.1486 | similar to hypothetical gene supported by BC007071 | Rattus norvegicus similar to hypothetical gene supported by BC007071 (RGD1311868), mRNA [NM_001033061] | NM_001033061 |
| **1.37** | 2.59 | Increase | 3.47 | 0.00249998 | 0.1498 | XM_222160 | Rattus norvegicus similar to endothelial monocyte-activating polypeptide (LOC288662), mRNA [XM_222160] | XM_222160 |
| **1.16** | 2.23 | Increase | 3.47 | 0.00250552 | 0.1498 | RGD1562717_predicted | PREDICTED: Rattus norvegicus similar to ABI gene family, member 3 (NESH) binding protein (predicted) (RGD1562717_predicted), mRNA [XM_344015] | XM_344015 |
| **-1.29** | 2.44 | Decrease | -3.46 | 0.0025342 | 0.1506 | pam, highwire, rpm 1 (predicted) | PREDICTED: Rattus norvegicus pam, highwire, rpm 1 (predicted) (Phr1_predicted), mRNA [XM_214245] | XM_214245 |
| **-1.01** | 2.01 | Decrease | -3.46 | 0.00257538 | 0.1518 | carbamyl phosphatate synthetase 2 (mapped) | PREDICTED: Rattus norvegicus carbamyl phosphatate synthetase 2 (mapped) (Cad_mapped), mRNA [XM_343027] | XM_343027 |
| **-1.47** | 2.77 | Decrease | -3.45 | 0.00258624 | 0.1520 | zinc and ring finger 1 (predicted) | PREDICTED: Rattus norvegicus zinc and ring finger 1 (predicted) | XM_342692 |

| | | | | | | | | |
|---|---|---|---|---|---|---|---|---|
| | | | | | | | (Znrf1_predicted), mRNA [XM_342692] | |
| **3.57** | 11.85 | Increase | 3.45 | 0.00259159 | 0.1521 | a disintegrin-like and metallopeptidase (reprolysin type) with thrombospondin type 1 motif, 5 (aggrecanase-2) | Rattus norvegicus a disintegrin-like and metallopeptidase (reprolysin type) with thrombospondin type 1 motif, 5 (aggrecanase-2) (Adamts5), mRNA [NM_198761] | NM_198761 |
| **-2.34** | 5.08 | Decrease | -3.45 | 0.00261467 | 0.1528 | citron | Rattus norvegicus postsynaptic density protein (citron) mRNA, complete cds. [AF039218] | AF039218 |
| **1.62** | 3.07 | Increase | 3.45 | 0.00262671 | 0.1528 | TC543377 | P97374 (P97374) Serum amyloid A protein isoform 1, partial (51%) [TC543377] | |
| **1.09** | 2.13 | Increase | 3.44 | 0.00269206 | 0.1544 | D-serine modulator-1 | Rattus norvegicus D-serine modulator-1 (Dsm-1), mRNA [NM_001037215] | NM_001037215 |
| **1.88** | 3.68 | Increase | 3.43 | 0.00271635 | 0.1549 | guanylate cyclase 2g | Rattus norvegicus ksGC mRNA, complete cds. [U33847] | U33847 |
| **-1.35** | 2.55 | Decrease | -3.43 | 0.00272039 | 0.1549 | non imprinted in Prader-Willi/Angelman syndrome 2 homolog (human) (predicted) | PREDICTED: Rattus norvegicus non imprinted in Prader-Willi/Angelman syndrome 2 homolog (human) (predicted) (Nipa2_predicted), mRNA [XM_218718] | XM_218718 |
| **-1.39** | 2.62 | Decrease | -3.43 | 0.00276211 | 0.1549 | DV727231 | RVL19689 Wackym-Soares normalized rat vestibular cDNA library Rattus norvegicus cDNA 5', mRNA sequence [DV727231] | DV727231 |
| **1.07** | 2.09 | Increase | 3.42 | 0.00276402 | 0.1549 | XM_230113 | Rattus norvegicus similar to olfactory receptor GA_x6K02T2Q125-48226321-48225386 (LOC295766), mRNA [XM_230113] | XM_230113 |
| **1.03** | 2.05 | Increase | 3.42 | 0.00276469 | 0.1549 | hypothetical protein LOC687595 | PREDICTED: Rattus norvegicus hypothetical protein LOC687594, | XM_001078994 |

| | | | | | | | | |
|---|---|---|---|---|---|---|---|---|
| | | | | | | | transcript variant 3 (LOC687595), mRNA [XM_001078994] | |
| **1.25** | 2.38 | Increase | 3.42 | 0.00278419 | 0.1552 | capping protein (actin filament), gelsolin-like | Rattus norvegicus capping protein (actin filament), gelsolin-like (Capg), mRNA [NM_001013086] | NM_001013086 |
| **1.07** | 2.09 | Increase | 3.42 | 0.00279197 | 0.1552 | RAB13, member RAS oncogene family | Rattus norvegicus RAB13, member RAS oncogene family (Rab13), mRNA [NM_031092] | NM_031092 |
| **-1.57** | 2.97 | Decrease | -3.42 | 0.00279685 | 0.1552 | similar to KIAA1582 protein (predicted) | AA956352 UI-R-E1-fj-d-03-0-UI.s1 UI-R-E1 Rattus norvegicus cDNA clone UI-R-E1-fj-d-03-0-UI 3' similar to gi [AA956352] | AA956352 |
| **1.97** | 3.91 | Increase | 3.42 | 0.0028009 | 0.1552 | oligodendrocyte transcription factor 3 (predicted) | Rattus norvegicus similar to Olig3 bHLH protein (LOC293012), mRNA [XM_218772] | XM_218772 |
| **-1.96** | 3.90 | Decrease | -3.41 | 0.00286925 | 0.1573 | gap junction membrane channel protein alpha 5 | Rattus norvegicus gap junction membrane channel protein alpha 5 (Gja5), mRNA [NM_019280] | NM_019280 |
| **1.43** | 2.69 | Increase | 3.40 | 0.00289194 | 0.1583 | AW507178 | AW507178 EST00605 Plasmid Subtractive Library of Rat Cerebrum (stroke) Rattus norvegicus cDNA clone 1stB1, mRNA sequence [AW507178] | AW507178 |
| **2.42** | 5.35 | Increase | 3.40 | 0.00290391 | 0.1586 | mitochondrial ribosomal protein S10 | Rattus norvegicus mitochondrial ribosomal protein S10 (Mrps10), mRNA [NM_001008859] | NM_001008859 |
| **-1.79** | 3.46 | Decrease | -3.40 | 0.00291347 | 0.1589 | Mediterranean fever | Rattus norvegicus Mediterranean fever (Mefv), mRNA [NM_031634] | NM_031634 |
| **1.43** | 2.69 | Increase | 3.40 | 0.00291694 | 0.1589 | G-protein signalling modulator 1 (AGS3-like, C. elegans) | Rattus norvegicus G-protein signalling modulator 1 (AGS3-like, C. elegans), mRNA (cDNA clone IMAGE:7104788), complete cds. [BC086535] | BC086535 |

| | | | | | | | | |
|---|---|---|---|---|---|---|---|---|
| **1.59** | 3.00 | Increase | 3.39 | 0.00299458 | 0.1599 | olfactory receptor 1294 (predicted) | Rattus norvegicus olfactory receptor 1294 (predicted) (Olr1294_predicted), mRNA [NM_001000597] | NM_001000597 |
| **-2.14** | 4.40 | Decrease | -3.39 | 0.00299963 | 0.1600 | zinc finger protein 365 | Rattus norvegicus zinc finger protein 365 (Zfp365), mRNA [NM_001025145] | NM_001025145 |
| **-1.96** | 3.89 | Decrease | -3.39 | 0.0030178 | 0.1603 | transferrin receptor | PREDICTED: Rattus norvegicus transferrin receptor (Tfrc), mRNA [XM_001072774] | XM_001072774 |
| **1.42** | 2.68 | Increase | 3.38 | 0.00303493 | 0.1606 | G protein pathway suppressor 2 (predicted) | PREDICTED: Rattus norvegicus G protein pathway suppressor 2 (predicted) (Gps2_predicted), mRNA [XM_220615] | XM_220615 |
| **-1.46** | 2.75 | Decrease | -3.38 | 0.00306101 | 0.1610 | similar to hypothetical protein (predicted) | PREDICTED: Rattus norvegicus similar to hypothetical protein (predicted) (RGD1305269_predicted), mRNA [XM_223397] | XM_223397 |
| **-2.40** | 5.30 | Decrease | -3.37 | 0.00309934 | 0.1611 | ryanodine receptor 1, skeletal muscle | PREDICTED: Rattus norvegicus similar to ryanodine receptor 1 (skeletal) (LOC686059), mRNA [XM_574411] | XM_574411 |
| **1.12** | 2.18 | Increase | 3.37 | 0.00310347 | 0.1611 | insulin-like growth factor binding protein 5 | Rattus norvegicus cDNA clone IMAGE:7110383 [BC087030] | BC087030 |
| **1.27** | 2.41 | Increase | 3.37 | 0.00314157 | 0.1621 | BM986267 | BM986267 EST531721 Rat gene index, normalized rat, norvegicus Rattus norvegicus cDNA clone RGIAD36 3' end similar to CaM kinase IV beta, mRNA sequence [BM986267] | BM986267 |
| **-1.31** | 2.48 | Decrease | -3.37 | 0.00315777 | 0.1621 | ubiquitin specific peptidase 9, X chromosome (predicted) | PREDICTED: Rattus norvegicus ubiquitin specific peptidase 9, X chromosome (predicted) | XM_343766 |

| | | | | | | | | |
|---|---|---|---|---|---|---|---|---|
| | | | | | | | (Usp9x_predicted), mRNA [XM_343766] | |
| **1.80** | 3.48 | Increase | 3.37 | 0.00316515 | 0.1621 | similar to glyceraldehyde-3-phosphate dehydrogenase (predicted) | PREDICTED: Rattus norvegicus similar to glyceraldehyde-3-phosphate dehydrogenase (predicted) (RGD1565190_predicted), mRNA [XM_344998] | XM_344998 |
| **-2.20** | 4.58 | Decrease | -3.36 | 0.00317576 | 0.1621 | DY471722 | RVL22932 Wackym-Soares normalized rat vestibular cDNA library Rattus norvegicus cDNA 5', mRNA sequence [DY471722] | DY471722 |
| **-1.14** | 2.20 | Decrease | -3.36 | 0.00322584 | 0.1627 | hypothetical protein LOC304743 | Rattus norvegicus hypothetical protein LOC304743 (LOC304743), mRNA [NM_001024997] | NM_001024997 |
| **1.50** | 2.82 | Increase | 3.36 | 0.00324062 | 0.1631 | lysyl oxidase-like 2 (predicted) | PREDICTED: Rattus norvegicus lysyl oxidase-like 2 (predicted) (Loxl2_predicted), mRNA [XM_214225] | XM_214225 |
| **-1.33** | 2.52 | Decrease | -3.35 | 0.00330146 | 0.1645 | S-adenosylhomocysteine hydrolase-like 1 (predicted) | PREDICTED: Rattus norvegicus S-adenosylhomocysteine hydrolase-like 1 (predicted) (Ahcyl1_predicted), mRNA [XM_001068488] | XM_001068488 |
| **1.08** | 2.11 | Increase | 3.34 | 0.00332289 | 0.1649 | similar to Chromobox protein homolog 4 (Polycomb 2 homolog) (Pc2) (MPc2) | PREDICTED: Rattus norvegicus similar to Chromobox protein homolog 4 (Polycomb 2 homolog) (Pc2) (MPc2) (LOC501403), mRNA [XM_001081757] | XM_001081757 |
| **-1.50** | 2.83 | Decrease | -3.34 | 0.00334268 | 0.1649 | similar to neurobeachin (predicted) | PREDICTED: Rattus norvegicus similar to neurobeachin (predicted) (RGD1562629_predicted), mRNA [XM_001059612] | XM_001059612 |
| **1.62** | 3.08 | Increase | 3.34 | 0.00334331 | 0.1649 | peripherin 1 | Rattus norvegicus peripherin 1 (Prph1), mRNA [NM_012633] | NM_012633 |

| | | | | | | | | |
|---|---|---|---|---|---|---|---|---|
| **-5.24** | 37.82 | Decrease | -3.34 | 0.00336991 | 0.1657 | AA956504 | AA956504 UI-R-E1-fk-g-05-0-UI.s1 UI-R-E1 Rattus norvegicus cDNA clone UI-R-E1-fk-g-05-0-UI 3' similar to gi [AA956504] | AA956504 |
| **-1.39** | 2.62 | Decrease | -3.34 | 0.00338578 | 0.1657 | DEAH (Asp-Glu-Ala-His) box polypeptide 9 (predicted) | PREDICTED: Rattus norvegicus DEAH (Asp-Glu-Ala-His) box polypeptide 9 (predicted) (Dhx9_predicted), mRNA [XM_239780] | XM_239780 |
| **-1.08** | 2.11 | Decrease | -3.33 | 0.00339987 | 0.1657 | BC089934 | Rattus norvegicus cDNA clone IMAGE:7309127 [BC089934] | BC089934 |
| **-1.02** | 2.03 | Decrease | -3.33 | 0.00342911 | 0.1659 | HtrA serine peptidase 4 (predicted) | Rattus norvegicus similar to Probable serine protease HTRA4 precursor (LOC306564), mRNA [XM_224963] | XM_224963 |
| **1.06** | 2.08 | Increase | 3.33 | 0.00345874 | 0.1663 | 3-alpha-hydroxysteroid dehydrogenase | Rattus norvegicus 3-alpha-hydroxysteroid dehydrogenase (LOC191574), mRNA [NM_138547] | NM_138547 |
| **1.22** | 2.32 | Increase | 3.32 | 0.00348943 | 0.1667 | RGD1564450 (predicted) | PREDICTED: Rattus norvegicus RGD1564450 (predicted) (RGD1564450_predicted), mRNA [XM_215355] | XM_215355 |
| **-1.01** | 2.02 | Decrease | -3.32 | 0.00354795 | 0.1679 | TC521816 | D87060 cerebral protein-1 {Homo sapiens;} , partial (17%) [TC521816] | |
| **1.21** | 2.31 | Increase | 3.30 | 0.00365531 | 0.1703 | BF555594 | BF555594 UI-R-E0-cz-b-01-0-UI.r1 UI-R-E0 Rattus norvegicus cDNA clone UI-R-E0-cz-b-01-0-UI 5', mRNA sequence [BF555594] | BF555594 |
| **1.04** | 2.06 | Increase | 3.30 | 0.00366227 | 0.1703 | similar to Hypothetical protein MGC59076 | Rattus norvegicus similar to Hypothetical protein MGC59076 (RGD1309144), mRNA [NM_001009658] | NM_001009658 |
| **2.19** | 4.57 | Increase | 3.30 | 0.00369731 | 0.1711 | mitochondrial ribosomal protein L52 (predicted) | PREDICTED: Rattus norvegicus mitochondrial ribosomal protein L52 (predicted), transcript variant 2 | XM_341312 |

| | | | | | | | | |
|---|---|---|---|---|---|---|---|---|
| | | | | | | | (Mrpl52_predicted), mRNA [XM_341312] | |
| **-1.39** | 2.62 | Decrease | -3.30 | 0.00370888 | 0.1713 | potassium inwardly-rectifying channel, subfamily J, member 13 | Rattus norvegicus potassium inwardly-rectifying channel, subfamily J, member 13 (Kcnj13), mRNA [NM_053608] | NM_053608 |
| **-5.55** | 46.95 | Decrease | -3.29 | 0.00371585 | 0.1713 | tachykinin 4 | Rattus norvegicus tachykinin 4 (Tac4), mRNA [NM_172328] | NM_172328 |
| **-3.70** | 13.02 | Decrease | -3.29 | 0.0037495 | 0.1716 | discs, large (Drosophila) homolog-associated protein 3 | Rattus norvegicus discs, large (Drosophila) homolog-associated protein 3 (Dlgap3), mRNA [NM_173138] | NM_173138 |
| **1.13** | 2.19 | Increase | 3.29 | 0.00379421 | 0.1725 | AI599392 | AI599392 EST251095 Normalized rat embryo, Bento Soares Rattus sp. cDNA clone REMEO50 3' end, mRNA sequence [AI599392] | AI599392 |
| **1.02** | 2.03 | Increase | 3.29 | 0.00379446 | 0.1725 | endothelial cell adhesion molecule | Rattus norvegicus endothelial cell adhesion molecule (Esam), mRNA [NM_001004245] | NM_001004245 |
| **1.16** | 2.23 | Increase | 3.27 | 0.00390879 | 0.1737 | procollagen, type 1, alpha 1 | Rat alpha-1 type I collagen mRNA, segment 3. [M12199] | M12199 |
| **-1.62** | 3.07 | Decrease | -3.27 | 0.0039246 | 0.1737 | Syncrip | PREDICTED: Rattus norvegicus synaptotagmin binding, cytoplasmic RNA interacting protein (Syncrip), mRNA [XM_001065902] | XM_001065902 |
| **1.03** | 2.04 | Increase | 3.26 | 0.00404379 | 0.1759 | hypothetical protein LOC690113 | PREDICTED: Rattus norvegicus similar to hypothetical protein FLJ14800 (predicted) (RGD1561500_predicted), mRNA [XM_235711] | XM_235711 |
| **-1.64** | 3.12 | Decrease | -3.26 | 0.00404615 | 0.1759 | hypothetical LOC288978 | PREDICTED: Rattus norvegicus hypothetical LOC288978 (LOC288978), mRNA [XM_213864] | XM_213864 |

| | | | | | | | | |
|---|---|---|---|---|---|---|---|---|
| **1.02** | 2.02 | Increase | 3.26 | 0.00406073 | 0.1759 | CTD (carboxy-terminal domain, RNA polymerase II, polypeptide A) small phosphatase 1 | PREDICTED: Rattus norvegicus CTD (carboxy-terminal domain, RNA polymerase II, polypeptide A) small phosphatase 1 (Ctdsp1), mRNA [XM_343588] | XM_343588 |
| **-1.88** | 3.68 | Decrease | -3.25 | 0.00409773 | 0.1765 | BM986233 | BM986233 EST531687 Rat gene index, normalized rat, norvegicus Rattus norvegicus cDNA clone RGIAB50 3' end similar to 57-KDa calcium-binding protein MCaBP, mRNA sequence [BM986233] | BM986233 |
| **-2.02** | 4.07 | Decrease | -3.25 | 0.00410651 | 0.1765 | Ttn | PREDICTED: Rattus norvegicus titin (Ttn), mRNA [XM_001065955] | XM_001065955 |
| **1.95** | 3.86 | Increase | 3.25 | 0.00415592 | 0.1771 | ChaC, cation transport regulator-like 1 (E. coli) (predicted) | PREDICTED: Rattus norvegicus ChaC, cation transport regulator-like 1 (E. coli) (predicted) (Chac1_predicted), mRNA [XM_342497] | XM_342497 |
| **-1.07** | 2.10 | Decrease | -3.24 | 0.00418276 | 0.1778 | similar to RIKEN cDNA E230015L20 gene (predicted) | PREDICTED: Rattus norvegicus similar to RIKEN cDNA E230015L20 gene (predicted) (RGD1560873_predicted), mRNA [XM_001058121] | XM_001058121 |
| **1.58** | 2.99 | Increase | 3.24 | 0.00418398 | 0.1778 | olfactory receptor 384 (predicted) | Rattus norvegicus olfactory receptor 384 (predicted) (Olr384_predicted), mRNA [NM_001000263] | NM_001000263 |
| **1.42** | 2.68 | Increase | 3.24 | 0.00424019 | 0.1789 | cytochrome b-245, alpha polypeptide | Rattus norvegicus cytochrome b-245, alpha polypeptide (Cyba), mRNA [NM_024160] | NM_024160 |
| **-1.68** | 3.20 | Decrease | -3.22 | 0.00439272 | 0.1823 | oxidative-stress responsive 1 (predicted) | PREDICTED: Rattus norvegicus oxidative-stress responsive 1 (predicted) (Oxsr1_predicted), mRNA [XM_001078098] | XM_001078098 |
| **-1.13** | 2.19 | Decrease | -3.22 | 0.00440058 | 0.1823 | tenascin R | Rattus norvegicus tenascin R (Tnr), mRNA [NM_013045] | NM_013045 |

| | | | | | | | | |
|---|---|---|---|---|---|---|---|---|
| **-1.15** | 2.22 | Decrease | -3.22 | 0.00445323 | 0.1823 | AW141160 | AW141160 EST291196 Normalized rat brain, Bento Soares Rattus sp. cDNA clone RGIBE24 5' end similar to Ki-ras cellular oncogene 4B, mRNA sequence [AW141160] | AW141160 |
| **-1.11** | 2.16 | Decrease | -3.22 | 0.00445436 | 0.1823 | similar to RIKEN cDNA 3110052N05 | Rattus norvegicus similar to RIKEN cDNA 3110052N05 (RGD1308579), mRNA [NM_001014151] | NM_001014151 |
| **-2.63** | 6.18 | Decrease | -3.21 | 0.00455128 | 0.1837 | dystrophin, muscular dystrophy | Rattus norvegicus dystrophin, muscular dystrophy (Dmd), transcript variant Dp71ab, mRNA [NM_001005244] | NM_001005244 |
| **1.53** | 2.89 | Increase | 3.20 | 0.00456528 | 0.1837 | BI282748 | BI282748 UI-R-CW0s-cce-g-02-0-UI.s1 UI-R-CW0s Rattus norvegicus cDNA clone UI-R-CW0s-cce-g-02-0-UI 3', mRNA sequence [BI282748] | BI282748 |
| **1.80** | 3.47 | Increase | 3.20 | 0.00457862 | 0.1839 | signal sequence receptor, gamma | Rattus norvegicus signal sequence receptor, gamma (Ssr3), mRNA [NM_031120] | NM_031120 |
| **-4.62** | 24.52 | Decrease | -3.20 | 0.00460328 | 0.1844 | similar to apolipoprotein L2; apolipoprotein L-II (predicted) | PREDICTED: Rattus norvegicus similar to apolipoprotein L2; apolipoprotein L-II (predicted) (RGD1309808_predicted), mRNA [XM_343282] | XM_343282 |
| **-1.07** | 2.10 | Decrease | -3.20 | 0.00460798 | 0.1844 | similar to KIAA0614 protein (predicted) | PREDICTED: Rattus norvegicus similar to KIAA0614 protein (predicted) (RGD1309762_predicted), mRNA [XM_222205] | XM_222205 |
| **-1.50** | 2.82 | Decrease | -3.20 | 0.00464147 | 0.1853 | cyclin G1 | Rattus norvegicus cyclin G1 (Ccng1), mRNA [NM_012923] | NM_012923 |
| **-1.04** | 2.05 | Decrease | -3.19 | 0.00466874 | 0.1853 | Arid2_predicted | PREDICTED: Rattus norvegicus AT rich interactive domain 2 (Arid-rfx | XM_001059099 |

| | | | | | | | | |
|---|---|---|---|---|---|---|---|---|
| | | | | | | | like) (predicted) (Arid2_predicted), mRNA [XM_001059099] | |
| **1.77** | 3.42 | Increase | 3.19 | 0.00470476 | 0.1855 | similar to Protein C10orf11 homolog | PREDICTED: Rattus norvegicus similar to Protein C10orf11 homolog (LOC681383), mRNA [XM_001059639] | XM_001059639 |
| **3.31** | 9.95 | Increase | 3.19 | 0.00471651 | 0.1857 | AA924846 | AA924846 UI-R-A1-eb-a-03-0-UI.s1 UI-R-A1 Rattus norvegicus cDNA clone UI-R-A1-eb-a-03-0-UI 3' similar to gi [AA924846] | AA924846 |
| **-1.87** | 3.66 | Decrease | -3.19 | 0.00476375 | 0.1873 | ribonuclease, RNase A family, 10 (non-active) (predicted) | Rattus norvegicus ribonuclease, RNase A family, 10 (non-active) (predicted) (Rnase10_predicted), mRNA [NM_001012467] | NM_001012467 |
| **1.14** | 2.20 | Increase | 3.18 | 0.00480234 | 0.1885 | BE108178 | BE108178 UI-R-BS1-ayw-g-07-0-UI.s1 UI-R-BS1 Rattus norvegicus cDNA clone UI-R-BS1-ayw-g-07-0-UI 3', mRNA sequence [BE108178] | BE108178 |
| **1.03** | 2.04 | Increase | 3.16 | 0.00501616 | 0.1900 | similar to 60S ribosomal protein L29 (P23) (predicted) | PREDICTED: Rattus norvegicus similar to 60S ribosomal protein L29 (P23) (predicted) (RGD1562489_predicted), mRNA [XM_220370] | XM_220370 |
| **1.06** | 2.09 | Increase | 3.15 | 0.00511205 | 0.1909 | similar to RIKEN cDNA 2610003J06 | Rattus norvegicus similar to RIKEN cDNA 2610003J06 (RGD1307381), mRNA [NM_001014116] | NM_001014116 |
| **1.54** | 2.91 | Increase | 3.15 | 0.00517716 | 0.1914 | myosin, light polypeptide 2 | AGENCOURT_28626027 NIH_MGC_250 Rattus norvegicus cDNA clone IMAGE:7384149 5', mRNA sequence [CO563307] | CO563307 |
| **1.16** | 2.23 | Increase | 3.14 | 0.00528347 | 0.1933 | similar to Heparan-sulfate 6-O-sulfotransferase 2 (HS6ST-2) | PREDICTED: Rattus norvegicus similar to heparan sulfate 6-O-sulfotransferase 2 isoform S (predicted) | XM_228681 |

| | | | | | | | | |
|---|---|---|---|---|---|---|---|---|
| | | | | | | | (RGD1564397_predicted), mRNA [XM_228681] | |
| **-3.80** | 13.90 | Decrease | -3.14 | 0.00528409 | 0.1933 | cell death-inducing DNA fragmentation factor, alpha subunit-like effector A (predicted) | PREDICTED: Rattus norvegicus cell death-inducing DNA fragmentation factor, alpha subunit-like effector A (predicted) (Cidea_predicted), mRNA [XM_214551] | XM_214551 |
| **1.12** | 2.17 | Increase | 3.14 | 0.00530708 | 0.1937 | transmembrane protein 53 (predicted) | PREDICTED: Rattus norvegicus transmembrane protein 53 (predicted) (Tmem53_predicted), mRNA [XM_233431] | XM_233431 |
| **1.35** | 2.55 | Increase | 3.13 | 0.00544723 | 0.1960 | similar to scaffolding protein SLIPR (predicted) | Rattus norvegicus similar to scaffolding protein SLIPR (predicted) (RGD1306155_predicted), mRNA [NM_001037218] | NM_001037218 |
| **-1.18** | 2.27 | Decrease | -3.12 | 0.00554888 | 0.1970 | ENSRNOT00000051958 | Q9NQW1 (Q9NQW1) Secretory pathway component Sec31B-1, partial (10%) [TC548694] | |
| **-2.24** | 4.72 | Decrease | -3.11 | 0.00559753 | 0.1976 | titin | PREDICTED: Rattus norvegicus titin (Ttn), mRNA [XM_001065955] | XM_001065955 |
| **-1.24** | 2.36 | Decrease | -3.11 | 0.00567896 | 0.1982 | solute carrier family 30 (zinc transporter), member 4 | Rattus norvegicus solute carrier family 30 (zinc transporter), member 4 (Slc30a4), mRNA [NM_172066] | NM_172066 |
| **1.23** | 2.35 | Increase | 3.10 | 0.00573878 | 0.1983 | olfactory receptor 283 (predicted) | Rattus norvegicus olfactory receptor 283 (predicted) (Olr283_predicted), mRNA [NM_001001010] | NM_001001010 |
| **-1.34** | 2.54 | Decrease | -3.10 | 0.00581127 | 0.1989 | lipin 1 | Rattus norvegicus lipin 1 (Lpin1), mRNA [NM_001012111] | NM_001012111 |
| **1.33** | 2.52 | Increase | 3.09 | 0.00587023 | 0.1998 | follistatin | Rattus norvegicus follistatin (Fst), mRNA [NM_012561] | NM_012561 |
| **1.48** | 2.78 | Increase | 3.08 | 0.00602683 | 0.2011 | spastic paraplegia 21 homolog (human) | Rattus norvegicus spastic paraplegia 21 homolog (human) (Spg21), mRNA [NM_001006987] | NM_001006987 |

| | | | | | | | | |
|---|---|---|---|---|---|---|---|---|
| **1.49** | 2.81 | Increase | 3.08 | 0.0060366 | 0.2012 | carbohydrate sulfotransferase 12 | Rattus norvegicus carbohydrate sulfotransferase 12 (Chst12), mRNA [NM_001037775] | NM_001037775 |
| **-1.48** | 2.79 | Decrease | -3.08 | 0.00604993 | 0.2012 | HCR (a-helix coiled-coil rod homolog) | Rattus norvegicus HCR (a-helix coiled-coil rod homolog) (Hcr), mRNA [NM_001002822] | NM_001002822 |
| **-1.51** | 2.85 | Decrease | -3.08 | 0.00605562 | 0.2012 | similar to Dynamin-binding protein (Scaffold protein Tuba) | PREDICTED: Rattus norvegicus similar to Dynamin-binding protein (Scaffold protein Tuba) (LOC309362), mRNA [XM_219860] | XM_219860 |
| **2.37** | 5.16 | Increase | 3.08 | 0.00606736 | 0.2012 | similar to RIKEN cDNA 6330406I15 (predicted) | PREDICTED: Rattus norvegicus similar to RIKEN cDNA 6330406I15 (predicted) (RGD1307396_predicted), mRNA [XM_341029] | XM_341029 |
| **-1.09** | 2.13 | Decrease | -3.08 | 0.00610332 | 0.2019 | similar to high mobility group protein homolog HMG4 (predicted) | PREDICTED: Rattus norvegicus similar to high mobility group protein homolog HMG4 (predicted) (RGD1564407_predicted), mRNA [XM_223440] | XM_223440 |
| **-2.14** | 4.41 | Decrease | -3.07 | 0.00613449 | 0.2024 | AA892994 | AA892994 EST196797 Normalized rat kidney, Bento Soares Rattus sp. cDNA clone RKIBA84 3' end, mRNA sequence [AA892994] | AA892994 |
| **-1.07** | 2.10 | Decrease | -3.07 | 0.00621519 | 0.2030 | chromosome segregation 1-like (S. cerevisiae) (predicted) | PREDICTED: Rattus norvegicus chromosome segregation 1-like (S. cerevisiae) (predicted) (Cse1l_predicted), mRNA [XM_342581] | XM_342581 |
| **-1.40** | 2.64 | Decrease | -3.07 | 0.00621729 | 0.2030 | Ttn | PREDICTED: Rattus norvegicus titin (Ttn), mRNA [XM_001065955] | XM_001065955 |
| **-2.17** | 4.49 | Decrease | -3.06 | 0.00630278 | 0.2045 | X52757 | R.norvegicus mitochondrial D-loop region. [X52757] | X52757 |

| | | | | | | | | |
|---|---|---|---|---|---|---|---|---|
| **1.38** | 2.60 | Increase | 3.06 | 0.00637428 | 0.2050 | cofilin 1, non-muscle | Rattus norvegicus cofilin 1, non-muscle (Cfl1), mRNA [NM_017147] | NM_017147 |
| **-1.17** | 2.25 | Decrease | -3.05 | 0.00644075 | 0.2055 | programmed cell death 6 interacting protein | PREDICTED: Rattus norvegicus programmed cell death 6 interacting protein (Pdcd6ip), mRNA [XM_001076624] | XM_001076624 |
| **1.75** | 3.36 | Increase | 3.05 | 0.0065314 | 0.2070 | BF564217 | BF564217 UI-R-C4-alk-b-02-0-UI.r1 UI-R-C4 Rattus norvegicus cDNA clone UI-R-C4-alk-b-02-0-UI 5', mRNA sequence [BF564217] | BF564217 |
| **2.11** | 4.32 | Increase | 3.04 | 0.00662729 | 0.2085 | BM392202 | BM392202 UI-R-DO1-ckp-o-12-0-UI.s1 UI-R-DO1 Rattus norvegicus cDNA clone UI-R-DO1-ckp-o-12-0-UI 3', mRNA sequence [BM392202] | BM392202 |
| **1.09** | 2.13 | Increase | 3.04 | 0.00664561 | 0.2087 | pituitary tumor-transforming 1 | Rattus norvegicus pituitary tumor-transforming 1 (Pttg1), mRNA [NM_022391] | NM_022391 |
| **1.98** | 3.94 | Increase | 3.03 | 0.00669231 | 0.2094 | follistatin | Rattus norvegicus follistatin (Fst), mRNA [NM_012561] | NM_012561 |
| **1.78** | 3.44 | Increase | 3.03 | 0.00674725 | 0.2101 | growth arrest specific 7 | Rattus norvegicus growth arrest specific 7 (Gas7), mRNA [NM_053484] | NM_053484 |
| **-4.10** | 17.18 | Decrease | -3.03 | 0.00682525 | 0.2113 | similar to RIKEN cDNA C530028O21 gene | Rattus norvegicus similar to RIKEN cDNA C530028O21 gene (RGD1304952), mRNA [NM_001014059] | NM_001014059 |
| **1.09** | 2.13 | Increase | 3.02 | 0.00691209 | 0.2120 | similar to KIAA0974 protein | Rattus norvegicus similar to KIAA0974 protein (RGD1359592), mRNA [NM_001013878] | NM_001013878 |
| **2.83** | 7.13 | Increase | 3.02 | 0.00696461 | 0.2121 | AA944569 | AA944569 EST200068 Normalized rat embryo, Bento Soares Rattus sp. cDNA clone REMAL22 3' end, mRNA sequence [AA944569] | AA944569 |

| | | | | | | | | |
|---|---|---|---|---|---|---|---|---|
| **-2.12** | 4.33 | Decrease | -3.01 | 0.00703652 | 0.2134 | AI233993 | AI233993 EST230681 Normalized rat lung, Bento Soares Rattus sp. cDNA clone RLUCT82 3' end, mRNA sequence [AI233993] | AI233993 |
| **1.04** | 2.05 | Increase | 3.01 | 0.00704273 | 0.2135 | bcl2-associated death promoter | Rattus norvegicus bcl2-associated death promoter (Bad), mRNA [NM_022698] | NM_022698 |
| **1.52** | 2.87 | Increase | 3.00 | 0.00729822 | 0.2171 | AW917664 | AW917664 EST348968 Rat gene index, normalized rat, norvegicus, Bento Soares Rattus norvegicus cDNA clone RGIEG58 5' end, mRNA sequence [AW917664] | AW917664 |
| **-1.18** | 2.26 | Decrease | -2.99 | 0.00731168 | 0.2171 | ring finger protein 135 | Rattus norvegicus ring finger protein 135 (Rnf135), mRNA [NM_001012010] | NM_001012010 |
| **-1.22** | 2.33 | Decrease | -2.98 | 0.00746977 | 0.2188 | TC530939 | Q9ERK2 (Q9ERK2) Neprilysin-like peptidase gamma, partial (4%) [TC530939] | |
| **1.19** | 2.28 | Increase | 2.97 | 0.00767044 | 0.2210 | XM_342889 | Rattus norvegicus similar to hypothetical protein (LOC362570), mRNA [XM_342889] | XM_342889 |
| **1.13** | 2.18 | Increase | 2.96 | 0.00791288 | 0.2246 | reversion induced LIM gene | Rattus norvegicus reversion induced LIM gene (Ril), mRNA [NM_017062] | NM_017062 |
| **1.25** | 2.38 | Increase | 2.96 | 0.00795908 | 0.2256 | nicotinamide N-methyltransferase (predicted) | PREDICTED: Rattus norvegicus nicotinamide N-methyltransferase (predicted) (Nnmt_predicted), mRNA [XM_217138] | XM_217138 |
| **1.00** | 2.00 | Increase | 2.95 | 0.00811882 | 0.2268 | DnaJ (Hsp40) homolog, subfamily C, member 17 (predicted) | PREDICTED: Rattus norvegicus DnaJ (Hsp40) homolog, subfamily C, member 17 (predicted) (Dnajc17_predicted), mRNA [XM_230468] | XM_230468 |
| **2.10** | 4.29 | Increase | 2.95 | 0.00812464 | 0.2268 | Senp5_predicted | PREDICTED: Rattus norvegicus SUMO/sentrin specific protease 5 | XM_221369 |

| | | | | | | | | |
|---|---|---|---|---|---|---|---|---|
| | | | | | | | (predicted) (Senp5_predicted), mRNA [XM_221369] | |
| **1.12** | 2.18 | Increase | 2.94 | 0.00817349 | 0.2270 | AW523375 | AW523375 UI-R-BO0-aif-e-07-0-UI.s1 UI-R-BO0 Rattus norvegicus cDNA clone UI-R-BO0-aif-e-07-0-UI 3', mRNA sequence [AW523375] | AW523375 |
| **1.12** | 2.17 | Increase | 2.94 | 0.0082098 | 0.2275 | DiGeorge syndrome critical region gene 14 homolog (human) | Rattus norvegicus DiGeorge syndrome critical region gene 14 homolog (human) (Dgcr14), mRNA [NM_001012472] | NM_001012472 |
| **-1.01** | 2.02 | Decrease | -2.94 | 0.00827712 | 0.2284 | XM_225436 | Rattus norvegicus similar to hypothetical protein MGC39558 (LOC291212), mRNA [XM_225436] | XM_225436 |
| **-1.01** | 2.02 | Decrease | -2.94 | 0.00829809 | 0.2284 | similar to 5-aminoimidazole-4-carboxamide ribonucleotide formyltransferase/IMP cyclohydrolase (predicted) | PREDICTED: Rattus norvegicus similar to 5-aminoimidazole-4-carboxamide ribonucleotide formyltransferase/IMP cyclohydrolase (predicted) (RGD1559750_predicted), mRNA [XM_227632] | XM_227632 |
| **1.35** | 2.55 | Increase | 2.94 | 0.00831477 | 0.2284 | glutathione peroxidase 3 | Rattus norvegicus glutathione peroxidase 3 (Gpx3), mRNA [NM_022525] | NM_022525 |
| **1.31** | 2.48 | Increase | 2.94 | 0.00833605 | 0.2284 | AI145976 | AI145976 UI-R-BT0-qc-g-05-0-UI.s1 UI-R-BT0 Rattus norvegicus cDNA clone UI-R-BT0-qc-g-05-0-UI 3', mRNA sequence [AI145976] | AI145976 |
| **1.25** | 2.38 | Increase | 2.93 | 0.00837806 | 0.2291 | similar to RIKEN cDNA 1500015O10 (predicted) | PREDICTED: Rattus norvegicus similar to RIKEN cDNA 1500015O10 (predicted) (RGD1305645_predicted), mRNA [XM_343562] | XM_343562 |
| **-1.66** | 3.16 | Decrease | -2.93 | 0.00842478 | 0.2297 | Ttn | PREDICTED: Rattus norvegicus titin (Ttn), mRNA [XM_001065955] | XM_001065955 |

| | | | | | | | | |
|---|---|---|---|---|---|---|---|---|
| **-1.34** | 2.53 | Decrease | -2.92 | 0.00854064 | 0.2312 | splicing factor 3b, subunit 1 | BF523561 UI-R-C0-jr-d-05-0-UI.r1 UI-R-C0 Rattus norvegicus cDNA clone UI-R-C0-jr-d-05-0-UI 5', mRNA sequence [BF523561] | BF523561 |
| **-2.82** | 7.05 | Decrease | -2.92 | 0.00856814 | 0.2316 | U08214 | RSU08214 Rattus sp. DNA binding protein (URE-B1) mRNA, complete cds [U08214] | U08214 |
| **-1.15** | 2.22 | Decrease | -2.92 | 0.00861781 | 0.2321 | nebulin (predicted) | PREDICTED: Rattus norvegicus nebulin (predicted) (Neb_predicted), mRNA [XM_229925] | XM_229925 |
| **1.85** | 3.60 | Increase | 2.92 | 0.00870115 | 0.2326 | similar to guanine nucleotide binding protein, alpha stimulating, olfactory type | PREDICTED: Rattus norvegicus similar to guanine nucleotide binding protein, alpha stimulating, olfactory type (LOC682950), mRNA [XM_001060921] | XM_001060921 |
| **1.71** | 3.26 | Increase | 2.91 | 0.00874872 | 0.2331 | chymase 1, mast cell | Rattus norvegicus chymase 1, mast cell (Cma1), mRNA [NM_013092] | NM_013092 |
| **1.08** | 2.12 | Increase | 2.91 | 0.00880843 | 0.2343 | STARD3 N-terminal like | Rattus norvegicus STARD3 N-terminal like (Stard3nl), mRNA [NM_001008298] | NM_001008298 |
| **1.07** | 2.10 | Increase | 2.91 | 0.00882726 | 0.2345 | nuclear transcription factor-Y gamma | Rattus norvegicus nuclear transcription factor-Y gamma (Nfyc), mRNA [NM_012866] | NM_012866 |
| **1.95** | 3.86 | Increase | 2.91 | 0.00885347 | 0.2349 | similar to Antxr2 protein | PREDICTED: Rattus norvegicus similar to Antxr2 protein (LOC305633), mRNA [XM_223745] | XM_223745 |
| **-1.76** | 3.39 | Decrease | -2.90 | 0.00898534 | 0.2371 | AW141373 | AW141373 EST291414 Normalized rat brain, Bento Soares Rattus sp. cDNA clone RGIBP89 5' end similar to neural cell adhesion molecule NCAM-120, mRNA sequence [AW141373] | AW141373 |
| **1.77** | 3.41 | Increase | 2.88 | 0.00952512 | 0.2448 | histone cluster 1, H2bm (predicted) | PREDICTED: Rattus norvegicus histone 1, H2bm (predicted) | XM_341530 |

| | | | | | | | | |
|---|---|---|---|---|---|---|---|---|
| | | | | | | | (Hist1h2bm_predicted), mRNA [XM_341530] | |
| **-1.55** | 2.92 | Decrease | -2.88 | 0.00952634 | 0.2448 | titin | PREDICTED: Rattus norvegicus titin (Ttn), mRNA [XM_001065955] | XM_001065955 |
| **1.05** | 2.07 | Increase | 2.88 | 0.0095292 | 0.2448 | TPA regulated locus | Rattus norvegicus TPA regulated locus (Tparl), mRNA [NM_001024802] | NM_001024802 |
| **-2.96** | 7.80 | Decrease | -2.87 | 0.0096271 | 0.2454 | XM_239603 | Rattus norvegicus similar to RIKEN cDNA 1300006M19 (LOC304282), mRNA [XM_239603] | XM_239603 |
| **1.66** | 3.17 | Increase | 2.86 | 0.00984004 | 0.2471 | Uap1_predicted | PREDICTED: Rattus norvegicus UDP-N-acetylglucosamine pyrophosphorylase 1 (predicted) (Uap1_predicted), mRNA [XM_222863] | XM_222863 |
| **1.55** | 2.94 | Increase | 2.86 | 0.00990568 | 0.2482 | zinc finger protein 579 (predicted) | PREDICTED: Rattus norvegicus zinc finger protein 579 (predicted) (Znf579_predicted), mRNA [XM_218200] | XM_218200 |
| **2.23** | 4.69 | Increase | 2.86 | 0.0099432 | 0.2486 | leucine rich repeat containing 30 (predicted) | PREDICTED: Rattus norvegicus leucine rich repeat containing 30 (predicted) (Lrrc30_predicted), mRNA [XM_237538] | XM_237538 |
| **-1.46** | 2.76 | Decrease | -2.85 | 0.00996867 | 0.2486 | netrin 2-like (chicken) | PREDICTED: Rattus norvegicus netrin 2-like (chicken) (Ntn2l), mRNA [XM_343867] | XM_343867 |
| **1.23** | 2.35 | Increase | 2.85 | 0.01001135 | 0.2486 | procollagen, type VI, alpha 2 | PREDICTED: Rattus norvegicus procollagen, type VI, alpha 2 (Col6a2), mRNA [XM_342115] | XM_342115 |
| **-1.37** | 2.59 | Decrease | -2.85 | 0.01011066 | 0.2493 | RGD1565416_predicted | PREDICTED: Rattus norvegicus similar to talin 2 (predicted) (RGD1565416_predicted), mRNA [XM_236367] | XM_236367 |

| | | | | | | | | |
|---|---|---|---|---|---|---|---|---|
| **2.35** | 5.10 | Increase | 2.84 | 0.01022892 | 0.2505 | TC536217 | Q800A3 (Q800A3) Ddx49-A-prov protein (Fragment), partial (23%) [TC536217] | |
| **-1.27** | 2.40 | Decrease | -2.84 | 0.01026295 | 0.2510 | acyl-Coenzyme A dehydrogenase family, member 11 (predicted) | PREDICTED: Rattus norvegicus acyl-Coenzyme A dehydrogenase family, member 11 (predicted) (Acad11_predicted), mRNA [XM_236582] | XM_236582 |
| **-1.03** | 2.04 | Decrease | -2.84 | 0.01034504 | 0.2519 | hect (homologous to the E6-AP (UBE3A) carboxyl terminus) domain and RCC1 (CHC1)-like domain (RLD) 2 (predicted) | PREDICTED: Rattus norvegicus hect (homologous to the E6-AP (UBE3A) carboxyl terminus) domain and RCC1 (CHC1)-like domain (RLD) 2 (predicted) (Herc2_predicted), mRNA [XM_218720] | XM_218720 |
| **-1.14** | 2.21 | Decrease | -2.84 | 0.01036428 | 0.2521 | zinc finger and BTB domain containing 25 | Rattus norvegicus zinc finger and BTB domain containing 25 (Zbtb25), mRNA [NM_199496] | NM_199496 |
| **-1.59** | 3.00 | Decrease | -2.83 | 0.01046023 | 0.2536 | doublesex and mab-3 related transcription factor like family A2 (predicted) | PREDICTED: Rattus norvegicus doublesex and mab-3 related transcription factor like family A2 (predicted) (Dmrta2_predicted), mRNA [XM_233363] | XM_233363 |
| **1.26** | 2.40 | Increase | 2.82 | 0.01067749 | 0.2553 | similar to protein phosphatase 1, regulatory (inhibitor) subunit 1C (predicted) | BG670819 DRNBGB03 Rat DRG Library Rattus norvegicus cDNA clone DRNBGB03 5', mRNA sequence [BG670819] | BG670819 |
| **1.83** | 3.56 | Increase | 2.82 | 0.01074371 | 0.2554 | BF407382 | BF407382 UI-R-BJ2-bqd-d-06-0-UI.s1 UI-R-BJ2 Rattus norvegicus cDNA clone UI-R-BJ2-bqd-d-06-0-UI 3', mRNA sequence [BF407382] | BF407382 |
| **1.54** | 2.90 | Increase | 2.81 | 0.01088406 | 0.2577 | angiotensin II receptor, type 1 (AT1A) | Rattus norvegicus angiotensin II receptor, type 1 (AT1A) (Agtr1a), mRNA [NM_030985] | NM_030985 |

| | | | | | | | | |
|---|---|---|---|---|---|---|---|---|
| **1.62** | 3.07 | Increase | 2.81 | 0.01092323 | 0.2579 | death effector domain-containing | Rattus norvegicus death effector domain-containing (Dedd), mRNA [NM_031800] | NM_031800 |
| **-1.32** | 2.49 | Decrease | -2.81 | 0.01095405 | 0.2583 | USP6 N-terminal like (predicted) | Rattus norvegicus similar to Expressed sequence AI316785 (LOC291309), mRNA [XM_214508] | XM_214508 |
| **1.01** | 2.01 | Increase | 2.81 | 0.01097683 | 0.2587 | transmembrane protein 87A (predicted) | PREDICTED: Rattus norvegicus transmembrane protein 87A (predicted) (Tmem87a_predicted), mRNA [XM_345422] | XM_345422 |
| **-1.41** | 2.65 | Decrease | -2.81 | 0.01098555 | 0.2587 | diamine oxidase-like protein 1 | Rattus norvegicus diamine oxidase-like protein 1 (Doxl1), mRNA [NM_199233] | NM_199233 |
| **-2.37** | 5.16 | Decrease | -2.81 | 0.01108028 | 0.2595 | similar to HTGN29 protein; keratinocytes associated transmembrane protein 2 | PREDICTED: Rattus norvegicus similar to HTGN29 protein; keratinocytes associated transmembrane protein 2 (RGD1310352), mRNA [XM_220404] | XM_220404 |
| **-1.31** | 2.48 | Decrease | -2.81 | 0.01108456 | 0.2595 | similar to RNA binding motif, single stranded interacting protein 3 isoform 1 | PREDICTED: Rattus norvegicus similar to RNA binding motif, single stranded interacting protein 3 isoform 1, transcript variant 5 (LOC680726), mRNA [XM_001061391] | XM_001061391 |
| **-1.01** | 2.02 | Decrease | -2.80 | 0.01122104 | 0.2606 | phospholipase C, beta 4 | Rattus norvegicus phospholipase C, beta 4 (Plcb4), mRNA [NM_024353] | NM_024353 |
| **-1.24** | 2.36 | Decrease | -2.80 | 0.01127263 | 0.2612 | similar to RIKEN cDNA 1700081O22 | Rattus norvegicus similar to RIKEN cDNA 1700081O22 (LOC367515), mRNA [NM_001014271] | NM_001014271 |
| **-1.04** | 2.05 | Decrease | -2.79 | 0.01151578 | 0.2641 | ryanodine receptor 1, skeletal muscle | PREDICTED: Rattus norvegicus ryanodine receptor 1, skeletal muscle (Ryr1), mRNA [XM_341818] | XM_341818 |

| | | | | | | | | |
|---|---|---|---|---|---|---|---|---|
| **-1.89** | 3.71 | Decrease | -2.79 | 0.01161151 | 0.2644 | M33313 | RATCYP2A21 Rat hepatic steroid hydroxylase IIA2 (CYP2A2) gene, exons 1 and 2 [M33313] | M33313 |
| **1.08** | 2.11 | Increase | 2.78 | 0.01166414 | 0.2647 | serine/threonine kinase 16 | Rattus norvegicus serine/threonine kinase 16 (Stk16), mRNA [NM_173142] | NM_173142 |
| **1.55** | 2.92 | Increase | 2.78 | 0.01186883 | 0.2667 | choline/ethanolamine phosphotransferase 1 | Rattus norvegicus choline/ethanolamine phosphotransferase 1 (Cept1), mRNA [NM_001007699] | NM_001007699 |
| **-1.18** | 2.27 | Decrease | -2.78 | 0.01186922 | 0.2667 | ENSRNOT00000027501 | Rattus norvegicus LRRGT00043 mRNA, complete cds. [AY383698] | AY383698 |
| **1.70** | 3.25 | Increase | 2.77 | 0.01200626 | 0.2667 | Ac1258 | Rattus norvegicus Ac1258 mRNA, complete cds. [AY310151] | AY310151 |
| **-2.84** | 7.18 | Decrease | -2.77 | 0.01203121 | 0.2668 | Ttn | PREDICTED: Rattus norvegicus titin (Ttn), mRNA [XM_001065955] | XM_001065955 |
| **1.27** | 2.42 | Increase | 2.76 | 0.01222808 | 0.2689 | gap junction membrane channel protein alpha 7 | PREDICTED: Rattus norvegicus gap junction membrane channel protein alpha 7 (Gja7), mRNA [XM_001081521] | XM_001081521 |
| **-4.65** | 25.02 | Decrease | -2.76 | 0.01223386 | 0.2689 | BF550568 | BF550568 UI-R-C0-jl-b-08-0-UI.r1 UI-R-C0 Rattus norvegicus cDNA clone UI-R-C0-jl-b-08-0-UI 5', mRNA sequence [BF550568] | BF550568 |
| **1.06** | 2.09 | Increase | 2.76 | 0.01228861 | 0.2692 | Josephin domain containing 2 (predicted) | PREDICTED: Rattus norvegicus Josephin domain containing 2 (predicted) (Josd2_predicted), mRNA [XM_214929] | XM_214929 |
| **1.27** | 2.41 | Increase | 2.75 | 0.01260622 | 0.2731 | TC521066 | Q49846 (Q49846) B2235_C3_243 (Possible conserved membrane protein), partial (7%) [TC521066] | |
| **1.54** | 2.91 | Increase | 2.75 | 0.0126117 | 0.2731 | non-metastatic cells 7, protein expressed in | Rattus norvegicus non-metastatic cells 7, protein expressed in (Nme7), mRNA [NM_138532] | NM_138532 |

| | | | | | | | | |
|---|---|---|---|---|---|---|---|---|
| **-1.48** | 2.79 | Decrease | -2.75 | 0.01264704 | 0.2733 | ubiquitin specific peptidase 9, X chromosome (predicted) | PREDICTED: Rattus norvegicus ubiquitin specific peptidase 9, X chromosome (predicted) (Usp9x_predicted), mRNA [XM_343766] | XM_343766 |
| **1.04** | 2.06 | Increase | 2.74 | 0.01292742 | 0.2768 | BI301310 | UI-R-DL0-cio-f-15-0-UI.s1 UI-R-DL0 Rattus norvegicus cDNA clone UI-R-DL0-cio-f-15-0-UI 3', mRNA sequence [BI301310] | BI301310 |
| **-2.30** | 4.91 | Decrease | -2.73 | 0.0130034 | 0.2773 | MAM domain containing 4 | Rattus norvegicus MAM domain containing 4 (Mamdc4), mRNA [NM_145768] | NM_145768 |
| **-2.07** | 4.20 | Decrease | -2.73 | 0.0131755 | 0.2786 | myosin Va | Rattus norvegicus myosin Va (Myo5a), mRNA [NM_022178] | NM_022178 |
| **1.00** | 2.00 | Increase | 2.73 | 0.01320504 | 0.2787 | procollagen, type 1, alpha 1 | PREDICTED: Rattus norvegicus procollagen, type 1, alpha 1 (Col1a1), mRNA [XM_213440] | XM_213440 |
| **1.30** | 2.46 | Increase | 2.72 | 0.01325323 | 0.2790 | LOC683544 | PREDICTED: Rattus norvegicus similar to proline rich membrane anchor 1 (LOC683544), mRNA [XM_001066450] | XM_001066450 |
| **1.07** | 2.10 | Increase | 2.72 | 0.0133098 | 0.2796 | similar to RIKEN cDNA 5830446M03 | Rattus norvegicus similar to RIKEN cDNA 5830446M03 (RGD1307688), mRNA [NM_001014188] | NM_001014188 |
| **1.23** | 2.34 | Increase | 2.71 | 0.01366456 | 0.2832 | tripartite motif-containing 42 | Rattus norvegicus tripartite motif-containing 42 (Trim42), mRNA [NM_001013955] | NM_001013955 |
| **-1.19** | 2.29 | Decrease | -2.71 | 0.01375115 | 0.2837 | nischarin | PREDICTED: Rattus norvegicus nischarin (Nisch), mRNA [XM_240330] | XM_240330 |
| **-1.28** | 2.42 | Decrease | -2.70 | 0.01386863 | 0.2849 | BF408265 | BF408265 UI-R-BJ2-bqr-c-10-0-UI.s1 UI-R-BJ2 Rattus norvegicus cDNA clone UI-R-BJ2-bqr-c-10-0-UI 3', mRNA sequence [BF408265] | BF408265 |

| | | | | | | | | |
|---|---|---|---|---|---|---|---|---|
| **1.08** | 2.12 | Increase | 2.70 | 0.01390994 | 0.2850 | similar to exosome component 1 | PREDICTED: Rattus norvegicus similar to exosome component 1 (LOC679140), mRNA [XM_001054866] | XM_001054866 |
| **1.25** | 2.37 | Increase | 2.70 | 0.01392796 | 0.2850 | SRY-box containing gene 4 (predicted) | PREDICTED: Rattus norvegicus SRY-box containing gene 4 (predicted) (Sox4_predicted), mRNA [XM_344594] | XM_344594 |
| **-1.44** | 2.71 | Decrease | -2.70 | 0.01393741 | 0.2850 | microtubule associated serine/threonine kinase 2 (predicted) | PREDICTED: Rattus norvegicus microtubule associated serine/threonine kinase 2 (predicted) (Mast2_predicted), mRNA [XM_233782] | XM_233782 |
| **1.91** | 3.75 | Increase | 2.70 | 0.01394159 | 0.2850 | BF393048 | BF393048 UI-R-CA0-bgp-e-04-0-UI.s1 UI-R-CA0 Rattus norvegicus cDNA clone UI-R-CA0-bgp-e-04-0-UI 3', mRNA sequence [BF393048] | BF393048 |
| **1.23** | 2.34 | Increase | 2.70 | 0.01402709 | 0.2855 | BI277534 | UI-R-CY0-bxs-d-02-0-UI.s1 UI-R-CY0 Rattus norvegicus cDNA clone UI-R-CY0-bxs-d-02-0-UI 3', mRNA sequence [BI277534] | BI277534 |
| **-1.06** | 2.09 | Decrease | -2.70 | 0.01408667 | 0.2859 | AW144275 | AW144275 EST294571 Normalized rat ovary, Bento Soares Rattus sp. cDNA clone RGICP06 5' end, mRNA sequence [AW144275] | AW144275 |
| **1.29** | 2.45 | Increase | 2.69 | 0.0141436 | 0.2864 | similar to G2 (predicted) | PREDICTED: Rattus norvegicus similar to G2 (predicted) (RGD1310754_predicted), mRNA [XM_001079537] | XM_001079537 |
| **1.26** | 2.40 | Increase | 2.69 | 0.01422858 | 0.2869 | XM_214487 | Rattus norvegicus similar to RIKEN cDNA 5730599O09 (LOC291188), mRNA [XM_214487] | XM_214487 |
| **2.07** | 4.20 | Increase | 2.69 | 0.01426916 | 0.2874 | artemin | Rattus norvegicus artemin (Artn), mRNA [NM_053397] | NM_053397 |

| | | | | | | | | |
|---|---|---|---|---|---|---|---|---|
| **1.93** | 3.81 | Increase | 2.68 | 0.01460522 | 0.2893 | brain-specific angiogenesis inhibitor 1-associated protein 2 | Rattus norvegicus brain-specific angiogenesis inhibitor 1-associated protein 2 (Baiap2), mRNA [NM_057196] | NM_057196 |
| **1.40** | 2.65 | Increase | 2.68 | 0.01466533 | 0.2894 | tumor necrosis factor (ligand) superfamily, member 13 | Rattus norvegicus tumor necrosis factor (ligand) superfamily, member 13 (Tnfsf13), mRNA [NM_001009623] | NM_001009623 |
| **-1.32** | 2.49 | Decrease | -2.68 | 0.01472376 | 0.2896 | AW918233 | AW918233 EST349537 Rat gene index, normalized rat, norvegicus, Bento Soares Rattus norvegicus cDNA clone RGIEO19 5' end, mRNA sequence [AW918233] | AW918233 |
| **-1.03** | 2.04 | Decrease | -2.67 | 0.01481576 | 0.2904 | AI236318 | EST232880 Normalized rat ovary, Bento Soares Rattus sp. cDNA clone ROVDE13 3' end, mRNA sequence [AI236318] | AI236318 |
| **1.08** | 2.12 | Increase | 2.67 | 0.01490865 | 0.2912 | Col11a1 | PREDICTED: Rattus norvegicus procollagen, type XI, alpha 1 (Col11a1), mRNA [XM_342325] | XM_342325 |
| **-1.18** | 2.26 | Decrease | -2.67 | 0.01505742 | 0.2923 | CO388689 | AGENCOURT_26625193 NIH_MGC_253 Rattus norvegicus cDNA clone IMAGE:7304073 5', mRNA sequence [CO388689] | CO388689 |
| **1.17** | 2.25 | Increase | 2.66 | 0.01519363 | 0.2936 | TAF7 RNA polymerase II, TATA box binding protein (TBP)-associated factor, 55kDa (predicted) | PREDICTED: Rattus norvegicus TAF7 RNA polymerase II, TATA box binding protein (TBP)-associated factor, 55kDa (predicted) (Taf7_predicted), mRNA [XM_226031] | XM_226031 |
| **1.24** | 2.36 | Increase | 2.66 | 0.01523302 | 0.2939 | sal-like 3 (Drosophila) (predicted) | PREDICTED: Rattus norvegicus sal-like 3 (Drosophila) (predicted) (Sall3_predicted), mRNA [XM_344708] | XM_344708 |

| | | | | | | | | |
|---|---|---|---|---|---|---|---|---|
| **-1.31** | 2.48 | Decrease | -2.66 | 0.01533722 | 0.2946 | Dst_predicted | PREDICTED: Rattus norvegicus dystonin (predicted) (Dst_predicted), mRNA [XM_237042] | XM_237042 |
| **1.07** | 2.09 | Increase | 2.65 | 0.01548465 | 0.2954 | protein phosphatase 1, regulatory (inhibitor) subunit 14A | Rattus norvegicus protein phosphatase 1, regulatory (inhibitor) subunit 14A (Ppp1r14a), mRNA [NM_130403] | NM_130403 |
| **-1.50** | 2.84 | Decrease | -2.65 | 0.01551369 | 0.2955 | AW920343 | AW920343 EST351647 Rat gene index, normalized rat, norvegicus, Bento Soares Rattus norvegicus cDNA clone RGIGU32 5' end, mRNA sequence [AW920343] | AW920343 |
| **1.05** | 2.07 | Increase | 2.65 | 0.01556498 | 0.2957 | similar to Tetraspanin-15 (Tspan-15) (Transmembrane 4 superfamily member 15) (Tetraspan NET-7) | PREDICTED: Rattus norvegicus similar to Tetraspanin-15 (Tspan-15) (Transmembrane 4 superfamily member 15) (Tetraspan NET-7) (LOC679462), mRNA [XM_001056482] | XM_001056482 |
| **1.83** | 3.56 | Increase | 2.65 | 0.01572027 | 0.2964 | LOC679596 | PREDICTED: Rattus norvegicus similar to GABA(A) receptor-associated protein like 2 (LOC679596), mRNA [XM_001053633] | XM_001053633 |
| **-2.07** | 4.21 | Decrease | -2.64 | 0.01577634 | 0.2968 | period homolog 2 (Drosophila) | Rattus norvegicus period homolog 2 (Drosophila) (Per2), mRNA [NM_031678] | NM_031678 |
| **-3.48** | 11.15 | Decrease | -2.64 | 0.01602261 | 0.2994 | AI008646 | AI008646 EST203097 Normalized rat embryo, Bento Soares Rattus sp. cDNA clone REMBB18 3' end, mRNA sequence [AI008646] | AI008646 |
| **-1.16** | 2.24 | Decrease | -2.64 | 0.01604015 | 0.2994 | similar to KIAA0853 protein (predicted) | PREDICTED: Rattus norvegicus similar to KIAA0853 protein (predicted) | XM_573801 |

| | | | | | | | | |
|---|---|---|---|---|---|---|---|---|
| | | | | | | | (RGD1563689_predicted), mRNA [XM_573801] | |
| **1.59** | 3.00 | Increase | 2.64 | 0.01609348 | 0.3001 | schlafen 2 (predicted) | PREDICTED: Rattus norvegicus schlafen 2 (predicted) (Slfn2_predicted), mRNA [XM_220779] | XM_220779 |
| **1.38** | 2.60 | Increase | 2.63 | 0.01628676 | 0.3022 | cathepsin K | Rattus norvegicus cathepsin K (Ctsk), mRNA [NM_031560] | NM_031560 |
| **1.21** | 2.31 | Increase | 2.63 | 0.01643612 | 0.3039 | drebrin 1 | Rattus norvegicus drebrin 1 (Dbn1), mRNA [NM_031024] | NM_031024 |
| **1.17** | 2.26 | Increase | 2.62 | 0.0165623 | 0.3047 | v-ral simian leukemia viral oncogene homolog B | Rattus norvegicus v-ral simian leukemia viral oncogene homolog B (Ralb), mRNA [NM_053821] | NM_053821 |
| **1.04** | 2.06 | Increase | 2.62 | 0.01657829 | 0.3049 | TC562057 | Q8R0F4 (Q8R0F4) BC026978 protein (Fragment), partial (20%) [TC562057] | |
| **-1.82** | 3.53 | Decrease | -2.62 | 0.01659621 | 0.3051 | protein kinase, cGMP-dependent, type 1 (mapped) | PREDICTED: Rattus norvegicus protein kinase, cGMP-dependent, type 1 (mapped) (Prkg1_mapped), mRNA [XM_219805] | XM_219805 |
| **-1.28** | 2.44 | Decrease | -2.61 | 0.01681984 | 0.3067 | supervillin (predicted) | PREDICTED: Rattus norvegicus supervillin (predicted) (Svil_predicted), mRNA [XM_341540] | XM_341540 |
| **-1.58** | 3.00 | Decrease | -2.61 | 0.01689749 | 0.3073 | similar to Myeloid/lymphoid or mixed-lineage leukemia protein 3 homolog (Histone-lysine N-methyltransferase, H3 lysine-4 specific MLL3) | PREDICTED: Rattus norvegicus similar to Myeloid/lymphoid or mixed-lineage leukemia protein 3 homolog (Histone-lysine N-methyltransferase, H3 lysine-4 specific MLL3) (LOC679252), mRNA [XM_001054661] | XM_001054661 |
| **-1.43** | 2.69 | Decrease | -2.61 | 0.01697028 | 0.3078 | transient receptor potential cation channel, subfamily C, member 1 | Rattus norvegicus transient receptor potential cation channel, subfamily C, member 1 (Trpc1), mRNA [NM_053558] | NM_053558 |

| | | | | | | | | |
|---|---|---|---|---|---|---|---|---|
| **1.50** | 2.83 | Increase | 2.61 | 0.01700255 | 0.3078 | thrombospondin 2 | PREDICTED: Rattus norvegicus thrombospondin 2 (Thbs2), mRNA [XM_214778] | XM_214778 |
| **1.10** | 2.14 | Increase | 2.61 | 0.01704674 | 0.3079 | TC541278 | Q8R4C3 (Q8R4C3) Csr1, partial (33%) [TC541278] | |
| **2.81** | 7.03 | Increase | 2.61 | 0.01707904 | 0.3081 | chondroadherin | Rattus norvegicus chondroadherin (Chad), mRNA [NM_019164] | NM_019164 |
| **-1.11** | 2.16 | Decrease | -2.60 | 0.01718253 | 0.3092 | olfactory receptor 157 (predicted) | Rattus norvegicus olfactory receptor 157 (predicted) (Olr157_predicted), mRNA [NM_001000169] | NM_001000169 |
| **1.68** | 3.21 | Increase | 2.60 | 0.01732919 | 0.3109 | AA955229 | AA955229 UI-R-A1-ej-f-11-0-UI.s1 UI-R-A1 Rattus norvegicus cDNA clone UI-R-A1-ej-f-11-0-UI 3' similar to gi [AA955229] | AA955229 |
| **-1.27** | 2.42 | Decrease | -2.60 | 0.01743481 | 0.3119 | LOC679811 | PREDICTED: Rattus norvegicus similar to RIKEN cDNA D930015E06, transcript variant 3 (LOC679811), mRNA [XM_001054550] | XM_001054550 |
| **1.04** | 2.05 | Increase | 2.60 | 0.01751446 | 0.3123 | XM_346216 | Rattus norvegicus similar to Itga6 protein (LOC367576), mRNA [XM_346216] | XM_346216 |
| **1.11** | 2.16 | Increase | 2.60 | 0.0175352 | 0.3124 | AW529005 | AW529005 UI-R-BT1-aka-a-11-0-UI.s1 UI-R-BT1 Rattus norvegicus cDNA clone UI-R-BT1-aka-a-11-0-UI 3', mRNA sequence [AW529005] | AW529005 |
| **1.33** | 2.52 | Increase | 2.59 | 0.01782956 | 0.3142 | similar to Ext1 | PREDICTED: Rattus norvegicus similar to Ext1 (LOC299907), mRNA [XM_216920] | XM_216920 |
| **1.58** | 2.99 | Increase | 2.58 | 0.01793143 | 0.3153 | AA850885 | AA850885 EST193653 Normalized rat ovary, Bento Soares Rattus sp. cDNA clone ROVAO01 3' end, mRNA sequence [AA850885] | AA850885 |
| **1.11** | 2.16 | Increase | 2.58 | 0.01830055 | 0.3178 | similar to ADP-ribosylation factor-like 10B (predicted) | PREDICTED: Rattus norvegicus similar to ADP-ribosylation factor- | XM_573456 |

| | | | | | | | | |
|---|---|---|---|---|---|---|---|---|
| | | | | | | | like 10B (predicted) (RGD1565940_predicted), mRNA [XM_573456] | |
| **-3.12** | 8.70 | Decrease | -2.57 | 0.01838832 | 0.3189 | AW144502 | AW144502 EST294879 Normalized rat embryo, Bento Soares Rattus sp. cDNA clone RGIAM10 5' end, mRNA sequence [AW144502] | AW144502 |
| **1.04** | 2.06 | Increase | 2.57 | 0.01841647 | 0.3191 | AW143671 | AW143671 EST293967 Normalized rat embryo, Bento Soares Rattus sp. cDNA clone RGIBX76 5' end, mRNA sequence [AW143671] | AW143671 |
| **1.24** | 2.37 | Increase | 2.57 | 0.01842896 | 0.3191 | promethin | Rattus norvegicus promethin (LOC378467), mRNA [NM_194354] | NM_194354 |
| **1.19** | 2.27 | Increase | 2.57 | 0.01843961 | 0.3191 | acyl-Coenzyme A binding domain containing 4 | Rattus norvegicus acyl-Coenzyme A binding domain containing 4 (Acbd4), mRNA [NM_001012013] | NM_001012013 |
| **1.35** | 2.54 | Increase | 2.57 | 0.01852726 | 0.3195 | hypothetical protein LOC499856 | Rattus norvegicus hypothetical protein LOC499856 (LOC499856), mRNA [NM_001025042] | NM_001025042 |
| **-1.04** | 2.05 | Decrease | -2.57 | 0.01859502 | 0.3198 | similar to RIKEN cDNA E130308A19 (predicted) | PREDICTED: Rattus norvegicus similar to RIKEN cDNA E130308A19 (predicted), transcript variant 2 (RGD1310951_predicted), mRNA [XM_001056694] | XM_001056694 |
| **2.98** | 7.87 | Increase | 2.57 | 0.01859884 | 0.3198 | TC535418 | Q6PD06 (Q6PD06) Cdc23 protein, partial (24%) [TC535418] | |
| **1.24** | 2.37 | Increase | 2.57 | 0.01863681 | 0.3202 | Usher syndrome 1C homolog (human) | Rattus norvegicus harmonin a1 (Ush1c) mRNA, complete cds; alternatively spliced [AY588414] | AY588414 |
| **1.36** | 2.58 | Increase | 2.57 | 0.01869699 | 0.3205 | CDC23 (cell division cycle 23, yeast, homolog) | Rattus norvegicus similar to cell division cycle protein 23; anaphase-promoting complex subunit 8 (LOC291689), mRNA [XM_214588] | XM_214588 |

| | | | | | | | | |
|---|---|---|---|---|---|---|---|---|
| **1.04** | 2.05 | Increase | 2.56 | 0.01900729 | 0.3233 | AA925266 | AA925266 UI-R-A1-ee-f-06-0-UI.s1 UI-R-A1 Rattus norvegicus cDNA clone UI-R-A1-ee-f-06-0-UI 3', mRNA sequence [AA925266] | AA925266 |
| **1.38** | 2.61 | Increase | 2.56 | 0.01903899 | 0.3233 | pyrimidinergic receptor P2Y, G-protein coupled, 6 | Rattus norvegicus pyrimidinergic receptor P2Y, G-protein coupled, 6 (P2ry6), mRNA [NM_057124] | NM_057124 |
| **1.52** | 2.86 | Increase | 2.56 | 0.01904764 | 0.3233 | cytochrome b5 reductase 3 | Rattus norvegicus cytochrome b5 reductase 3 (Cyb5r3), mRNA [NM_138877] | NM_138877 |
| **1.07** | 2.09 | Increase | 2.56 | 0.01907299 | 0.3233 | prepro-Neuropeptide W polypeptide | Rattus norvegicus prepro-Neuropeptide W polypeptide (LOC259224), mRNA [NM_153294] | NM_153294 |
| **1.45** | 2.74 | Increase | 2.56 | 0.01908543 | 0.3233 | LOC682841 | PREDICTED: Rattus norvegicus similar to H3 histone, family 3B (LOC682841), mRNA [XM_001063350] | XM_001063350 |
| **-1.13** | 2.19 | Decrease | -2.55 | 0.01923209 | 0.3237 | vacuolar protein sorting 13D (yeast) (predicted) | PREDICTED: Rattus norvegicus vacuolar protein sorting 13D (yeast) (predicted) (Vps13d_predicted), mRNA [XM_233792] | XM_233792 |
| **-1.16** | 2.23 | Decrease | -2.55 | 0.01929009 | 0.3238 | Bmpr2 | PREDICTED: Rattus norvegicus bone morphogenic protein receptor, type II (serine/threonine kinase) (Bmpr2), mRNA [XM_217409] | XM_217409 |
| **1.59** | 3.01 | Increase | 2.54 | 0.01957363 | 0.3250 | angiopoietin-like 4 | Rattus norvegicus angiopoietin-like 4 (Angptl4), mRNA [NM_199115] | NM_199115 |
| **1.26** | 2.39 | Increase | 2.54 | 0.01967334 | 0.3260 | hypothetical protein LOC691995 | PREDICTED: Rattus norvegicus hypothetical protein LOC691995 (LOC691995), mRNA [XM_001080750] | XM_001080750 |
| **1.71** | 3.27 | Increase | 2.54 | 0.01972914 | 0.3265 | prostaglandin E synthase | Rattus norvegicus prostaglandin E synthase (Ptges), mRNA [NM_021583] | NM_021583 |

| | | | | | | | | |
|---|---|---|---|---|---|---|---|---|
| **-1.93** | 3.82 | Decrease | -2.53 | 0.02010545 | 0.3290 | short form of beta II spectrin | RNO242018 Rattus norvegicus mRNA for short form of beta II spectrin, partial [AJ242018] | AJ242018 |
| **2.05** | 4.15 | Increase | 2.53 | 0.02019218 | 0.3295 | similar to hypothetical protein (predicted) | PREDICTED: Rattus norvegicus similar to hypothetical protein (predicted) (RGD1565522_predicted), mRNA [XM_237794] | XM_237794 |
| **-1.10** | 2.15 | Decrease | -2.53 | 0.02025678 | 0.3297 | LOC688211 | PREDICTED: Rattus norvegicus hypothetical protein LOC688211 (LOC688211), mRNA [XM_001081514] | XM_001081514 |
| **1.11** | 2.16 | Increase | 2.53 | 0.02036398 | 0.3302 | AI177494 | AI177494 EST221126 Normalized rat placenta, Bento Soares Rattus sp. cDNA clone RPLCA67 3' end, mRNA sequence [AI177494] | AI177494 |
| **1.57** | 2.97 | Increase | 2.52 | 0.02038753 | 0.3302 | XM_345436 | Rattus norvegicus similar to 40S RIBOSOMAL PROTEIN S3A (V-FOS TRANSFORMATION EFFECTOR PROTEIN) (LOC366193), mRNA [XM_345436] | XM_345436 |
| **1.85** | 3.60 | Increase | 2.52 | 0.02040549 | 0.3303 | BQ203347 | BQ203347 UI-R-DZ1-cnf-i-24-0-UI.s1 NCI_CGAP_DZ1 Rattus norvegicus cDNA clone IMAGE:7344962 3', mRNA sequence [BQ203347] | BQ203347 |
| **-1.45** | 2.74 | Decrease | -2.52 | 0.02042297 | 0.3303 | similar to RIKEN cDNA 2310045A20 (predicted) | PREDICTED: Rattus norvegicus similar to RIKEN cDNA 2310045A20 (predicted) (RGD1562860_predicted), mRNA [XM_341223] | XM_341223 |
| **1.25** | 2.37 | Increase | 2.52 | 0.02060741 | 0.3318 | MUS81 endonuclease homolog (yeast) | Rattus norvegicus MUS81 endonuclease homolog (yeast) (Mus81), mRNA [NM_001025645] | NM_001025645 |

| | | | | | | | | |
|---|---|---|---|---|---|---|---|---|
| **1.02** | 2.02 | Increase | 2.52 | 0.02069596 | 0.3328 | leukotriene C4 synthase | Rattus norvegicus leukotriene C4 synthase (Ltc4s), mRNA [NM_053639] | NM_053639 |
| **-1.25** | 2.39 | Decrease | -2.52 | 0.0207058 | 0.3328 | hect (homologous to the E6-AP (UBE3A) carboxyl terminus) domain and RCC1 (CHC1)-like domain (RLD) 1 (predicted) | PREDICTED: Rattus norvegicus hect (homologous to the E6-AP (UBE3A) carboxyl terminus) domain and RCC1 (CHC1)-like domain (RLD) 1 (predicted) (Herc1_predicted), mRNA [XM_236362] | XM_236362 |
| **-1.76** | 3.38 | Decrease | -2.52 | 0.02072519 | 0.3328 | BF413313 | UI-R-BT1-bnw-d-07-0-UI.s1 UI-R-BT1 Rattus norvegicus cDNA clone UI-R-BT1-bnw-d-07-0-UI 3', mRNA sequence [BF413313] | BF413313 |
| **-1.85** | 3.61 | Decrease | -2.51 | 0.02086794 | 0.3337 | peptidyl-tRNA hydrolase 1 homolog (S. cerevisiae) (predicted) | PREDICTED: Rattus norvegicus peptidyl-tRNA hydrolase 1 homolog (S. cerevisiae) (predicted) (Ptrh1_predicted), mRNA [XM_342416] | XM_342416 |
| **1.55** | 2.92 | Increase | 2.50 | 0.02128455 | 0.3377 | AI385133 | AI385133 UI-R-A0-ay-f-02-0-UI.s1 UI-R-A0 Rattus norvegicus cDNA clone UI-R-A0-ay-f-02-0-UI 3', mRNA sequence [AI385133] | AI385133 |
| **1.45** | 2.73 | Increase | 2.50 | 0.02155472 | 0.3389 | ENSRNOT00000052065 | PREDICTED: Rattus norvegicus similar to ribosomal protein S10 (LOC364977), mRNA [XM_344746] | XM_344746 |
| **-1.41** | 2.66 | Decrease | -2.50 | 0.02158464 | 0.3390 | Down syndrome critical region gene 1-like 1 | Rattus norvegicus Down syndrome critical region gene 1-like 1 (Dscr1l1), mRNA [NM_175578] | NM_175578 |
| **-1.23** | 2.34 | Decrease | -2.49 | 0.0217374 | 0.3397 | polymerase (DNA directed), alpha 2 | Rattus norvegicus polymerase (DNA directed), alpha 2 (Pola2), mRNA [NM_053480] | NM_053480 |
| **1.02** | 2.02 | Increase | 2.49 | 0.0217481 | 0.3397 | CD302 antigen | Rattus norvegicus CD302 antigen (Cd302), mRNA [NM_001013916] | NM_001013916 |

| | | | | | | | | |
|---|---|---|---|---|---|---|---|---|
| **1.63** | 3.10 | Increase | 2.49 | 0.02175304 | 0.3397 | CD44 antigen | Rattus norvegicus CD44 antigen (Cd44), mRNA [NM_012924] | NM_012924 |
| **1.07** | 2.10 | Increase | 2.49 | 0.02177022 | 0.3399 | RGD1561069_predicted | PREDICTED: Rattus norvegicus similar to F-box only protein 31 (predicted) (RGD1561069_predicted), mRNA [XM_574248] | XM_574248 |
| **-1.65** | 3.13 | Decrease | -2.49 | 0.0220242 | 0.3410 | abhydrolase domain containing 1 | Rattus norvegicus abhydrolase domain containing 1 (Abhd1), mRNA [NM_001008520] | NM_001008520 |
| **1.37** | 2.59 | Increase | 2.48 | 0.02231967 | 0.3427 | proline arginine-rich end leucine-rich repeat protein | Rattus norvegicus proline arginine-rich end leucine-rich repeat protein (Prelp), mRNA [NM_053385] | NM_053385 |
| **2.49** | 5.61 | Increase | 2.47 | 0.02269923 | 0.3454 | acetylcholinesterase | R.norvegicus mRNA for glycolipid-anchored form of acetylcholinesterase (HAChE). [X70140] | X70140 |
| **1.57** | 2.96 | Increase | 2.47 | 0.0229602 | 0.3475 | nuclear factor, interleukin 3 regulated | Rattus norvegicus nuclear factor, interleukin 3 regulated (Nfil3), mRNA [NM_053727] | NM_053727 |
| **1.28** | 2.43 | Increase | 2.47 | 0.02305132 | 0.3484 | AW535083 | AW535083 UI-R-C4-als-a-07-0-UI.s1 UI-R-C4 Rattus norvegicus cDNA clone UI-R-C4-als-a-07-0-UI 3', mRNA sequence [AW535083] | AW535083 |
| **1.28** | 2.43 | Increase | 2.47 | 0.02310719 | 0.3485 | AF216218 | AF216218 Rattus norvegicus orphanin FQ receptor gene (OFQR), complete cds, alternatively spliced [AF216218] | AF216218 |
| **1.11** | 2.15 | Increase | 2.46 | 0.0232104 | 0.3492 | lactamase, beta (predicted) | PREDICTED: Rattus norvegicus lactamase, beta (predicted) (Lactb_predicted), mRNA [XM_217181] | XM_217181 |
| **-1.21** | 2.32 | Decrease | -2.46 | 0.02337605 | 0.3495 | 5-azacytidine induced gene 1 (predicted) | PREDICTED: Rattus norvegicus 5-azacytidine induced gene 1 | XM_340945 |

| | | | | | | | | |
|---|---|---|---|---|---|---|---|---|
| | | | | | | | (predicted) (Azi1_predicted), mRNA [XM_340945] | |
| **-1.10** | 2.14 | Decrease | -2.46 | 0.02358261 | 0.3511 | FAD-dependent oxidoreductase domain containing 1 (predicted) | Rattus norvegicus hypothetical LOC315547 (LOC315547), mRNA [XM_235988] | XM_235988 |
| **1.79** | 3.45 | Increase | 2.45 | 0.02374693 | 0.3522 | BF564703 | UI-R-BU0-amv-g-06-0-UI.r1 UI-R-BU0 Rattus norvegicus cDNA clone UI-R-BU0-amv-g-06-0-UI 5', mRNA sequence [BF564703] | BF564703 |
| **-1.69** | 3.23 | Decrease | -2.45 | 0.02409551 | 0.3538 | XM_341060 | Rattus norvegicus similar to Williams-Beuren syndrome critical region protein 27 (LOC360794), mRNA [XM_341060] | XM_341060 |
| **1.32** | 2.49 | Increase | 2.45 | 0.02410141 | 0.3538 | junction adhesion molecule 2 | Rattus norvegicus junction adhesion molecule 2 (Jam2), mRNA [NM_001034004] | NM_001034004 |
| **-1.01** | 2.01 | Decrease | -2.44 | 0.02416909 | 0.3539 | AW917678 | AW917678 EST348982 Rat gene index, normalized rat, norvegicus, Bento Soares Rattus norvegicus cDNA clone RGIEG73 5' end, mRNA sequence [AW917678] | AW917678 |
| **-1.48** | 2.78 | Decrease | -2.44 | 0.02419229 | 0.3539 | POU domain, class 2, transcription factor 1 | PREDICTED: Rattus norvegicus POU domain, class 2, transcription factor 1 (Pou2f1), mRNA [XM_001075635] | XM_001075635 |
| **1.26** | 2.39 | Increase | 2.44 | 0.02420531 | 0.3539 | SRY-box containing gene 21 (predicted) | PREDICTED: Rattus norvegicus SRY-box containing gene 21 (predicted) (Sox21_predicted), mRNA [XM_224521] | XM_224521 |
| **-2.06** | 4.16 | Decrease | -2.44 | 0.02447062 | 0.3560 | AW143180 | AW143180 EST293476 Normalized rat brain, Bento Soares Rattus sp. cDNA clone RGIBF05 5' end, mRNA sequence [AW143180] | AW143180 |

| | | | | | | | | |
|---|---|---|---|---|---|---|---|---|
| **-1.73** | 3.32 | Decrease | -2.44 | 0.02449439 | 0.3561 | growth differentiation factor 11 | PREDICTED: Rattus norvegicus growth differentiation factor 11 (Gdf11), mRNA [XM_001071574] | XM_001071574 |
| **1.13** | 2.19 | Increase | 2.43 | 0.0248293 | 0.3587 | coronin, actin binding protein 6 | Rattus norvegicus coronin, actin binding protein 6 (Coro6), mRNA [NM_139115] | NM_139115 |
| **-1.15** | 2.21 | Decrease | -2.43 | 0.02486483 | 0.3587 | CUG triplet repeat, RNA binding protein 2 | Rattus norvegicus CUG triplet repeat, RNA binding protein 2 (Cugbp2), mRNA [NM_017197] | NM_017197 |
| **-2.43** | 5.40 | Decrease | -2.43 | 0.02486515 | 0.3587 | epsin 3 | Rattus norvegicus epsin 3 (Epn3), mRNA [NM_001024791] | NM_001024791 |
| **1.40** | 2.63 | Increase | 2.43 | 0.02498064 | 0.3597 | procollagen, type V, alpha 1 | Rattus norvegicus procollagen, type V, alpha 1 (Col5a1), mRNA [NM_134452] | NM_134452 |
| **1.43** | 2.69 | Increase | 2.42 | 0.02522654 | 0.3615 | transcriptional co-activator with PDZ-binding motif (TA)Z | Rattus norvegicus transcriptional co-activator with PDZ-binding motif (TA)Z (MGC116096), mRNA [NM_001024869] | NM_001024869 |
| **-1.94** | 3.84 | Decrease | -2.42 | 0.02534621 | 0.3621 | laminin, beta 3 | PREDICTED: Rattus norvegicus laminin, beta 3 (Lamb3), mRNA [XM_001069930] | XM_001069930 |
| **1.10** | 2.14 | Increase | 2.42 | 0.02550939 | 0.3626 | leucine rich repeat containing 33 | Rattus norvegicus leucine rich repeat containing 33 (Lrrc33), mRNA [NM_001024995] | NM_001024995 |
| **-1.21** | 2.31 | Decrease | -2.42 | 0.02550979 | 0.3626 | tetraspanin 1 | Rattus norvegicus tetraspanin 1 (Tspan1), mRNA [NM_001004236] | NM_001004236 |
| **-1.33** | 2.51 | Decrease | -2.42 | 0.02561668 | 0.3632 | similar to ornithine decarboxylase-like protein | Rattus norvegicus similar to ornithine decarboxylase-like protein (LOC366473), mRNA [NM_001014261] | NM_001014261 |
| **1.02** | 2.03 | Increase | 2.42 | 0.025695 | 0.3636 | ELK3, member of ETS oncogene family (predicted) | PREDICTED: Rattus norvegicus ELK3, member of ETS oncogene family (predicted) (Elk3_predicted), mRNA [XM_343198] | XM_343198 |

| | | | | | | | | |
|---|---|---|---|---|---|---|---|---|
| **1.22** | 2.33 | Increase | 2.42 | 0.02569629 | 0.3636 | AW915440 | AW915440 EST346744 Normalized rat embryo, Bento Soares Rattus sp. cDNA clone RGICV43 5' end, mRNA sequence [AW915440] | AW915440 |
| **-1.39** | 2.61 | Decrease | -2.41 | 0.02581236 | 0.3639 | laminin, beta 3 | PREDICTED: Rattus norvegicus laminin, beta 3 (Lamb3), mRNA [XM_223087] | XM_223087 |
| **1.05** | 2.07 | Increase | 2.41 | 0.02584516 | 0.3639 | TC538123 | Q7Z5L9 (Q7Z5L9) Interferon regulatory factor-2 binding protein 2A, partial (64%) [TC538123] | |
| **-1.30** | 2.47 | Decrease | -2.41 | 0.02602022 | 0.3639 | zinc fingers and homeoboxes 3 | PREDICTED: Rattus norvegicus zinc fingers and homeoboxes 3, transcript variant 2 (Zhx3), mRNA [XM_230811] | XM_230811 |
| **1.30** | 2.47 | Increase | 2.41 | 0.02609978 | 0.3639 | AI231472 | AI231472 EST228160 Normalized rat embryo, Bento Soares Rattus sp. cDNA clone REMDK57 3' end, mRNA sequence [AI231472] | AI231472 |
| **-1.23** | 2.34 | Decrease | -2.41 | 0.02620741 | 0.3646 | similar to ZFP36L3 (predicted) | PREDICTED: Rattus norvegicus similar to ZFP36L3 (predicted) (RGD1559581_predicted), mRNA [XM_228661] | XM_228661 |
| **-1.94** | 3.83 | Decrease | -2.40 | 0.02640348 | 0.3649 | Syncrip | PREDICTED: Rattus norvegicus synaptotagmin binding, cytoplasmic RNA interacting protein (Syncrip), mRNA [XM_001065902] | XM_001065902 |
| **1.05** | 2.07 | Increase | 2.40 | 0.02643124 | 0.3649 | XM_346356 | Rattus norvegicus similar to 60S RIBOSOMAL PROTEIN L7 (LOC367933), mRNA [XM_346356] | XM_346356 |
| **-1.11** | 2.16 | Decrease | -2.40 | 0.02645783 | 0.3650 | similar to Myosin-18B (Myosin XVIIIb) | Rattus norvegicus similar to myosin-like protein (LOC304551), mRNA [XM_239684] | XM_239684 |
| **1.98** | 3.94 | Increase | 2.40 | 0.02652971 | 0.3655 | arachidonate 5-lipoxygenase activating protein | Rattus norvegicus arachidonate 5-lipoxygenase activating protein (Alox5ap), mRNA [NM_017260] | NM_017260 |

| | | | | | | | | |
|---|---|---|---|---|---|---|---|---|
| **1.70** | 3.24 | Increase | 2.40 | 0.02658955 | 0.3658 | myelin-associated glycoprotein | Rattus norvegicus myelin-associated glycoprotein (Mag), mRNA [NM_017190] | NM_017190 |
| **1.49** | 2.81 | Increase | 2.40 | 0.02668094 | 0.3663 | similar to Slit-like 2 | PREDICTED: Rattus norvegicus similar to Slit-like 2 (LOC679921), mRNA [XM_001055204] | XM_001055204 |
| **1.30** | 2.46 | Increase | 2.39 | 0.02681878 | 0.3668 | proline-rich proteoglycan 2 | Rattus norvegicus proline-rich proteoglycan 2 (Prpg2), mRNA [NM_172065] | NM_172065 |
| **-2.14** | 4.40 | Decrease | -2.39 | 0.0269002 | 0.3674 | CD6 antigen | Rattus norvegicus CD6 antigen (Cd6), mRNA [NM_175577] | NM_175577 |
| **-1.68** | 3.20 | Decrease | -2.39 | 0.02700562 | 0.3676 | pellino homolog 3 (Drosophila) (predicted) | PREDICTED: Rattus norvegicus pellino homolog 3 (Drosophila) (predicted) (Peli3_predicted), mRNA [XM_219692] | XM_219692 |
| **1.38** | 2.60 | Increase | 2.38 | 0.02744446 | 0.3694 | AW144408 | AW144408 EST294704 Normalized rat ovary, Bento Soares Rattus sp. cDNA clone RGICR81 5' end, mRNA sequence [AW144408] | AW144408 |
| **2.12** | 4.35 | Increase | 2.38 | 0.02745857 | 0.3694 | retinol binding protein 1, cellular | Rattus norvegicus retinol binding protein 1, cellular (Rbp1), mRNA [NM_012733] | NM_012733 |
| **-1.10** | 2.15 | Decrease | -2.38 | 0.02748495 | 0.3694 | TC553545 | Q961E9 (Q961E9) GH28016p, partial (4%) [TC553545] | |
| **-1.15** | 2.22 | Decrease | -2.38 | 0.02758548 | 0.3696 | Vcl_predicted | PREDICTED: Rattus norvegicus vinculin (predicted) (Vcl_predicted), mRNA [XM_223781] | XM_223781 |
| **1.31** | 2.48 | Increase | 2.38 | 0.0279174 | 0.3714 | similar to RIKEN cDNA 1810012H11 | Rattus norvegicus similar to RIKEN cDNA 1810012H11 (LOC298606), mRNA [NM_001013936] | NM_001013936 |
| **-1.34** | 2.53 | Decrease | -2.37 | 0.02798333 | 0.3719 | CA507012 | CA507012 UI-R-FS1-cqd-e-23-0-UI.s1 NCI_CGAP_FS1 Rattus norvegicus cDNA clone IMAGE:7361377 3', mRNA sequence [CA507012] | CA507012 |

| | | | | | | | | |
|---|---|---|---|---|---|---|---|---|
| **-1.14** | 2.20 | Decrease | -2.37 | 0.02808914 | 0.3719 | EGF-like-domain, multiple 4 | PREDICTED: Rattus norvegicus EGF-like-domain, multiple 4 (Egfl4), mRNA [XM_341803] | XM_341803 |
| **-1.75** | 3.35 | Decrease | -2.37 | 0.02846067 | 0.3735 | AW918794 | AW918794 EST350098 Rat gene index, normalized rat, norvegicus, Bento Soares Rattus norvegicus cDNA clone RGIEY31 5' end, mRNA sequence [AW918794] | AW918794 |
| **-1.18** | 2.27 | Decrease | -2.36 | 0.0285873 | 0.3743 | AI555010 | AI555010 UI-R-C2p-qu-h-04-0-UI.s1 UI-R-C2p Rattus norvegicus cDNA clone UI-R-C2p-qu-h-04-0-UI 3', mRNA sequence [AI555010] | AI555010 |
| **-1.12** | 2.17 | Decrease | -2.36 | 0.02866495 | 0.3745 | Rab6 interacting protein 1 (predicted) | PREDICTED: Rattus norvegicus Rab6 interacting protein 1 (predicted) (Rab6ip1_predicted), mRNA [XM_219270] | XM_219270 |
| **1.63** | 3.10 | Increase | 2.36 | 0.0288298 | 0.3745 | WD repeat and SOCS box-containing 1 | Rattus norvegicus WD repeat and SOCS box-containing 1 (Wsb1), transcript variant 2, mRNA [NM_001025664] | NM_001025664 |
| **-1.03** | 2.04 | Decrease | -2.36 | 0.02885336 | 0.3745 | U08214 | RSU08214 Rattus sp. DNA binding protein (URE-B1) mRNA, complete cds [U08214] | U08214 |
| **1.46** | 2.75 | Increase | 2.36 | 0.02897611 | 0.3748 | DV722215 | RVL13603 Wackym-Soares normalized rat vestibular cDNA library Rattus norvegicus cDNA 5', mRNA sequence [DV722215] | DV722215 |
| **1.00** | 2.00 | Increase | 2.35 | 0.02929268 | 0.3769 | similar to CD69 antigen (p60, early T-cell activation antigen) (predicted) | PREDICTED: Rattus norvegicus similar to CD69 antigen (p60, early T-cell activation antigen) (predicted) (RGD1565373_predicted), mRNA [XM_231622] | XM_231622 |

| | | | | | | | | |
|---|---|---|---|---|---|---|---|---|
| **-1.58** | 2.99 | Decrease | -2.35 | 0.02940474 | 0.3769 | valyl-tRNA synthetase 2 | PREDICTED: Rattus norvegicus valyl-tRNA synthetase 2 (Vars2), mRNA [XM_001076616] | XM_001076616 |
| **-1.36** | 2.56 | Decrease | -2.35 | 0.02945723 | 0.3769 | interleukin 13 | Rattus norvegicus interleukin 13 (Il13), mRNA [NM_053828] | NM_053828 |
| **-2.06** | 4.16 | Decrease | -2.35 | 0.02949021 | 0.3769 | titin | RNU89530 Rattus norvegicus titin protein homolog mRNA, partial cds [U89530] | U89530 |
| **-1.12** | 2.18 | Decrease | -2.35 | 0.02971844 | 0.3781 | AW144299 | AW144299 EST294595 Normalized rat ovary, Bento Soares Rattus sp. cDNA clone RGICP57 5' end, mRNA sequence [AW144299] | AW144299 |
| **-1.39** | 2.62 | Decrease | -2.34 | 0.02987516 | 0.3785 | myosin, heavy polypeptide 14 | PREDICTED: Rattus norvegicus myosin, heavy polypeptide 14 (Myh14), mRNA [XM_218617] | XM_218617 |
| **2.34** | 5.07 | Increase | 2.34 | 0.02988069 | 0.3785 | XM_345191 | Rattus norvegicus similar to ribosomal protein L30 (LOC365749), mRNA [XM_345191] | XM_345191 |
| **1.15** | 2.23 | Increase | 2.34 | 0.02993471 | 0.3785 | F11 receptor | Rattus norvegicus F11 receptor (F11r), mRNA [NM_053796] | NM_053796 |
| **-1.13** | 2.18 | Decrease | -2.34 | 0.02999332 | 0.3790 | XM_346113 | PREDICTED: Rattus norvegicus similar to Down syndrome cell adhesion molecule (LOC367375), mRNA [XM_346113] | XM_346113 |
| **-1.58** | 2.98 | Decrease | -2.34 | 0.03023716 | 0.3802 | AW918794 | AW918794 EST350098 Rat gene index, normalized rat, norvegicus, Bento Soares Rattus norvegicus cDNA clone RGIEY31 5' end, mRNA sequence [AW918794] | AW918794 |
| **-1.09** | 2.12 | Decrease | -2.34 | 0.03024991 | 0.3802 | Sbno1 | PREDICTED: Rattus norvegicus sno, strawberry notch homolog 1 (Drosophila) (Sbno1), mRNA [XM_222152] | XM_222152 |

| | | | | | | | | |
|---|---|---|---|---|---|---|---|---|
| **1.21** | 2.32 | Increase | 2.33 | 0.0305071 | 0.3815 | neuroblastoma, suppression of tumorigenicity 1 | Rattus norvegicus neuroblastoma, suppression of tumorigenicity 1 (Nbl1), mRNA [NM_031609] | NM_031609 |
| **1.26** | 2.39 | Increase | 2.33 | 0.03051087 | 0.3815 | transmembrane protein 104 (predicted) | PREDICTED: Rattus norvegicus transmembrane protein 104 (predicted) (Tmem104_predicted), mRNA [XM_221104] | XM_221104 |
| **-1.69** | 3.23 | Decrease | -2.33 | 0.03053483 | 0.3817 | homer homolog 2 (Drosophila) | Rattus norvegicus homer homolog 2 (Drosophila) (Homer2), mRNA [NM_053309] | NM_053309 |
| **1.18** | 2.26 | Increase | 2.33 | 0.03078096 | 0.3835 | XM_217340 | Rattus norvegicus similar to hypothetical protein MGC19067 (LOC301239), mRNA [XM_217340] | XM_217340 |
| **1.00** | 2.00 | Increase | 2.33 | 0.03092873 | 0.3844 | gap junction membrane channel protein alpha 7 | PREDICTED: Rattus norvegicus gap junction membrane channel protein alpha 7 (Gja7), mRNA [XM_001081521] | XM_001081521 |
| **1.70** | 3.24 | Increase | 2.33 | 0.03094604 | 0.3844 | paired related homeobox 2 (predicted) | PREDICTED: Rattus norvegicus paired related homeobox 2 (predicted) (Prrx2_predicted), mRNA [XM_238327] | XM_238327 |
| **2.49** | 5.64 | Increase | 2.33 | 0.03099063 | 0.3846 | AW533905 | AW533905 UI-R-C4-alr-b-02-0-UI.s1 UI-R-C4 Rattus norvegicus cDNA clone UI-R-C4-alr-b-02-0-UI 3', mRNA sequence [AW533905] | AW533905 |
| **1.12** | 2.18 | Increase | 2.32 | 0.03123406 | 0.3854 | parathymosin | Rattus norvegicus parathymosin (Ptms), mRNA [NM_031975] | NM_031975 |
| **1.11** | 2.16 | Increase | 2.32 | 0.03159451 | 0.3869 | BF555800 | BF555800 UI-R-A1-ej-d-01-0-UI.r1 UI-R-A1 Rattus norvegicus cDNA clone UI-R-A1-ej-d-01-0-UI 5', mRNA sequence [BF555800] | BF555800 |
| **1.03** | 2.04 | Increase | 2.31 | 0.03169803 | 0.3876 | RT1 class II, locus Da | Rattus norvegicus RT1 class II, locus Da (RT1-Da), mRNA [NM_001008847] | NM_001008847 |

| | | | | | | | | |
|---|---|---|---|---|---|---|---|---|
| **1.49** | 2.82 | Increase | 2.30 | 0.03236966 | 0.3912 | mast cell protease 6 | Rattus norvegicus mast cell protease 6 (Mcpt6), mRNA [NM_019180] | NM_019180 |
| **1.25** | 2.39 | Increase | 2.30 | 0.03262557 | 0.3923 | tissue factor pathway inhibitor | Rattus norvegicus tissue factor pathway inhibitor (Tfpi), mRNA [NM_017200] | NM_017200 |
| **1.06** | 2.08 | Increase | 2.30 | 0.03263429 | 0.3923 | lipocalin 7 | Rattus norvegicus lipocalin 7 (Lcn7), mRNA [NM_053582] | NM_053582 |
| **1.36** | 2.56 | Increase | 2.30 | 0.03284202 | 0.3931 | BI285576 | BI285576 UI-R-CW0s-ccb-g-06-0-UI.s1 UI-R-CW0s Rattus norvegicus cDNA clone UI-R-CW0s-ccb-g-06-0-UI 3', mRNA sequence [BI285576] | BI285576 |
| **1.13** | 2.18 | Increase | 2.30 | 0.03293153 | 0.3934 | AW917911 | AW917911 EST349215 Rat gene index, normalized rat, norvegicus, Bento Soares Rattus norvegicus cDNA clone RGIEK03 5' end, mRNA sequence [AW917911] | AW917911 |
| **-1.12** | 2.17 | Decrease | -2.29 | 0.03310292 | 0.3942 | CO398332 | AGENCOURT_27827173 NIH_MGC_252 Rattus norvegicus cDNA clone IMAGE:7308022 5', mRNA sequence [CO398332] | CO398332 |
| **-1.12** | 2.18 | Decrease | -2.29 | 0.03312584 | 0.3942 | similar to Retinoic acid induced 2 (predicted) | PREDICTED: Rattus norvegicus similar to Retinoic acid induced 2 (predicted) (RGD1560139_predicted), mRNA [XM_576959] | XM_576959 |
| **-1.04** | 2.05 | Decrease | -2.29 | 0.03340342 | 0.3946 | protein phosphatase 1B, magnesium dependent, beta isoform | Rattus norvegicus protein phosphatase 1B, magnesium dependent, beta isoform (Ppm1b), mRNA [NM_033096] | NM_033096 |
| **-1.13** | 2.19 | Decrease | -2.29 | 0.03347089 | 0.3949 | nuclear factor I/B | PREDICTED: Rattus norvegicus nuclear factor I/B (Nfib), mRNA [XM_342854] | XM_342854 |

| | | | | | | | | |
|---|---|---|---|---|---|---|---|---|
| **1.30** | 2.46 | Increase | 2.29 | 0.03352783 | 0.3952 | glutamate receptor, metabotropic 2 | PREDICTED: Rattus norvegicus glutamate receptor, metabotropic 2 (Grm2), mRNA [XM_343470] | XM_343470 |
| **1.00** | 2.00 | Increase | 2.28 | 0.03369648 | 0.3959 | AI045508 | AI045508 UI-R-C1-kj-g-08-0-UI.s1 UI-R-C1 Rattus norvegicus cDNA clone UI-R-C1-kj-g-08-0-UI 3', mRNA sequence [AI045508] | AI045508 |
| **1.01** | 2.01 | Increase | 2.28 | 0.0337376 | 0.3959 | BF417776 | BF417776 UI-R-CN0-blx-a-09-0-UI.s1 UI-R-CN0 Rattus norvegicus cDNA clone UI-R-CN0-blx-a-09-0-UI 3', mRNA sequence [BF417776] | BF417776 |
| **-1.11** | 2.16 | Decrease | -2.28 | 0.0337597 | 0.3959 | ATPase, Ca++ transporting, plasma membrane 3 | PREDICTED: Rattus norvegicus ATPase, Ca++ transporting, plasma membrane 3 (Atp2b3), mRNA [XM_343839] | XM_343839 |
| **1.83** | 3.57 | Increase | 2.28 | 0.03376864 | 0.3959 | Jun D proto-oncogene | Rattus norvegicus Jun D proto-oncogene (Jund), mRNA [NM_138875] | NM_138875 |
| **-1.31** | 2.47 | Decrease | -2.28 | 0.03387811 | 0.3964 | integrin alpha 7 | Rattus norvegicus integrin alpha 7 (Itga7), mRNA [NM_030842] | NM_030842 |
| **1.09** | 2.14 | Increase | 2.28 | 0.03392 | 0.3967 | similar to nuclear localized factor 2 | PREDICTED: Rattus norvegicus similar to nuclear localized factor 2 (LOC501015), mRNA [XM_001056441] | XM_001056441 |
| **1.37** | 2.59 | Increase | 2.28 | 0.03393656 | 0.3967 | lysyl oxidase-like 1 | Rattus norvegicus lysyl oxidase-like 1 (Loxl1), mRNA [NM_001012125] | NM_001012125 |
| **1.51** | 2.84 | Increase | 2.28 | 0.03395108 | 0.3967 | HtrA serine peptidase 3 (predicted) | PREDICTED: Rattus norvegicus HtrA serine peptidase 3 (predicted) (Htra3_predicted), mRNA [XM_341237] | XM_341237 |
| **-1.58** | 2.99 | Decrease | -2.28 | 0.03415916 | 0.3982 | Ttn | PREDICTED: Rattus norvegicus titin (Ttn), mRNA [XM_001065955] | XM_001065955 |
| **-1.02** | 2.02 | Decrease | -2.28 | 0.03418866 | 0.3983 | AI411407 | AI411407 EST239701 Normalized rat kidney, Bento Soares Rattus sp. | AI411407 |

| | | | | | | | | |
|---|---|---|---|---|---|---|---|---|
| | | | | | | | cDNA clone RKIES92 3' end, mRNA sequence [AI411407] | |
| **1.39** | 2.63 | Increase | 2.28 | 0.03431443 | 0.3988 | TC518066 | Q9ERK2 (Q9ERK2) Neprilysin-like peptidase gamma, partial (3%) [TC518066] | |
| **1.09** | 2.13 | Increase | 2.28 | 0.03434051 | 0.3990 | quinolinate phosphoribosyltransferase | Rattus norvegicus quinolinate phosphoribosyltransferase (Qprt), mRNA [NM_001009646] | NM_001009646 |
| **1.03** | 2.04 | Increase | 2.27 | 0.03443671 | 0.3993 | hsp70-interacting protein | Rattus norvegicus hsp70-interacting protein (Hspbp1), mRNA [NM_139261] | NM_139261 |
| **1.21** | 2.31 | Increase | 2.27 | 0.03451459 | 0.3993 | farnesyltransferase, CAAX box, beta | Rattus norvegicus farnesyltransferase, CAAX box, beta (Fntb), mRNA [NM_172034] | NM_172034 |
| **1.50** | 2.83 | Increase | 2.27 | 0.03456926 | 0.3995 | A kinase (PRKA) anchor protein (gravin) 12 | Rattus norvegicus A kinase (PRKA) anchor protein (gravin) 12 (Akap12), transcript variant 1, mRNA [NM_057103] | NM_057103 |
| **-1.19** | 2.29 | Decrease | -2.27 | 0.03475208 | 0.3999 | similar to RIKEN cDNA B630019K06 (predicted) | Rattus norvegicus similar to hypothetical protein MGC31104 (LOC363449), mRNA [XM_343770] | XM_343770 |
| **1.33** | 2.51 | Increase | 2.27 | 0.03479267 | 0.3999 | XM_227418 | Rattus norvegicus similar to hypothetical protein FLJ32934 (LOC295247), mRNA [XM_227418] | XM_227418 |
| **1.54** | 2.91 | Increase | 2.27 | 0.03480154 | 0.3999 | CO386194 | AGENCOURT_26627340 NIH_MGC_253 Rattus norvegicus cDNA clone IMAGE:7302287 5', mRNA sequence [CO386194] | CO386194 |
| **-1.53** | 2.90 | Decrease | -2.26 | 0.03513186 | 0.4012 | similar to Glutathione S-transferase 8 (GST 8-8) (Chain 8) (GST class-alpha) (predicted) | PREDICTED: Rattus norvegicus similar to Glutathione S-transferase 8 (GST 8-8) (Chain 8) (GST class-alpha) (predicted) (RGD1564906_predicted), mRNA [XM_576039] | XM_576039 |

| | | | | | | | | |
|---|---|---|---|---|---|---|---|---|
| **1.04** | 2.05 | Increase | 2.26 | 0.0353762 | 0.4020 | similar to Laminin alpha-4 chain precursor (predicted) | PREDICTED: Rattus norvegicus similar to Laminin alpha-4 chain precursor (predicted) (RGD1560062_predicted), mRNA [XM_228209] | XM_228209 |
| **-1.30** | 2.46 | Decrease | -2.26 | 0.03553779 | 0.4026 | similar to hypothetical basic protein I-19 (predicted) | PREDICTED: Rattus norvegicus similar to hypothetical basic protein I-19 (predicted) (RGD1305157_predicted), mRNA [XM_236724] | XM_236724 |
| **-1.73** | 3.32 | Decrease | -2.26 | 0.03575581 | 0.4034 | glycine-, glutamate-, thienylcyclohexylpiperidine-binding protein | PREDICTED: Rattus norvegicus glycine-, glutamate-, thienylcyclohexylpiperidine-binding protein (LOC246295), mRNA [XM_575968] | XM_575968 |
| **-2.22** | 4.66 | Decrease | -2.25 | 0.03601868 | 0.4047 | BC099186 | Rattus norvegicus cDNA clone MGC:116357 IMAGE:7383333, complete cds. [BC099186] | BC099186 |
| **1.32** | 2.49 | Increase | 2.25 | 0.03619798 | 0.4051 | phosphatase, orphan 1 (predicted) | PREDICTED: Rattus norvegicus phosphatase, orphan 1 (predicted) (Phospho1_predicted), mRNA [XM_220877] | XM_220877 |
| **1.93** | 3.82 | Increase | 2.25 | 0.03638548 | 0.4059 | deoxyribonuclease II | Rattus norvegicus deoxyribonuclease II (Dnase2), mRNA [NM_138539] | NM_138539 |
| **2.60** | 6.08 | Increase | 2.25 | 0.03647912 | 0.4061 | complement component 1, q subcomponent, gamma polypeptide | Rattus norvegicus complement component 1, q subcomponent, gamma polypeptide (C1qg), mRNA [NM_001008524] | NM_001008524 |
| **1.09** | 2.13 | Increase | 2.25 | 0.0364859 | 0.4061 | AW142493 | AW142493 EST292745 Normalized rat embryo, Bento Soares Rattus sp. cDNA clone RGIAN21 5' end, mRNA sequence [AW142493] | AW142493 |

| | | | | | | | | |
|---|---|---|---|---|---|---|---|---|
| **1.21** | 2.32 | Increase | 2.25 | 0.03652906 | 0.4061 | adipocyte-specific adhesion molecule | Rattus norvegicus adipocyte-specific adhesion molecule (Asam), mRNA [NM_173154] | NM_173154 |
| **1.66** | 3.15 | Increase | 2.24 | 0.03669018 | 0.4064 | protein C receptor, endothelial | Rattus norvegicus protein C receptor, endothelial (Procr), mRNA [NM_001025733] | NM_001025733 |
| **-1.55** | 2.94 | Decrease | -2.24 | 0.03678111 | 0.4064 | XM_234810 | Rattus norvegicus similar to GLUTATHIONE S-TRANSFERASE 8 (GST 8-8) (CHAIN 8) (GST CLASS-ALPHA) (LOC314570), mRNA [XM_234810] | XM_234810 |
| **-1.21** | 2.32 | Decrease | -2.24 | 0.03683957 | 0.4065 | N-myc downstream regulated gene 4 | Rattus norvegicus N-myc downstream regulated gene 4 (Ndrg4), mRNA [NM_031967] | NM_031967 |
| **-1.42** | 2.67 | Decrease | -2.24 | 0.03690091 | 0.4066 | similar to KIAA0368 (predicted) | Rattus norvegicus similar to KIAA0368 (LOC313196), mRNA [XM_232937] | XM_232937 |
| **1.70** | 3.24 | Increase | 2.24 | 0.03719753 | 0.4088 | similar to RIKEN cDNA 1300017J02 | PREDICTED: Rattus norvegicus similar to RIKEN cDNA 1300017J02 (RGD1310507), mRNA [XM_236574] | XM_236574 |
| **1.04** | 2.06 | Increase | 2.24 | 0.03721769 | 0.4088 | reticulon 4 receptor-like 2 | Rattus norvegicus reticulon 4 receptor-like 2 (Rtn4rl2), mRNA [NM_181380] | NM_181380 |
| **-1.17** | 2.25 | Decrease | -2.23 | 0.03764038 | 0.4102 | Mapk7 | Rattus norvegicus mitogen-activated protein kinase 7 (Mapk7), mRNA [XM_340813] | XM_340813 |
| **2.17** | 4.50 | Increase | 2.23 | 0.03804436 | 0.4123 | LRRGT00154 | PREDICTED: Rattus norvegicus LRRGT00154 (LOC499544), mRNA [XM_574867] | XM_574867 |
| **1.65** | 3.15 | Increase | 2.22 | 0.03812488 | 0.4126 | chymase 1, mast cell | Rattus norvegicus chymase 1, mast cell (Cma1), mRNA [NM_013092] | NM_013092 |
| **-1.38** | 2.61 | Decrease | -2.22 | 0.03837913 | 0.4131 | solute carrier family 27 (fatty acid transporter), member 1 | Rattus norvegicus solute carrier family 27 (fatty acid transporter), | NM_053580 |

| | | | | | | | | |
|---|---|---|---|---|---|---|---|---|
| | | | | | | | member 1 (Slc27a1), mRNA [NM_053580] | |
| **-2.42** | 5.36 | Decrease | -2.22 | 0.03847613 | 0.4133 | cholinergic receptor, nicotinic, alpha polypeptide 7 | Rattus norvegicus cholinergic receptor, nicotinic, alpha polypeptide 7 (Chrna7), mRNA [NM_012832] | NM_012832 |
| **-1.33** | 2.51 | Decrease | -2.22 | 0.0385194 | 0.4134 | kinesin family member 21A (predicted) | PREDICTED: Rattus norvegicus kinesin family member 21A (predicted) (Kif21a_predicted), mRNA [XM_001056804] | XM_001056804 |
| **1.26** | 2.40 | Increase | 2.21 | 0.03920549 | 0.4170 | serine/threonine kinase 25 (STE20 homolog, yeast) | Rattus norvegicus serine/threonine kinase 25 (STE20 homolog, yeast) (Stk25), mRNA [NM_184049] | NM_184049 |
| **-1.10** | 2.15 | Decrease | -2.21 | 0.03945026 | 0.4175 | carbonic anhydrase 1 (predicted) | PREDICTED: Rattus norvegicus carbonic anhydrase 1 (predicted) (Car1_predicted), mRNA [XM_226922] | XM_226922 |
| **-1.18** | 2.26 | Decrease | -2.21 | 0.03949196 | 0.4175 | similar to Guanine nucleotide-binding protein G(T) gamma-T1 subunit precursor (Transducin gamma chain) | AW533257 UI-R-BU0-ana-f-11-0-UI.s1 UI-R-BU0 Rattus norvegicus cDNA clone UI-R-BU0-ana-f-11-0-UI 3', mRNA sequence [AW533257] | AW533257 |
| **-2.38** | 5.19 | Decrease | -2.20 | 0.03974168 | 0.4189 | BF281791 | BF281791 EST446382 Rat Gene Index, normalized rat, Rattus norvegicus cDNA Rattus norvegicus cDNA clone RGIBA05 similar to alpha cardiac myosin heavy chain, mRNA sequence [BF281791] | BF281791 |
| **-1.65** | 3.15 | Decrease | -2.20 | 0.03981819 | 0.4193 | cell division cycle 25 homolog A (S. cerevisiae) | Rattus norvegicus cell division cycle 25 homolog A (S. cerevisiae) (Cdc25a), mRNA [NM_133571] | NM_133571 |
| **1.15** | 2.22 | Increase | 2.20 | 0.04002754 | 0.4203 | zinc finger, FYVE domain containing 1 (predicted) | PREDICTED: Rattus norvegicus zinc finger, FYVE domain containing 1 (predicted) (Zfyve1_predicted), mRNA [XM_216762] | XM_216762 |

| | | | | | | | | |
|---|---|---|---|---|---|---|---|---|
| **1.29** | 2.44 | Increase | 2.19 | 0.0405062 | 0.4222 | similar to hypothetical protein FLJ21827 | Rattus norvegicus similar to hypothetical protein FLJ21827 (RGD1307682), mRNA [NM_001024760] | NM_001024760 |
| **-1.01** | 2.01 | Decrease | -2.19 | 0.04092458 | 0.4247 | BG664520 | BG664520 DRABEH09 Rat DRG Library Rattus norvegicus cDNA clone DRABEH09 5', mRNA sequence [BG664520] | BG664520 |
| **1.74** | 3.33 | Increase | 2.19 | 0.04093541 | 0.4247 | XM_227913 | Rattus norvegicus similar to RIKEN cDNA 4930580F03 (LOC294168), mRNA [XM_227913] | XM_227913 |
| **-1.92** | 3.79 | Decrease | -2.19 | 0.04105345 | 0.4248 | secreted phosphoprotein 1 | Rattus norvegicus secreted phosphoprotein 1 (Spp1), mRNA [NM_012881] | NM_012881 |
| **1.09** | 2.12 | Increase | 2.19 | 0.04118983 | 0.4254 | similar to ribosomal protein L10 | PREDICTED: Rattus norvegicus similar to ribosomal protein L10 (LOC689075), mRNA [XM_001069421] | XM_001069421 |
| **1.26** | 2.39 | Increase | 2.18 | 0.04142679 | 0.4261 | procollagen, type XI, alpha 2 (mapped) | Rattus norvegicus procollagen, type XI, alpha 2 (mapped) (Col11a2_mapped), mRNA [NM_212528] | NM_212528 |
| **1.15** | 2.22 | Increase | 2.18 | 0.0415061 | 0.4261 | lysyl oxidase | Rattus norvegicus lysyl oxidase (Lox), mRNA [NM_017061] | NM_017061 |
| **1.01** | 2.02 | Increase | 2.18 | 0.04175704 | 0.4269 | XM_346101 | Rattus norvegicus similar to ribosomal protein L30 (LOC367349), mRNA [XM_346101] | XM_346101 |
| **-1.06** | 2.08 | Decrease | -2.18 | 0.04198626 | 0.4271 | RGD1564173_predicted | PREDICTED: Rattus norvegicus similar to RIKEN cDNA 1700001F22 (predicted) (RGD1564173_predicted), mRNA [XM_579040] | XM_579040 |
| **-1.27** | 2.42 | Decrease | -2.17 | 0.04219395 | 0.4282 | leucine rich repeat protein 2, neuronal (predicted) | PREDICTED: Rattus norvegicus leucine rich repeat protein 2, | XM_222670 |

| | | | | | | | | |
|---|---|---|---|---|---|---|---|---|
| | | | | | | | neuronal (predicted) (Lrrn2_predicted), mRNA [XM_222670] | |
| **1.16** | 2.24 | Increase | 2.17 | 0.04228922 | 0.4286 | similar to RIKEN cDNA 2810002D13 gene | Rattus norvegicus similar to RIKEN cDNA 2810002D13 gene (RGD1307128), mRNA [NM_001012354] | NM_001012354 |
| **-1.48** | 2.79 | Decrease | -2.17 | 0.04246787 | 0.4293 | AW142013 | AW142013 EST292128 Normalized rat ovary, Bento Soares Rattus sp. cDNA clone RGICT53 5' end similar to basic-leucine zipper nuclear factor, mRNA sequence [AW142013] | AW142013 |
| **-1.10** | 2.15 | Decrease | -2.17 | 0.04278324 | 0.4310 | AI412098 | AI412098 EST240392 Normalized rat kidney, Bento Soares Rattus sp. cDNA clone RKIEJ68 3' end, mRNA sequence [AI412098] | AI412098 |
| **2.69** | 6.45 | Increase | 2.17 | 0.04289125 | 0.4315 | mast cell protease 1 | Rattus norvegicus mast cell protease 1 precursor (RMCP-1) mRNA, complete cds. [U67915] | U67915 |
| **2.48** | 5.57 | Increase | 2.17 | 0.04289192 | 0.4315 | secreted acidic cysteine rich glycoprotein | Rattus norvegicus secreted acidic cysteine rich glycoprotein (Sparc), mRNA [NM_012656] | NM_012656 |
| **-1.33** | 2.51 | Decrease | -2.16 | 0.04316576 | 0.4317 | similar to SH3-domain binding protein 3 | PREDICTED: Rattus norvegicus similar to SH3-domain binding protein 3 (LOC688018), mRNA [XR_009418] | XR_009418 |
| **1.29** | 2.45 | Increase | 2.16 | 0.04339813 | 0.4329 | TC523917 | 1913423B exoK gene. {Sinorhizobium meliloti;} , partial (6%) [TC523917] | |
| **-1.24** | 2.36 | Decrease | -2.16 | 0.04384894 | 0.4353 | M33313 | RATCYP2A21 Rat hepatic steroid hydroxylase IIA2 (CYP2A2) gene, exons 1 and 2 [M33313] | M33313 |
| **1.03** | 2.04 | Increase | 2.15 | 0.04393574 | 0.4354 | UDP-glucuronate decarboxylase 1 | Rattus norvegicus UDP-glucuronate decarboxylase 1 (Uxs1), mRNA [NM_139336] | NM_139336 |

| | | | | | | | | |
|---|---|---|---|---|---|---|---|---|
| **1.13** | 2.18 | Increase | 2.15 | 0.04405789 | 0.4354 | two pore channel 1 | Rattus norvegicus two pore channel 1 (Tpcn1), mRNA [NM_139332] | NM_139332 |
| **-2.06** | 4.17 | Decrease | -2.15 | 0.04412762 | 0.4354 | alpha thalassemia/mental retardation syndrome X-linked homolog (human) | PREDICTED: Rattus norvegicus alpha thalassemia/mental retardation syndrome X-linked homolog (human) (Atrx), mRNA [XM_217570] | XM_217570 |
| **1.96** | 3.89 | Increase | 2.15 | 0.04437498 | 0.4360 | carboxypeptidase A3 | PREDICTED: Rattus norvegicus carboxypeptidase A3 (Cpa3), mRNA [XM_342219] | XM_342219 |
| **1.24** | 2.35 | Increase | 2.15 | 0.04441238 | 0.4361 | LOC691522 | PREDICTED: Rattus norvegicus hypothetical protein LOC691522 (LOC691522), mRNA [XM_001078640] | XM_001078640 |
| **1.37** | 2.59 | Increase | 2.15 | 0.04456073 | 0.4364 | ARP3 actin-related protein 3 homolog (yeast) | Rattus norvegicus ARP3 actin-related protein 3 homolog (yeast) (Actr3), mRNA [NM_031068] | NM_031068 |
| **-1.16** | 2.23 | Decrease | -2.15 | 0.04463685 | 0.4364 | AABR03006190 | Rattus norvegicus chromosome 1, 7 clones, strain BN/SsNHsdMCW RNOR03210214, whole genome shotgun sequence [AABR03006190] | AABR03006190 |
| **1.60** | 3.04 | Increase | 2.15 | 0.04474353 | 0.4368 | AI233749 | AI233749 EST230437 Normalized rat kidney, Bento Soares Rattus sp. cDNA clone RKIDJ59 3' end, mRNA sequence [AI233749] | AI233749 |
| **-1.14** | 2.21 | Decrease | -2.15 | 0.04475269 | 0.4368 | CO396335 | AGENCOURT_26737670 NIH_MGC_255 Rattus norvegicus cDNA clone IMAGE:7317613 5', mRNA sequence [CO396335] | CO396335 |
| **1.14** | 2.20 | Increase | 2.14 | 0.04502713 | 0.4372 | cerebral endothelial cell adhesion molecule 1 | Rattus norvegicus cerebral endothelial cell adhesion molecule 1 (Ceecam1), mRNA [NM_001011962] | NM_001011962 |
| **1.20** | 2.30 | Increase | 2.14 | 0.04538006 | 0.4374 | AA925693 | AA925693 UI-R-A1-ec-a-04-0-UI.s1 UI-R-A1 Rattus norvegicus cDNA | AA925693 |

| | | | | | | | | |
|---|---|---|---|---|---|---|---|---|
| | | | | | | | clone UI-R-A1-ec-a-04-0-UI 3' similar to gi [AA925693] | |
| **-1.82** | 3.54 | Decrease | -2.14 | 0.04565938 | 0.4379 | pyrroline-5-carboxylate reductase-like | Rattus norvegicus pyrroline-5-carboxylate reductase-like (Pycrl), mRNA [NM_001011993] | NM_001011993 |
| **1.27** | 2.41 | Increase | 2.13 | 0.04598545 | 0.4390 | AW914967 | AW914967 EST346271 Normalized rat ovary, Bento Soares Rattus sp. cDNA clone RGIBM91 5' end, mRNA sequence [AW914967] | AW914967 |
| **1.05** | 2.07 | Increase | 2.12 | 0.04660006 | 0.4424 | cadherin EGF LAG seven-pass G-type receptor 1 | PREDICTED: Rattus norvegicus cadherin EGF LAG seven-pass G-type receptor 1 (Celsr1), mRNA [XM_001070474] | XM_001070474 |
| **-1.58** | 2.99 | Decrease | -2.12 | 0.04677122 | 0.4429 | leprecan-like 1 | Rattus norvegicus leprecan-like 1 (Leprel1), mRNA [NM_001025627] | NM_001025627 |
| **1.09** | 2.13 | Increase | 2.12 | 0.04684448 | 0.4431 | similar to RIKEN cDNA 9230117N10 | Rattus norvegicus similar to RIKEN cDNA 9230117N10 (RGD1311155), mRNA [NM_001014166] | NM_001014166 |
| **1.01** | 2.01 | Increase | 2.12 | 0.04690984 | 0.4432 | death-associated protein | Rattus norvegicus death-associated protein (Dap), mRNA [NM_022526] | NM_022526 |
| **1.06** | 2.09 | Increase | 2.12 | 0.04704079 | 0.4435 | LOC294446 | PREDICTED: Rattus norvegicus similar to Myristoylated alanine-rich C-kinase substrate (MARCKS) (Protein kinase C substrate 80 kDa protein), transcript variant 2 (LOC294446), mRNA [XM_001061084] | XM_001061084 |
| **-1.16** | 2.23 | Decrease | -2.12 | 0.04733898 | 0.4435 | transmembrane BAX inhibitor motif containing 1 | Rattus norvegicus transmembrane BAX inhibitor motif containing 1 (Tmbim1), mRNA [NM_001007713] | NM_001007713 |
| **1.10** | 2.14 | Increase | 2.12 | 0.0475309 | 0.4436 | doublesex and mab-3 related transcription factor 2 (predicted) | PREDICTED: Rattus norvegicus doublesex and mab-3 related transcription factor 2 (predicted) | XM_219927 |

| | | | | | | | | |
|---|---|---|---|---|---|---|---|---|
| | | | | | | | (Dmrt2_predicted), mRNA [XM_219927] | |
| **1.07** | 2.10 | Increase | 2.11 | 0.04763449 | 0.4442 | olfactory receptor 1423 (predicted) | Rattus norvegicus olfactory receptor 1423 (predicted) (Olr1423_predicted), mRNA [NM_001000006] | NM_001000006 |
| **-1.42** | 2.67 | Decrease | -2.11 | 0.04788666 | 0.4451 | filamin A interacting protein 1 | Rattus norvegicus filamin A interacting protein 1 (Filip1), mRNA [NM_145682] | NM_145682 |
| **-1.01** | 2.01 | Decrease | -2.11 | 0.04803214 | 0.4454 | Trp53bp1_predicted | PREDICTED: Rattus norvegicus transformation related protein 53 binding protein 1 (predicted) (Trp53bp1_predicted), mRNA [XM_215812] | XM_215812 |
| **1.59** | 3.01 | Increase | 2.11 | 0.04811401 | 0.4457 | AI045333 | AI045333 UI-R-C1-kh-e-06-0-UI.s1 UI-R-C1 Rattus norvegicus cDNA clone UI-R-C1-kh-e-06-0-UI 3', mRNA sequence [AI045333] | AI045333 |
| **1.09** | 2.13 | Increase | 2.11 | 0.04834112 | 0.4461 | colony stimulating factor 1 (macrophage) | Rattus norvegicus colony stimulating factor 1 (macrophage) (Csf1), mRNA [NM_023981] | NM_023981 |
| **-1.84** | 3.58 | Decrease | -2.11 | 0.04834792 | 0.4461 | AW914294 | AW914294 EST345598 Normalized rat brain, Bento Soares Rattus sp. cDNA clone RGIAD11 5' end, mRNA sequence [AW914294] | AW914294 |
| **1.28** | 2.43 | Increase | 2.11 | 0.04839854 | 0.4461 | similar to calmodulin-like 4 | Rattus norvegicus similar to CLN6 protein (LOC315746), mRNA [XM_236325] | XM_236325 |
| **1.50** | 2.82 | Increase | 2.11 | 0.0484262 | 0.4463 | aryl hydrocarbon receptor nuclear translocator-like | Rattus norvegicus aryl hydrocarbon receptor nuclear translocator-like (Arntl), mRNA [NM_024362] | NM_024362 |
| **1.02** | 2.03 | Increase | 2.10 | 0.04871161 | 0.4469 | ribonuclease, RNase A family 4 | Rattus norvegicus ribonuclease, RNase A family 4 (Rnase4), mRNA [NM_020082] | NM_020082 |

| | | | | | | | | |
|---|---|---|---|---|---|---|---|---|
| **1.18** | 2.27 | Increase | 2.10 | 0.04917496 | 0.4481 | XM_345721 | Rattus norvegicus similar to KIAA1912 protein (LOC366726), mRNA [XM_345721] | XM_345721 |
| **-1.16** | 2.23 | Decrease | -2.10 | 0.0493999 | 0.4485 | AA874941 | AA874941 UI-R-E0-ci-d-06-0-UI.s1 UI-R-E0 Rattus norvegicus cDNA clone UI-R-E0-ci-d-06-0-UI 3' similar to gi [AA874941] | AA874941 |
| **1.20** | 2.29 | Increase | 2.09 | 0.04969242 | 0.4488 | reversion-inducing-cysteine-rich protein with kazal motifs (predicted) | PREDICTED: Rattus norvegicus reversion-inducing-cysteine-rich protein with kazal motifs (predicted) (Reck_predicted), mRNA [XM_233371] | XM_233371 |

**Supplementary Material Table S4.** *STRING (a database of known and predicted protein-protein interactions) analysis of differentially expressed genes from HF/SD and HF/RD animals at adolescence.* 499 nodes were identified, with an average local clustering coefficient of 0.299 and a PPI enrichment p-value $<1 \cdot 10^{-16}$. Of these, eight main clusters were identified with the highest confidence for interaction score (≥0.9).

| | |
|---|---|
| Cluster 1 | Mrps15, Rps25, Rpsa, Fau, Rps4x, Mrps2, Mrps5, Mrps10, Rps5, Rps15a, Rps15, Rps16, *RGD1566136*, Mrps9, Mrpl43, Rps11, Mrps15. |
| Cluster 2 | Actr2, Arpc4, Epn2, Baiap2, Nckap1l, Actr3, Synj1, Ston2 |
| Cluster 3 | Pf4, Sparc, Procr, Grm2, Fpr1, C5ar1, Gpsm1, Ccl21, Gabbr1 |
| Cluster 4 | Slu7, Poldip3, Ranbp2, Dhx9, Srrm1, Bud31, Elavl1, Ddx46, Sf3b1 |
| Cluster 5 | Agtr1a, Cck, P2ry6, Tacr2, P2ry1 |
| Cluster 6 | RT1-Da, RT1-Bb, Ap1s1, RT1-Ba |
| Cluster 7 | Cyba, Ncf1, Ncf4, Mmp2 |
| Cluster 8 | Col1a1, Col6a2, Col11a2 |

**Supplementary Material Table S5.** *Top 10 KEGG pathways identified by STRING from the analysis of differentially expressed genes from HF/SD and HF/RD animals at adolescence.*

| Pathway Description | False discovery rate (FDR) | Matching proteins |
|---|---|---|
| ECM-receptor interaction | 8.95E-07 | Cd44,Chad,Col11a2,Col1a1,Col1a2,Col5a1,Col6a2,Comp,Gp9,Itga7,Lamb2,Lamb3,Spp1,Thbs2 |
| Staphylococcus aureus infection | 7.26E-05 | C1qa,C1qc,C1s,C2,C5ar1,Fpr1,RT1-Ba,RT1-Bb,RT1-Da |
| Focal adhesion | 0.00375 | Akt1,Chad,Col11a2,Col1a1,Col1a2,Col5a1,Col6a2,Comp,Itga7,Lamb2,Lamb3,Mylpf,Sos2,Spp1,Thbs2 |
| Renin-angiotensin system | 0.00386 | Agtr1a,Anpep,Cma1,Cpa3,Mcpt8l3 |
| Phagosome | 0.00408 | Atp6v1g2,Comp,Ctss,Cyba,Mrc1,Ncf1,Ncf4,RT1-A2,RT1-Ba,RT1-Bb,RT1-Da,Tfrc,Thbs2 |
| Intestinal immune network for IgA production | 0.011 | Cd80,Cxcr4,RT1-Ba,RT1-Bb,RT1-Da,Tnfsf13 |
| Systemic lupus erythematosus | 0.0159 | C1qa,C1qc,C1s,Cd80,Mcpt8l3,RT1-Ba,RT1-Bb,RT1-Da |
| PI3K-Akt signaling pathway | 0.0289 | Akt1,Chad,Col11a2,Col1a1,Col1a2,Col5a1,Col6a2,Comp,Csf1, Itga7,Lamb2,Lamb3,Osmr,Sos2,Spp1,Thbs2,Ywhab |
| Leukocyte transendothelial migration | 0.0314 | Cldn11,Cldn23,Cxcr4,Cyba,Jam2,Mmp2,Mylpf,Ncf1,Ncf4 |
| Osteoclast differentiation | 0.0402 | Akt1,Csf1r,Ctsk,Cyba,Fosb,Jund,Ncf1,Ncf4,Tnfrsf11b |
